# Cell-free DNA fragmentomics for preeclampsia risk assessment

**DOI:** 10.1038/s41467-026-72682-4

**Published:** 2026-05-02

**Authors:** Wenqiu Xu, Songchang Chen, Jia Li, Si Zhou, Yanning Yin, Zhixu Qiu, Jianguo Zhang, Cong Liu, Qiang Zhao, Gefei Xiao, Yan Zhou, Zhiguang Zhao, Xiao Zhang, Wenzhi Yang, Yunfang Wang, Huiqin Li, Zhen Yang, Suihua Feng, Qun Zhang, Weiping Chen, Huahua Li, Xiaohong Ruan, Hua Li, Sufen Zhang, Liqing Hu, Jie Qin, Wuyan Huang, Zhongzhe Li, Xianling Cao, Xuanyou Zhou, Naixin Xu, Dongxia Hou, Hong Dong, Jie Wang, Yaxian Liu, Quanfu Zhang, Xiaohua Wang, Lijian Zhao, Hefeng Huang, Chenming Xu

**Affiliations:** 1 Hebei Industrial Technology Research Institute of Genomics in Maternal & Child Health, Clin Lab, BGI Genomics, Shijiazhuang, China; 2 Institute of Reproduction and Development, Shanghai Key Laboratory of Reproduction and Development, Obstetrics and Gynecology Hospital, Fudan University, Shanghai, China; 3 Shanghai Key Laboratory of Female Reproductive Endocrine Related Diseases, Obstetrics and Gynecology Hospital, Fudan University, Shanghai, China; 4 BGI Genomics, Shenzhen, China; 5 School of Public Health, Hebei Medical University, Shijiazhuang, China; 6 Department of Obstetrics and Gynecology, Jiangmen Central Hospital, Jiangmen, Guangdong province China; 7 Clinical Transformation and Application Key Lab for Obstetrics and Gynecology, Pediatrics, and Reproductive Medicine of Jiangmen, Jiangmen, Guangdong province China; 8 Department of Medical Genetics and Prenatal Diagnosis, Zhuhai Center for Maternal and Child Health Care, Zhuhai, Guangdong province China; 9 Inner Mongolia Maternity and Child Health Care Hospital, Hohhot, China; 10 Clin Lab, BGI Genomics, Shanghai, China; 11 Department of Prevention and Health Care, Zhuhai Center for Maternal and Child Health Care, Zhuhai, Guangdong province China; 12 Shenzhen Baoan Women’s and Children’s Hospital, Shenzhen, Guangdong Province China; 13 Medical Technology College, Hebei Medical University, Shijiazhuang, China

**Keywords:** Disease model, Predictive markers, Risk factors

## Abstract

Preeclampsia (PE) is a pregnancy-specific hypertensive disorder that could lead to serious maternal and fetal complications, yet early identification of women at risk remains challenging because reliable biomarkers are limited. Here we show that generating relatively stable cell-free DNA (cfDNA) fragmentomic metrics, including transcription start site (TSS) coverage, TSS score, and Gini coefficient, required 600 million whole-genome sequencing reads of plasma cfDNA. These metrics exhibited observable differences among genes with varying expression levels in blood cells and placental tissues. In a cohort of 1,058 pregnant women, cfDNA fragmentomics could distinguish pregnancies that subsequently developed PE. When integrated with maternal risk factors, predictive models in two independent test sets achieved mean area under the curves of 0.903 and 0.850 for early-onset and late-onset PE, respectively, with sensitivities of 0.731 and 0.607 at a 10% false positive rate. Importantly, these models also performed well in samples collected before or at 16 weeks of gestation, supporting the potential of cfDNA fragmentomics in early PE risk assessment.

## Abstract (web summary)

Preeclampsia is a serious pregnancy complication, but early identification of women at risk remains difficult due to limited biomarkers. Here, the authors show that cell-free DNA fragmentomics from maternal blood can predict preeclampsia risk early in pregnancy.

## Introduction

Preeclampsia (PE) is a hypertensive disorder of pregnancy characterized by new-onset hypertension with proteinuria or organ injury occurring after 20 weeks of gestation. It poses a major threat to maternal and fetal health globally. In China, the overall prevalence of PE is 2.3%, comparable to or lower than rates in other countries^1,2^. However, the risk of adverse neonatal outcomes, particularly stillbirth, surpasses that of many other countries^1,3^. Preterm delivery, as the primary cause of poor neonatal outcomes, remains the only effective intervention for managing PE, albeit at the cost of an increased preterm birth rate. The burden of PE underscores the critical need for proactive measures. Currently, randomized trials have demonstrated that administering low-dose aspirin before 16 weeks of gestation could reduce the incidence of PE and the likelihood of preterm birth^4,5^. These findings emphasize the importance of early diagnosis and timely intervention to prevent the morbidity and mortality associated with PE, ensuring improved outcomes for pregnant women and newborns. Furthermore, according to the time of symptom onset (34 weeks of gestational age), PE is classified into two categories: early-onset and late-onset PE^3,6^. The etiologies of early-onset and late-onset PE exhibit heterogeneity^3^, leading to variable predictive performance of clinical risk factors and biomarkers. To address this, we divided pregnant women into early-onset or late-onset PE groups, aiming to identify relevant cfDNA fragmentomic biomarkers and develop effective prediction models for each group.

**Fig. 1:**
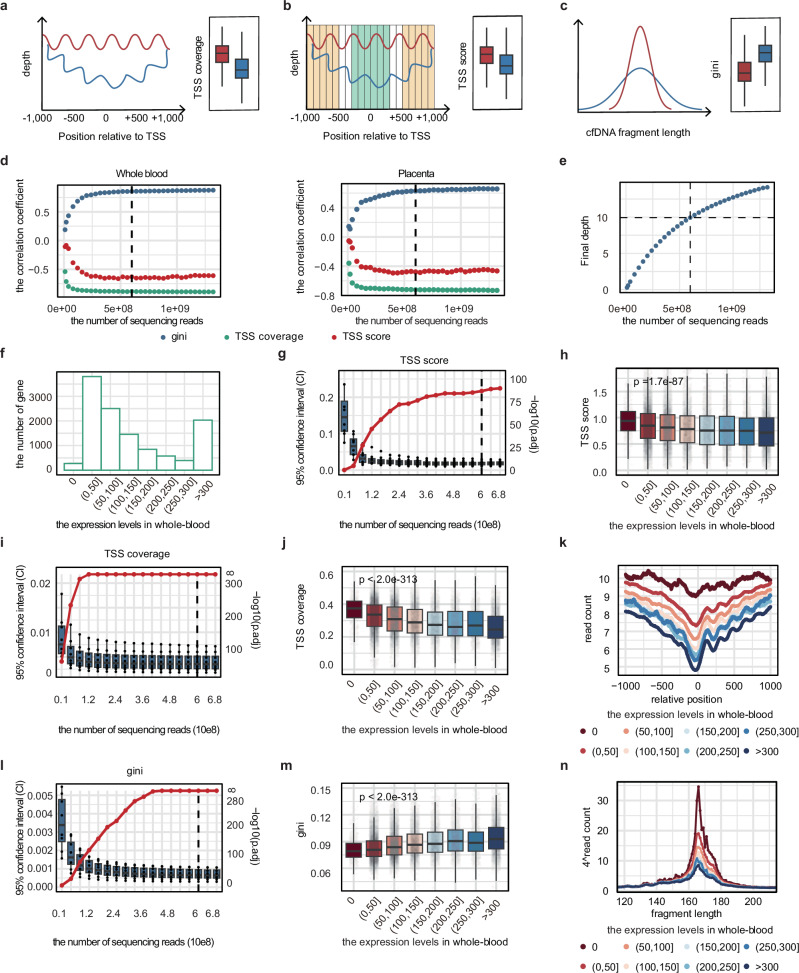
The relationship between cfDNA fragmentomics and gene expression in blood cells. **a–c** The box plots illustrate the results of three different cfDNA fragmentomic metrics: TSS coverage, TSS score and Gini coefficient (“Methods”). These metrics were used to assess overall coverage patterns (**a**) and size distributions (**c**) of cfDNA fragments at 2 kb TSS regions of active (right box) and inactive genes (left box), as shown in the left schematic illustration. 2410 active genes and 666 inactive genes were classified by their gene expression levels in blood cells (0 or >300). For TSS score calculation, each 2 kb TSS region was divided into 20 bins of 100 bp size. In panel (**b**) the distal and proximal shaded areas represent the 10 bins furthest from the TSS and 6 bins closest to the TSS, respectively. The TSS score for each TSS region was computed as the ratio of the mean read depth in the proximal bins to that in the distal bins. **d** Spearman correlation coefficients between each cfDNA fragmentomic and corresponding gene expression levels in blood cells and placentas at different sequencing depths. **e** A scatterplot depicting the relationship between the number of sequencing reads and the final read depth used for calculating three types of cfDNA fragmentomics. **f** The number of expressed genes across eight groups, categorized by gene expression levels in blood cells (0, >0 and <=50, >50 and <=100, >100 and <=150, >150 and <=200, >200 and <=250, >250 and <=300, >300). Gene expression levels were obtained using microarray analysis and preprocessed for background correction and normalization. **g** The distributions and differences of TSS scores within each gene group and among the eight gene groups as sequencing depth increased. Boxplots depict the 95% confidence interval (CI) of TSS scores within each gene group (left y-axis), while line plots depict the significance of differences in TSS scores among the eight groups using the Kruskal-Wallis test (right y-axis). **h** Boxplots showing TSS scores for different gene groups at 600 million sequencing reads. **i** Similar to (**g**) box and line plots depict the distributions and differences in TSS coverages within each gene group (left y-axis) and among the eight groups of genes (right y-axis), respectively. **j** Similar to (**h**) boxplots depict TSS coverages for different gene groups at 600 million sequencing reads. **k** Dot and line plots depict the mean number of mapped reads per base at the 2 kb TSS regions of different gene groups. **l** Similar to (**g**, **i**) box and line plots depict the distributions and differences in Gini coefficients within each gene group (left y-axis) and among the eight groups of genes (right y-axis), respectively. **m** Similar to (**h**, **j**), boxplots depict Gini coefficients for different gene groups at 600 million sequencing reads. **n** Dot and line plots depict the length distribution of mapping reads at 2 kb TSS regions of different gene groups. The dashed lines in (**d**, **e**, **g**, **i**, **l**) represent the results at 600 million sequencing reads. The Kruskal-Wallis Test was used to compare the significance of differences between three or more groups, and adjusted *p*-values were corrected using the Benjamini-Hochberg (BH) method for multiple comparisons. Box plots (**a**–**c**, **g–j**, **l**, **m**) show the median (center line, 50th percentile), with box bounds representing the 25th (first quartile, Q1) and 75th (third quartile, Q3) percentiles. Whiskers extend from the minimum to maximum values within Q1–1.5 $\times$IQR and Q3 + 1.5 $\times$IQR, where IQR is the interquartile range. Source data are provided as a Source Data file.

Plasma cell-free DNA (cfDNA) molecules, released from apoptotic and necrotic cells, were first discovered in 1948^7^. Following enzymatic processing, cfDNA fragments in plasma are predominantly composed of nucleosome-protected DNA, and their read depth and size distribution at transcription start site (TSS) regions indirectly reflect the expression signatures of tissues or blood cells in individuals^8–11^. These cfDNA fragmentomic patterns can change under specific physiological or pathological conditions and are quantifiable through fragmentomic methods^8,9,12–17^. In pregnancy, cfDNA promoter profiling has been explored for predicting pregnancy complications^10,18^, as cfDNA levels derived from active promoters in tissues or blood cells are lower than those from inactive promoters due to nucleosome occupancy. However, several critical questions remain unanswered: 1) It is unclear whether other cfDNA fragmentomic features could also be used for PE prediction. While TSS coverage, which quantifies cfDNA fragment abundance at promoters, has been characterized in previous cfDNA profiling studies^10,11^, this type of cfDNA fragmentomic has not been thoroughly explored for differences in cfDNA levels in plasma at various distances from the TSS. TSS score^19^ addresses this gap and has proven valuable in cancer diagnosis. Additionally, we reasoned that cfDNA fragments at the TSS regions of active genes are more likely to undergo random cleavage, resulting in a broader and more heterogeneous fragment size distribution. In contrast, fragments at TSS regions of inactive genes tend to show less variation due to nucleosome packaging, leading to more homogeneous fragment sizes. To capture this variation, we used the Gini coefficient to quantify the diversity of cfDNA fragment lengths at each 2 kb TSS region, reflecting the variability in fragment size distributions^11^. Thus, to capture these complexities, we employed TSS coverage, TSS score, and Gini coefficient to assess the abundance and distribution of cfDNA fragments at each TSS region, as well as the diversity of cfDNA fragment lengths (Fig. 1a–c, “Methods”). 2) The sequencing depth of cfDNA whole genome sequencing (WGS) required for relatively stable evaluation of cfDNA fragmentomic metrics has not yet been clearly defined. The fragmentation profile of cfDNA in plasma ideally is the result of various biological processes, including apoptosis and degradation. In pregnancies, the majority of cfDNA molecules originate from blood cells and the placenta. However, technical or unexplained biological factors might also influence the quantification of cfDNA fragmentomics. Therefore, it is essential to determine a reasonable sequencing depth for WGS in cfDNA fragmentomics analysis. Collectively, to address these gaps, we first compared the relationships between gene expression levels in placentas or blood cells and cfDNA fragmentomics at different sequencing depths, aiming to determine a reasonable depth for cfDNA WGS. Subsequently, we performed WGS (> 600 million sequencing reads) of plasma cfDNA from 551 healthy pregnant women, 138 pregnant women with early-onset PE (< 34 weeks of gestational age) and 369 pregnant women with late-onset PE, and evaluated the performance of PE prediction models based on diverse cfDNA fragmentomics.

## Results

### A sequencing depth of at least 600 million reads is required for robust cfDNA fragmentomics analysis

To ascertain a reasonable sequencing depth required for WGS in analyzing three types of cfDNA fragmentomics (Fig. 1a–c, “Methods”), including TSS coverage, TSS score and Gini coefficient (metrics of cfDNA fragment abundance, distribution at each TSS region and fragment length diversity), we performed WGS (approximately 50$\times$) of plasma cfDNA from a pregnant woman. Subsequently, we correlated these cfDNA fragmentomics with gene expression levels in the placenta and blood cells at different depths. Notably, as the number of cfDNA sequencing reads increased, correlations between gene expression levels in blood cells and corresponding TSS coverages, TSS scores, or Gini coefficients progressively improved and appeared to plateau when sequencing depth reached approximately 600 million reads (Fig. 1d). Similar results were also observed when comparing with expression levels in placental tissues (Fig. 1d, Supplementary Data 1). Based on this analysis, we supposed that calculations of cfDNA fragmentomics, including TSS coverages, TSS scores, and Gini coefficients, began to stabilize at a final read depth of 10$\times$, which was obtained from 600 million sequencing reads (Fig. 1e).

To validate these observations, we further compared the differences in TSS coverages, TSS scores, and Gini coefficients among eight groups of genes categorized by their expression levels in blood cells or placental tissues (Fig. 1f, Supplementary Fig. 1a and Supplementary Data 2). We reasoned that with increasing sequencing depth, cfDNA fragmentomic features would exhibit more consistent results within each gene group and more distinct differences among the eight groups. Indeed, at 600 million sequencing reads, we observed narrow 95% confidence intervals (CIs) for TSS coverages, TSS scores and Gini coefficients within each group and more significant differences in these features among eight groups (Fig. 1g, i, l, Supplementary Fig. 1b, d, g). Genes with higher expression levels in blood cells or placental tissues exhibited lower cfDNA levels within 2 kb TSS regions, particularly in subregions closest to the TSS (Fig. 1h, j–k, Supplementary Fig. 1c, e, f). Poorly expressed genes exhibited a sharp peak in the distribution of cfDNA fragment lengths and low diversity within 2 kb TSS regions (Fig. 1m, n, Supplementary Fig. 1h, i.). Overall, these results indicated that at a sequencing depth of 600 million reads ( ~10 $\times$ final read depth), TSS coverage, TSS score, and Gini coefficient metrics showed modest but observable differences between genes with different expression levels in tissues and blood.

### CfDNA-fragmentomic features associated with PE

**Fig. 2:**
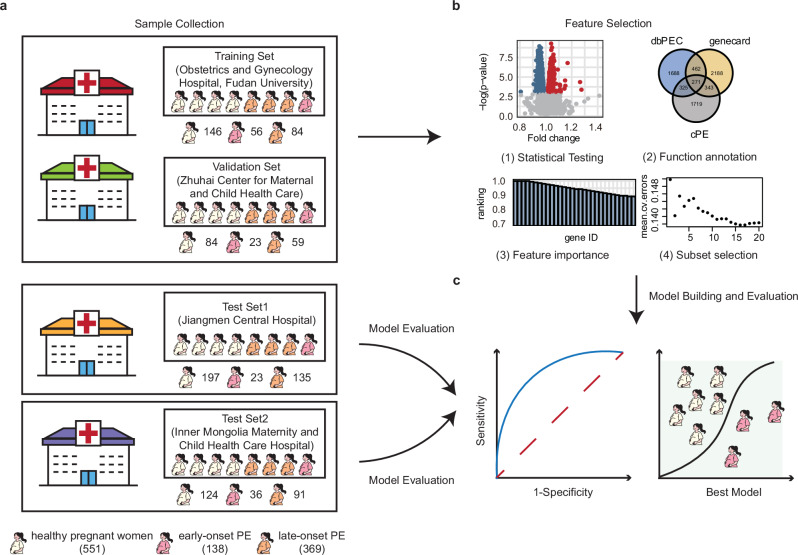
Procedure for predicting PE by cfDNA fragmentomics. **a** Sample collection. Maternal plasma samples were collected from pregnant women (gestational ages between 12 and 26 weeks) at four hospitals in China. According to the recruitment criteria (“Methods”), the study included samples from 551 healthy pregnant women, 138 pregnant women with early-onset PE, and 369 pregnant women with late-onset PE. Samples from each hospital were analyzed as independent datasets. For subsequent feature selection, training and validation sets were combined to select features for each cfDNA fragmentomic. **b** Feature selection. The workflow for feature selection is depicted in the schema, including statistical testing, function annotation, ranking feature importance, and identifying the optimal subset of features. **c** Model building and evaluation. Prediction models for early-onset PE or late-onset PE were developed using the training set. Model performance was then evaluated using the validation and test sets.

**Fig. 3:**
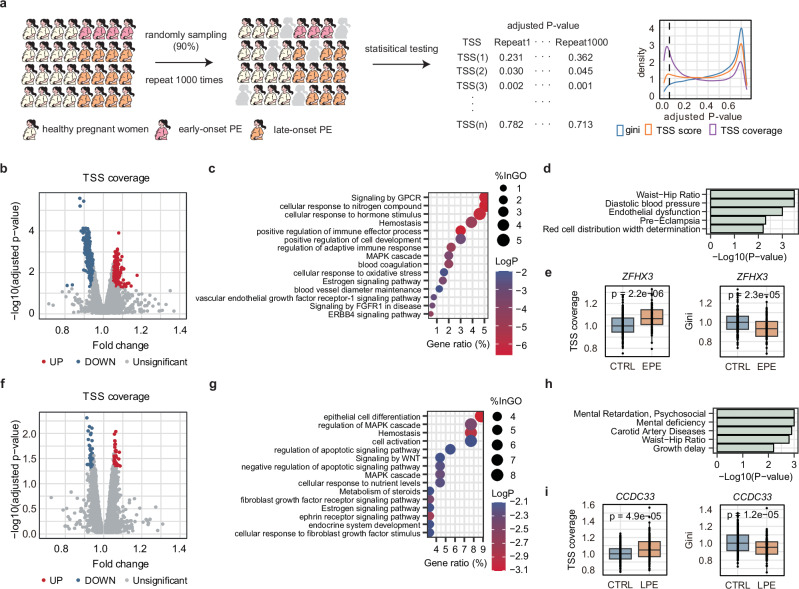
Differential cfDNA fragmentomics analysis between early-onset or late-onset PE and healthy controls. **a** Workflow overview. The schema illustrates the workflow for the differential analysis of cfDNA fragmentomics. For early-onset PE, the differential analysis of three types of cfDNA fragmentomics was performed using randomly selected 90% of healthy samples and 90% of samples with early-onset PE. This process was repeated 1000 times. For each feature of each cfDNA fragmentomic, the upper 95% CI of the adjusted *p*-values from 1000 differential analyses was used as the selection criterion. A similar approach was applied to identify differential features for late-onset PE prediction. The dashed line represents the threshold where the upper 95% CI of the adjusted *p*-values equals 0.05. **b**, **f** Differential TSS coverages between PE samples and controls. TSS regions with significantly higher and lower TSS coverages in early-onset PE (**b**) or late-onset PE (**f**) were highlighted across 1000 differential analyses, with the upper 95% CI of adjusted *p*-values less than 0.05. Fold changes in TSS coverages between PE samples and controls were averaged over 1000 analyses. Adjusted *p*-values were obtained by the two-sided Wilcoxon rank sum test and corrected using the Benjamini-Hochberg (BH) method for multiple comparisons. **c**, **g** Enrichment analysis. Enrichment analysis of pathways and biological processes was performed on genes associated with differential TSS coverages identified in early-onset PE (**c**) or late-onset PE samples (**g**). **d**, **h** DisGeNET analysis. DisGeNET analysis of genes with differential TSS coverages between early-onset PE (**d**) or late-onset PE samples (**h**) and controls identified in (**b**, **f**). *P*-values for functional enrichment were calculated using the accumulative hypergeometric test in Metascape. **e**, **i** Boxplots depict TSS coverages and Gini coefficients at TSS regions of specific genes enriched for “Diastolic blood pressure” in the DisGeNET database. For example, *ZFHX3* (**e**) and *CCDC33* (**i**) genes showed significant differences in early-onset PE or late-onset PE samples compared to controls. *P*-values in boxplots were obtained via the two-sided Wilcoxon rank sum test with no correction for multiple comparisons. Panel (**e**) includes 79 early-onset PE samples (EPE) and 230 healthy control samples (CTRL), while panel (**i**) includes 143 late-onset PE samples (LPE) and 230 healthy control samples. Box plots (**e**, **i**) show the median (center line, 50th percentile), with box bounds representing the 25th (first quartile, Q1) and 75th (third quartile, Q3) percentiles. Whiskers extend from the minimum to maximum values within Q1-1.5 $\times$IQR and Q3 + 1.5 $\times$IQR, where IQR is the interquartile range. Source data are provided as a Source Data file.

To assess the potential of cfDNA fragmentomics in predicting early-onset and late-onset PE, we performed WGS ( > 10$\times$ final read depth) of plasma cfDNA from pregnant women with PE (*n* = 138 for early-onset PE, *n* = 369 for late-onset PE) and sampling-gestation-matched healthy controls (*n* = 551). As we established that a final read depth of at least 10$\times$ is required for relatively stable estimation of cfDNA fragmentomics, all data were downsampled to 10$\times$ to ensure consistency across the following analyses (Supplementary Fig. 2a–d, Supplementary Data 3). These samples were collected from four different hospitals (Fig. 2, Supplementary Fig. 2, Table 1, Supplementary Data 3 and Supplementary Table 1–3). Early-onset, late-onset PE and healthy samples from two hospitals were designated as the feature-selection set to identify features for each cfDNA fragmentomic that could facilitate distinguishing pregnant women with early-onset or late-onset PE from healthy controls, respectively. The feature-selection set comprised 79 pregnant women with early-onset PE, 143 pregnant women with late-onset PE and 230 healthy participants (Fig. 2). We carried out differential analyses through 1000 bootstrap resamplings (Fig. 3a), retaining 985, 87 and 2585 TSS regions with significantly differential TSS coverages, TSS scores and Gini coefficients between pregnant women with early-onset PE and controls, respectively (Fig. 3b, Supplementary Fig. 3a, e, “Methods”). Similarly, 107, 63 and 1924 TSS regions were retained as significantly differential TSS coverages, TSS scores, and Gini coefficients in late-onset PE samples compared with healthy controls (Fig. 3f, Supplementary Fig. 4a, e).

**Table 1:** Maternal characteristics of early-onset PE and healthy pregnancies in the training and validation sets

	Training Set			Validation Set		
	Control (*n* = 146)	EPE (*n* = 56)	*P*-value	Control (*n* = 84)	EPE (*n* = 23)	*P*-value
Maternal age (years)	30.00 $\pm$3.02	32.50 $\pm$4.41	3.22e-7	30.00 $\pm$3.76	34.00 $\pm$5.18	1.00e-3
**Gestational age**						
at sampling (weeks)	16.10 $\pm 1.71$	15.35 $\pm 1.66$	0.06	15.80 $\pm 2.56$	15.60 $\pm 2.78$	0.73
at delivery (weeks)	39.20 $\pm 0.87$	32.70 $\pm 2.22$	3.89e-28	39.00 $\pm 0.91$	33.50 $\pm$4.83	9.22e-12
at onset (weeks)	−	31.55 $\pm$1.83	−	−	29.60 $\pm$3.40	−
Height (cm)	163.00 $\pm$5.21	162.00 $\pm$5.32	0.24	160.00 $\pm$5.15	158.00 $\pm$6.16	0.08
Weight (kg)	55.00 $\pm$6.74	60.05 $\pm$10.53	3.67e-6	55.00 $\pm$7.49	58.00 $\pm$8.42	0.03
BMI (kg/m^2^)	20.33 $\pm$2.26	23.37 $\pm$3.98	1.04e-7	21.25 $\pm$2.58	23.31 $\pm$3.43	3.02e-3
MAP	82.98$\pm$10.28	94.65 $\pm$10.05	6.07e-10	81.46 $\pm$8.83	95.35 $\pm$10.32	4.89e-4
Past medical history	0 (0.00%)	6 (10.71%)	5.88e-4	0 (0.00%)	3 (13.04%)	0.01
**Conception method**						
In vitro fertiliaztion	9 (6.16%)	14 (25.00%)	3.25e-3	1 (1.19%)	2 (8.70%)	0.13
Natural	137 (93.84%)	42 (75.00%)	0.35	83 (98.81%)	21 (91.30%)	0.87
**Parity**						
>0	55 (37.67%)	8 (14.29%)	0.02	29 (34.52%)	11 (47.83%)	0.51
>1	3 (2.05%)	1 (1.79%)	1.00	3 (3.57%)	1 (4.35%)	1.00
**Gravidity**						
>1	78 (53.42%)	11 (19.64%)	3.97e-3	37 (44.05%)	15 (65.22%)	0.33
>2	31 (21.23%)	4 (7.14%)	0.06	14 (16.67%)	9 (39.13%)	0.11

Data were shown as media ± SD values of clinical characteristics or numbers (percentages) of samples. *EPE* early-onset preeclampsia, *BMI* body mass index, *MAP* mean arterial pressure. The two-sided Fisher’s exact test was used to compare gravidity, parity, past medical history and method of conception between PE samples and healthy controls. The two-sided Wilcoxon rank sum test was utilized to compare maternal age, BMI, and MAP between PE samples and healthy controls. Source data are provided as a Source Data file.

Next, to further understand these changes in cfDNA fragmentomics, we performed enrichment analyses on corresponding gene sets associated with differential TSS coverages, TSS scores, or Gini coefficients between pregnant women with early-onset or late-onset PE and healthy controls. Notably, several well-known PE-associated pathways were enriched in early-onset or late-onset PE, including “Hemostasis”^20^, “cellular response to oxidative stress”^21^, and “VEGFA-VEGFR2 Pathway”^22^ (Fig. 3c, g, Supplementary Fig. 3b, f, Supplementary Fig. 4b, f and Supplementary Data 4–9). Additionally, gene sets associated with differential cfDNA fragmentomic features were enriched in diseases such as “Diastolic blood pressure” and “Mean blood pressure” within the DisGeNET database (Fig. 3d, h, Supplementary Fig. 3c, g, Supplementary Fig. 4c, g, and Supplementary Data 4–9), consistent with the high blood pressure as a typical feature of PE. Notably, distinct cfDNA fragmentomic features of genes such as *ZFHX3*, *CCDC33*, *PLEKHH1*, and *PRKCE*, which are associated with “Diastolic blood pressure”, exhibited opposing trends in early-onset or late-onset PE, as indicated by their TSS coverages and Gini coefficients (Fig. 3e, i, Supplementary Fig. 3h and Supplementary Fig. 4h). Similarly, TSS scores and Gini coefficients of *NT5C2* and *HDAC4* genes showed different directional changes between early-onset or late-onset PE and healthy controls (Supplementary Fig. 3d, Supplementary Fig. 4d). These observations supported our hypothesis that specific TSS regions in pregnant women with PE exhibited increased cfDNA fragment abundance and reduced length diversity, reflecting a dynamic regulation of cfDNA fragmentomics in response to pathways associated with PE. These changes also highlighted the potential value of cfDNA fragmentomic features as predictive biomarkers for PE.

### Identification of cfDNA fragmentomic features for PE prediction

**Fig. 4:**
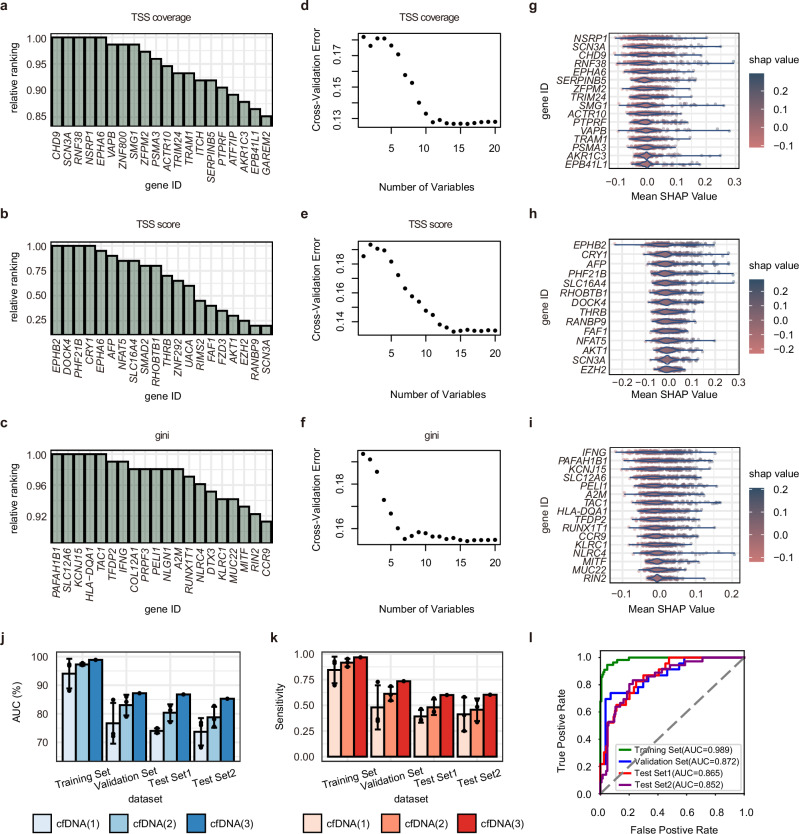
Feature selection for each cfDNA fragmentomic and early-onset PE prediction. **a–c** Corresponding gene ranking for top 20 TSS coverages (**a**), TSS scores (**b**), and Gini coefficients (**c**) according to their feature importance for each cfDNA fragmentomic. **d–f** Dot plots depict the mean squared error across each fold during the cross-validation process for models built with distinct subsets of predictors (“Methods”). The subsets of predictors, ranging in size from 1 to 20, were derived from the top 20 TSS coverages, TSS scores, and Gini coefficients as shown in **a–c**. **g–i** The SHAP summary plots depict the distribution of contributions of TSS coverages (**g**), TSS scores (**h**), and Gini coefficients (**i**) to early-onset PE prediction, respectively. The y-axis represented genes corresponding to TSS coverages, TSS scores or Gini coefficients of the optimal subsets in (**d–f**). **j** The barplots depict the mean area under the curve (AUC) of early-onset PE models based on different combinations of cfDNA fragmentomic types in the training set, validation set and test sets. **k** The barplots depict the mean sensitivity of early-onset PE models based on different combinations of cfDNA fragmentomic types in the training set, validation set, and test sets at a false positive rate of 10%. The error bar plots in (**j**, **k)** represent the mean ± SD of AUCs or sensitivities for models constructed using one, two, or all three types of cfDNA fragmentomics. The number of models constructed using one, two, and all three types of cfDNA fragmentomics is three, three, and one, respectively. Specifically, “cfDNA (1)”, “cfDNA (2)”, and “cfDNA (3)” indicate models that incorporate one, two, and three fragmentomics types, respectively. The “cfDNA (2)” group includes three models built with two types of cfDNA fragmentomics: 1) 16 TSS coverages and 14 TSS scores, 2) 16 TSS coverages and 16 Gini coefficients, 3) 14 TSS scores and 16 Gini coefficients. Similarly, “cfDNA (1)” includes models using each individual fragmentomic type separately, and “cfDNA (3)” refers to models that combine all three types. The detailed performance metrics of each model are provided in Supplementary Data 16. **l** ROC curves of the optimal early-onset PE model based on cfDNA fragmentomics in the training set, validation set, and test sets. Source data are provided as a Source Data file.

To enhance the interpretability of differential cfDNA fragmentomic features, we focused on differential features corresponding to genes reported in PE-associated databases, including cPE^23^, dbPEC^24^, or GeneCards^25^. For early-onset PE prediction, 254 TSS coverages, 27 TSS scores, and 666 Gini coefficients were included, while 33 TSS coverages, 24 TSS scores, and 526 Gini coefficients were retained for late-onset PE prediction (Supplementary Fig. 5). To optimize model performance, we ranked the importance of features for each cfDNA fragmentomic using the Boruta algorithm (Fig. 4a–c, Supplementary Fig. 6a–c, Supplementary Data 10–15). The top 20 features for each cfDNA fragmentomic were then used to search for the best subset of features by tenfold cross-validation. Ultimately, 16 TSS coverages, 14 TSS scores, and 16 Gini coefficients were selected to discriminate early-onset PE from control pregnancies (Fig. 4d–f). For late-onset PE prediction, 17 TSS coverages, 17 TSS scores, and 16 Gini coefficients were incorporated into models (Supplementary Fig. 6d–f). Using SHapley Additive exPlanations (SHAP) for feature contribution analysis in tenfold cross-validation, we found that cfDNA fragmentomic features of *EPHA6* and *CHD9* genes were more influential attributes for both early-onset and late-onset PE prediction (Fig. 4g–i, Supplementary Fig. 6g–i). This suggested consistent regulatory processes in pregnancies affected by early-onset and late-onset PE.

### Evaluation of cfDNA fragmentomics for PE prediction

We next assessed the potential of selected features for each cfDNA fragmentomic in distinguishing pregnant women with PE from healthy controls during the first and second trimesters. To ensure the independence of hospital datasets, early-onset PE, late-onset PE, and healthy samples from one hospital in the feature-selection set were used as the training set, while samples from another hospital were designated as the validation set (Fig. 2a). Using the same healthy controls for both early-onset and late-onset PE models, we constructed random forest (RF) models in the training set based on each cfDNA fragmentomic. When comparing the performance of models based on different combinations of cfDNA fragmentomic types, we observed a progressive increase in the area under the curve (AUC) as the number of cfDNA fragmentomic types included in the early-onset PE prediction model increased (Fig. 4j, Supplementary Data 16). The optimal early-onset PE model was defined as the model that achieved the highest performance on the validation dataset based on a specific feature set. The optimal early-onset PE model based on cfDNA fragmentomics, which considered 46 TSS regions (16 TSS coverages, 14 TSS scores, and 16 Gini coefficients), achieved AUCs of 0.872 (95% CI: 0.869–0.875), 0.868 (95% CI: 0.866–0.870), and 0.853 (95% CI: 0.851–0.855) in the validation and test sets, respectively (Fig. 4l, Supplementary Data 16). At a 10% false positive rate (FPR), the model achieved sensitivities of 0.735 (95% CI: 0.728–0.741), 0.600 (95% CI: 0.593–0.608), and 0.603 (95% CI: 0.597–0.610) (Fig. 4k, Supplementary Data 16). Similarly, the optimal late-onset PE model, based on 33 TSS regions (17 TSS coverages and 16 Gini coefficients), showed moderate performance in the validation and test sets, with AUCs of 0.814 (95% CI: 0.812–0.817), 0.771 (95% CI: 0.769–0.773), and 0.812 (95% CI: 0.810–0.814) (Supplementary Fig. 6j, l, Supplementary Data 17). Corresponding sensitivities at a 10% FPR were 0.525 (95% CI: 0.520–0.531), 0.492 (95% CI: 0.489–0.495), and 0.471 (95% CI: 0.466–0.477) (Supplementary Fig. 6k, Supplementary Data 17). Notably, the optimal models for early-onset PE and late-onset PE prediction, developed using datasets downsampled to 10 $\times$ coverage, also achieved comparable or even improved performance when evaluated on pre-downsampled samples from test sets, suggesting generalizability across read depths greater than 10$\times$ (Supplementary Fig. 7, Supplementary Data 18, 19).

**Fig. 5:**
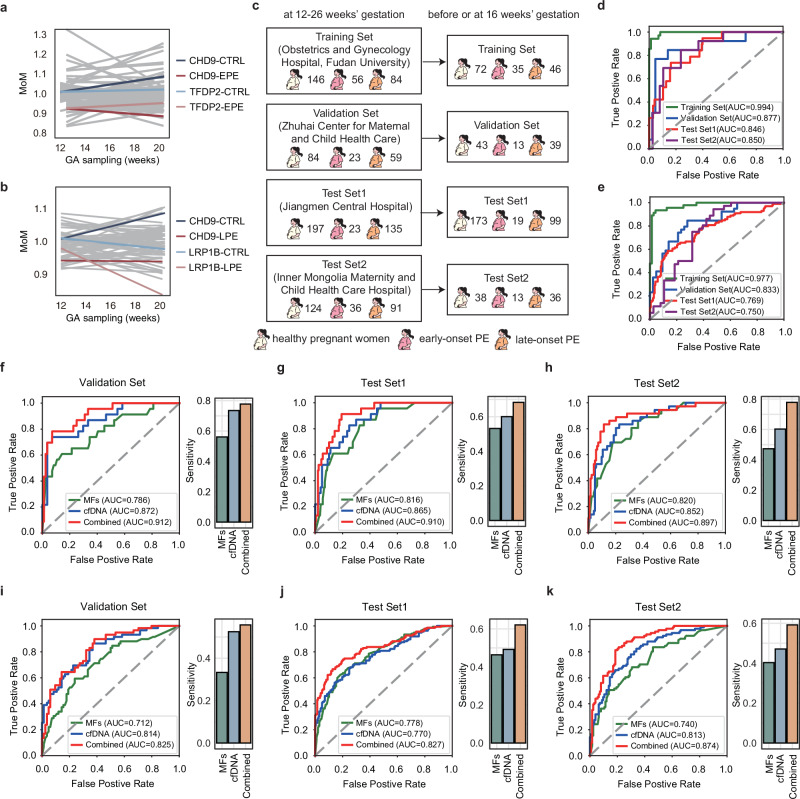
CfDNA fragmentomics improving PE prediction for samples obtained at 12–26 weeks of gestation. **a, b** The changes of cfDNA fragmentomic features used in the optimal early-onset (**a**) or late-onset (**b**) PE model as gestation progressed. The highlighted lines were two examples of feature changes over gestational weeks. GA: gestational age. **c** A schematic diagram showing the distribution of samples collected before or at 16 weeks of gestation across different hospitals. **d**, **e** ROC curves of early-onset (**d**) or late-onset (**e**) PE models constructed and validated based on cfDNA fragmentomics for samples obtained before or at 16 weeks of gestation. **f–h** The plots on the left show ROC curves of early-onset PE models based on cfDNA fragmentomics or maternal factors (MFs) for samples collected at 12–26 weeks of gestation in the validation set (**f**), test set1 (**g**), and test set2 (**h**). The barplots on the right depict sensitivities of early-onset PE models based on cfDNA fragmentomics or MFs in the validation set (**f**), test set1 (**g**), and test set2 (**h**), at a false positive rate of 10%. **i–k** The plots on the left show ROC curves of late-onset PE models based on cfDNA fragmentomics or MFs for samples collected at 12–26 weeks of gestation in the validation set (**i**), test set1 (**j**), and test set2 (**k**). The barplots on the right depict sensitivities of late-onset PE models based on cfDNA fragmentomics or MFs in the validation set (**i**), test set 1 (**j**), and test set 2 (**k**), at a false positive rate of 10%. EPE, early-onset preeclampsia. LPE, late-onset preeclampsia. CTRL, control. Source data are provided as a Source Data file.

Interestingly, we observed that many of the cfDNA fragmentomic features in the optimal early-onset or late-onset PE models did not show significant changes as gestation progressed, suggesting that these changes might occur as early as the first trimester (Supplementary Data 20, 21). For example, changes in TSS coverage of the *CHD9* gene in pregnant women with high risk of late-onset PE were detected earlier than changes in the Gini coefficient of the *LRP1B* gene, and such earlier changes in the *CHD9* and *TFDP2* genes were also observed in early-onset PE pregnancies (Fig. 5a, b). Considering the use of low-dose aspirin for PE prevention before 16 weeks of gestation, we further established and validated early-onset and late-onset PE models using samples obtained before or at 16 weeks’ gestation, utilizing the same features as described above (Fig. 5c). Only a slight decline was observed in the performance of the early-onset model, which displayed comparable performance when applied to samples obtained before or at 16 weeks’ gestation and to those collected between 12 and 26 weeks of gestation, with AUCs of 0.875 (95% CI: 0.871–0.879), 0.846 (95% CI: 0.843–0.848), and 0.851 (95% CI: 0.848–0.855) (Fig. 5d, Supplementary Data 22). Sensitivities at a 10% FPR were 0.785 (95% CI: 0.776–0.794), 0.492 (95% CI: 0.484–0.500), and 0.623 (95% CI: 0.611–0.635) in the validation and test sets, respectively (Supplementary Data 22). The late-onset PE model for samples obtained before or at 16 weeks of gestation exhibited a moderate reduction in performance compared with samples collected between 12 and 26 weeks of gestation. It yielded AUCs of 0.830 (95% CI: 0.827–0.833), 0.770 (95% CI:0.768–0.772), and 0.748 (95% CI: 0.744–0.751), with sensitivities of 0.572 (95% CI: 0.563–0.581), 0.515 (95% CI: 0.510–0.521), and 0.289 (95% CI: 0.280–0.299) at a 10% FPR across validation and test sets (Fig. 5e, Supplementary Data 23). Together, these findings highlighted the potential of cfDNA fragmentomics to identify pregnancies at risk of developing PE in the first and second trimesters.

### CfDNA fragmentomics improve PE prediction

Through the competing risks model, previous studies have assessed PE risk according to maternal characteristics in different populations^26,27^. Consistent with these findings, we also examined the contributions of maternal characteristics to PE risk assessment in our datasets. Maternal age, body mass index (BMI), mean arterial pressure (MAP), past medical history, and conception method showed significant differences between early-onset or late-onset PE and healthy controls (Table 1, Supplementary Table 1–3). To determine whether adding cfDNA fragmentomics could enhance the predictive performance of early-onset and late-onset PE models, we first reconstructed PE models based on maternal characteristics using the RF algorithm. Among the maternal factors (MFs), maternal age, BMI, and MAP showed stronger predictive performance, with higher AUCs for both early-onset and late-onset PE models compared to other maternal characteristics (Supplementary Figs. 8, 9). Using these three MFs, the early-onset PE model achieved AUCs of 0.786 (95% CI: 0.782–0.790), 0.817 (95% CI: 0.814–0.819), and 0.821 (95% CI: 0.819–0.824) in the validation and test sets (Supplementary Fig. 8f, Supplementary Data 22). Similarly, the late-onset PE model based on three MFs yielded AUCs of 0.714 (95% CI: 0.712–0.717), 0.778 (95% CI: 0.777–0.780), and 0.739 (95% CI: 0.737–0.741) in the validation and test sets (Supplementary Fig. 9f, Supplementary Data 23).

Next, we constructed combined models using cfDNA fragmentomics (46 TSS regions for early-onset PE and 33 TSS regions for late-onset PE) and MFs (maternal age, BMI and MAP). For early-onset PE, the combined model developed in the training set showed improved predictive performance in the validation and test sets, achieving AUCs of 0.912 (95% CI: 0.910–0.914), 0.910 (95% CI: 0.909–0.912), and 0.896 (95% CI: 0.894–0.898), respectively (Fig. 5f–h, Supplementary Data 22). Compared with the model based on MFs alone, the combined model demonstrated better performance (*P* = 0.013, 0.005 and 0.061 in validation and test sets, respectively), although performance differences between the combined model and the model based on cfDNA fragmentomics alone were not statistically significant (*P* = 0.138, 0.108 and 0.083). At a 10% FPR, the corresponding sensitivities were 0.778 (95% CI: 0.772–0.783), 0.683 (95% CI: 0.677–0.690), and 0.779 (95% CI: 0.772–0.785) (Fig. 5f–h, Supplementary Data 22). Importantly, performance improvements of the early-onset PE model were also observed in a subgroup of participants whose samples were collected before or at 16 weeks’ gestation, although the improvements were not statistically significant (*P*_combined vs MFs_ = 0.092, 0.103, and 0.237, and *P*_combined vs cfDNA fragmentomics_ = 0.578, 0.030, and 0.157 in validation and test sets, respectively). In this early-gestation subgroup, the combined model achieved AUCs of 0.902 (95% CI: 0.898–0.905), 0.900 (95% CI: 0.898–0.902), and 0.893 (95% CI: 0.890–0.896) in the validation and test sets, respectively. At a 10% FPR, sensitivities were 0.758 (95% CI: 0.749–0.768), 0.634 (95% CI: 0.627–0.642), and 0.709 (95% CI: 0.698–0.721) (Supplementary Fig. 10a–c, Supplementary Data 22).

Similarly, the combined late-onset PE model also showed significantly higher classification accuracy than models based on either MFs or cfDNA fragmentomics alone in the validation and test sets for samples obtained from 12 weeks of gestation onwards (*P*_combined vs MFs_ = 0.003, 0.025, and 2.643e-05, *P*_combined vs cfDNA fragmentomics_ = 0.605, 1.823e-04, and 0.003) (Supplementary Data 23). Comparable or improved performance was also observed for samples collected before or at 16 weeks of gestation (*P*_combined vs MFs_ = 0.04, 0.106, and 0.046, *P*_combined vs cfDNA fragmentomics_ = 0.679, 5.788e-04, and 0.006) (Supplementary Data 23). For samples collected between 12 and 26 weeks of gestation, the combined late-onset PE model yielded AUCs of 0.826 (95% CI: 0.824–0.828), 0.826 (95% CI: 0.825–0.828), and 0.874 (95% CI: 0.872–0.875) in validation and test sets (Fig. 5i–k, Supplementary Data 23). For samples obtained before or at 16 weeks of gestation, the corresponding AUCs were 0.847 (95% CI: 0.844–0.850), 0.822 (95% CI: 0.820–0.824), and 0.846 (95% CI: 0.843–0.849) (Supplementary Fig. 10d–f, Supplementary Data 23). At a specificity of 0.900, the combined late-onset PE model achieved sensitivities of 0.557 (95% CI: 0.551–0.562), 0.621 (95% CI: 0.618–0.625), and 0.592 (95% CI: 0.587–0.597) for samples obtained at 12–26 weeks of gestation, and 0.657 (95% CI: 0.652–0.662), 0.607 (95% CI: 0.600–0.614), and 0.551 (95% CI: 0.541–0.562) for those collected before or at 16 weeks of gestation (Supplementary Data 23). Overall, these observations suggested that integrating cfDNA fragmentomics with maternal factors could enhance the predictive performance of PE models compared with those based on individual factors alone.

## Discussion

PE remains one of the leading causes of maternal morbidity and mortality worldwide, causing at least 42,000 maternal deaths annually^28^. When determining the proper window for early identification of pregnant women at risk for PE, two key aspects should be considered. On the one hand, the initiation of low-dose aspirin intervention, which has been reported to effectively reduce the risk of PE^5^, is recommended before 16 weeks of gestation. On the other hand, it is important to consider a gestational window that enables relatively higher detection rates of PE risk. The Fetal Medicine Foundation (FMF) first-trimester prediction model, based on maternal risk factors, mean arterial pressure, uterine artery pulsatility index (UtA-PI), and serum biomarkers such as PIGF, has been externally validated in diverse prospective cohorts at 11–13 weeks of gestation and is regarded as a reliable standard for early screening of PE^29–33^. However, its predictive performance varies across populations. At a false-positive rate of 10%, detection rates for preterm PE were reported at 56.4% in Chinese and 64.0% in Asian populations^32,33^. Moreover, the application of UtA-PI, despite its potential, has not been widely adopted, particularly in China, due to its demanding and complex measurement technique. In parallel, emerging PE prediction based on cfDNA methylation has shown promise but also faces limitations. For example, a recent study by DeBorre et al. reported that cfDNA methylation profiles obtained before 12 weeks of gestation yielded lower predictive performance compared to those collected later than 12 weeks of gestation^26^, suggesting that the placental signal captured via methylation might be less robust at very early time points. While Non-invasive prenatal testing (NIPT) for fetal aneuploidy screening can be performed from ≥10 weeks of gestation, according to American College of Medical Genetics and Genomics (ACMG) guidelines^34^, in China, routine first-trimester blood tests are typically conducted between 10 and 12 weeks for maternal health assessment, and NIPT is generally performed between 12 and 26 weeks. Within this window, only a small proportion of samples were collected before 16 weeks, and restricting PE prediction to this early subset could potentially overlook many high-risk pregnancies. Given that the American College of Obstetricians and Gynecologists (ACOG) recommends starting low-dose aspirin between 12 and 28 weeks of gestation^35^, and that NIPT is typically performed during this window, it is clinically meaningful to explore whether cfDNA-based methods could provide predictive value using routinely collected NIPT samples. Therefore, in this study, we evaluated the performance of PE prediction based on cfDNA fragmentomics using samples obtained during NIPT, as well as the subset of NIPT samples collected before or at 16 weeks of gestation.

Previous and recent studies have demonstrated the feasibility of building PE prediction models using low-depth paired-end cfDNA WGS data (0.3 $\times$ or 0.5 $\times$), originally designed for fetal chromosomal abnormality screening^10,36^. While many current NIPT workflows have adopted or are transitioning toward paired-end sequencing, this approach has not been universally implemented across all regions. Single-end sequencing remains in use in some clinical laboratories, such as in China, which prevents the estimation of fragment length. In our previous work using NIPT data routinely collected for fetal aneuploidy screening in China, predictive performance across different clinical cohorts was limited (AUCs of 0.79, 0.76, and 0.60)^37^, and nucleosome positioning signals around promoter regions showed insufficient resolution. These findings motivated our exploration of fragmentomic markers at different sequencing conditions and depths. In this study, we employed a sequencing depth of at least 600 million reads ( ~ 10 $\times$ final read depth) to analyze TSS coverages, TSS scores and Gini coefficients with predictive potential for PE. At this threshold, our PE models based on MFs and cfDNA fragmentomics showed robust and consistent predictive performance across test sets. For samples collected at 12–26 weeks of gestation, the AUCs were 0.910 and 0.896 for early-onset PE, and 0.826 and 0.874 for late-onset PE. For samples collected before or at 16 weeks of gestation, the AUCs were 0.900 and 0.893 for early-onset PE, and 0.822 and 0.846 for late-onset PE. These results supported smaller differences in performance between the two test sets, especially for early-onset PE. At a 10% false positive rate, the average sensitivities for predicting early-onset and late-onset PE were 0.731 and 0.672, and 0.607 and 0.579, respectively. A 10% false positive rate might be relatively high, resulting in unnecessary anxiety and additional follow-up testing. However, this rate remains within the range observed for other clinically accepted prenatal screening tests, such as first-trimester combined screening for preeclampsia^30–33^.

Although our study explored the predictive performance of PE models based on cfDNA fragmentomics for samples collected during 12–16 weeks of gestation, with an average AUC of 0.852 in test sets, we acknowledge that this performance might appear relatively high, as clinical sampling in the participating hospitals typically occurs after 12 weeks of gestation. In contrast, De Borre et al. reported that an early-onset PE model based on cfDNA methylation alone achieved AUCs of 0.752 for samples collected between 9–14 weeks and 0.629 for those collected before 12 weeks. Their study provided valuable insights into the role of cfDNA methylation in PE prediction, especially at earlier gestational ages^26^. Additionally, PE prediction using cfRNA profiles showed AUCs of 0.710, 0.720, and 0.740 across different validation cohorts for samples collected between 5 and 16 weeks of gestation^38^. Together, these studies contributed significantly to the growing body of knowledge on earlier PE detection using various omics approaches. Importantly, we also acknowledge the recent work by Adil et al.^36^, which represents an influential contribution to cfDNA-based PE prediction. Adil et al. reported an overall AUC of 0.84 (AUC of 0.92 for EPE and 0.79 for LPE-PB) for predicting PE requiring preterm delivery based on 0.5 $\times$ paired-end sequencing data in a closely approximating real-world cohort, with samples collected at a median of 12.2 weeks (range, 8.1–16.0 weeks). Our study was in preparation and revision during the publication of their work. While our study required approximately 10 $\times$ sequencing depth, which is higher than that used in routine NIPT workflows, it explored fragmentomic markers detectable at different sequencing depths. This represents a promising complementary direction for cfDNA-based preeclampsia prediction, rather than a replacement for approaches optimized for ultra-low-depth NIPT data. We believe that, for basic research and early-stage clinical investigations, examining cfDNA fragmentomic signals at different sequencing depths remains meaningful. In addition to assessing predictive performance, higher sequencing depth allows for more detailed exploration of the underlying biological mechanisms, including the relationship between predictive markers, genetic information, and infection-related signals, which might inform future marker discovery and mechanistic studies.

Here, we observed significant differences in maternal age and BMI between pregnant women with PE and healthy controls. Higher maternal age and BMI were associated with an increased risk of PE, although similar findings had not always been consistently reported in other PE screening or prediction studies^26,31^. To ensure the clinical generalizability of our sample collection, pregnant women in the disease group were strictly selected from four hospitals based on established PE criteria, while sampling-gestation-matched controls were randomly selected. This approach ensured that differences in maternal age and BMI between the PE and control groups were not artificially introduced. Additionally, some population-based cohort studies have suggested that elevated maternal age and BMI might have a stronger correlation with PE risk in the Chinese population compared to others^1,3^. These differences in PE risk factors among different countries might be attributed to variations in the distribution of these factors in different populations or in the proportion of PE severity. Furthermore, maternal factors, particularly high BMI and elevated blood pressure, could affect cfDNA levels. For example, in pregnant women with higher BMI, the fetal fraction tends to be lower, which could affect the accuracy of cfDNA fragmentomics-based models. Given the existence of these differences, we believed that biomarkers might also differ across populations, collectively influencing the risk of PE occurrence. Nonetheless, our findings still provide valuable insights for clinical practice, particularly in the Chinese population. In addition, an intriguing area for further exploration involves refining algorithms to better accommodate heterogeneous demographic, clinical, and biomarker factors.

The abundance of cfDNA fragments derived from TSS regions in plasma is widely accepted to correlate with gene expression levels in cells due to nucleosome protection^9,10^. This correlation was observed in our study as well. For instance, cfDNA fragment levels at TSS regions of genes such as *AFP* and *CRY1* were lower in pregnant women with PE, and those at the TSS regions of *AKT1*, *EZH2*, *RHOBTB1*, *CYP19A1*, *ERVFRD-1* and *ATF3* genes were elevated, in contrast to the reported expression levels of these genes in placentas or maternal serum^39–46^. In addition to fragment abundance, we also examined cfDNA fragment length diversity, quantified using the Gini coefficient at TSS regions. This metric might also reflect chromatin accessibility and cleavage dynamics that differ between active and inactive genomic regions. Given that PE is associated with dysregulated gene expression and abnormal cell death in placental or blood cells, the Gini coefficient might also capture these underlying biological changes. Indeed, TSS regions of *LRP1B* and *TFDP2* genes exhibited more homogeneous fragment sizes in pregnant women with PE, consistent with their expression in placentas^47,48^. When combined with fragment coverage and other fragmentomic features, it might offer additional predictive value for PE detection. These findings highlight the multifaceted nature of cfDNA fragmentomics and the importance of integrating both abundance- and length diversity-based metrics to gain deeper insights into the pathophysiology of PE. Interestingly, we also found that cfDNA fragment signals at TSS regions of certain genes, such as *SERPINB5* and *A2M*, were altered in PE pregnancies but were not fully explained by their expression levels in preeclamptic placentas^49,50^. We hypothesized that the relationship between cfDNA coverage at a TSS region in plasma and gene expression in cells might be disrupted when other conditions exist, such as transcription factor (TF) occupancy and inappropriate cell death. TSS regions of highly expressed genes in cells might be bound by TFs or other factors rather than nucleosomes, potentially increasing the abundance and decreasing the diversity of short cfDNA fragments in plasma^9^. The concentration of TF binding is known to impact gene expression^51^. In addition, the observed increase in placental autophagy and apoptosis rates in pregnant women with PE could lead to more cell death, consequently increasing the abundance of cfDNA fragments in plasma, including fragments derived from TSS regions of tissue-specific genes^52,53^. Thus, the changes of cfDNA fragment levels and sizes at TSS regions in plasma reflect a combined result of multiple biological mechanisms, including nucleosome and TF occupancies, cell death and degradation. The balance between these biological processes under physiological and pathological conditions requires further exploration in future studies.

Despite its strengths, our study has some limitations. First, as a retrospective study, the samples in our research might not fully represent the overall population. In particular, the control group was defined to exclude not only hypertensive disorders but also other pregnancy complications and maternal comorbidities, and all participants delivered at term, resulting in a cleaner cohort than might be expected in real-world settings. This approach was chosen to minimize potential confounding factors in this exploratory phase, but it likely limits the generalizability of our findings to broader clinical populations. However, by collecting plasma samples from four hospitals and using two completely independent test sets, we enhanced the external validity of the model. Second, to improve the interpretability of cfDNA fragmentomic features, we focused on the features used for PE prediction on their corresponding genes obtained from databases associated with PE. While this might exclude features that contribute to model performance, removing this step resulted in poorer model performance. We believe that these unstable features caused by some interfering factors might not be truly related to the occurrence and development of PE. Further investigations are needed to assess the functionality and authenticity of cfDNA fragmentomic features corresponding to genes that have not yet been reported to play a role in PE. Moreover, our analysis focused on a more limited fragmentomic metric compared to the PFE method^11^. However, we have intended to explore additional types of cfDNA fragmentomics, including genetic information. Overall, we believe the role of cfDNA fragmentomics might have been underestimated in this study. Third, while our analyses demonstrated that fragmentomic metrics, such as TSS coverage, TSS score, and Gini coefficient, showed statistically significant differences between gene expression categories at a read depth of ~10$\times$, we acknowledge that this depth does not necessarily represent the optimal threshold for cfDNA fragmentomics analyses and predictive model development. Instead, the ~10 $\times$ read depth might represent a reasonable depth required for relatively stable estimation of cfDNA fragmentomic metrics, at which biologically informative signals could be extracted and used for risk prediction. Moreover, our study relied on an approximately 10 $\times$ paired-end sequencing depth, which is higher than what is routinely performed in standard clinical NIPT workflows, representing a limitation compared with the study by Adil et al. Further studies leveraging a broader spectrum of sequencing depths and alternative modeling strategies might uncover more optimal analytical frameworks. Finally, although we adopted several strategies to reduce model overfitting, such as stratified cross-validation during parameter tuning and setting larger values for the ‘min_samples_leaf’ parameter in the random forest classifier, we acknowledge that a degree of overfitting might still exist. Such overfitting is a known limitation when using relatively small or highly curated datasets. Future prospective studies in larger and more diverse populations are needed to further assess the generalizability of our predictive models.

Collectively, our findings suggested the potential of combining cfDNA fragmentomics with maternal factors to enhance the prediction of PE.

## Method

### Study overview and participants

Study overview. All samples analyzed in this study were retrospectively collected from 1058 pregnant women aged 20–45 years who were enrolled at four hospitals in China from Jan 1, 2019, to Jun 30, 2022, including the Obstetrics & Gynecology Hospital of Fudan University, Zhuhai Center for Maternal and Child Health Care, Jiangmen Central Hospital, and Inner Mongolia Maternity and Child Health Care Hospital. This study was approved by the Ethics Committee of Beijing Genomics Institute (BGI) (BGI-IRB 22134-T1), as well as the respective Ethics Committees of the participating hospitals: Obstetrics & Gynecology Hospital of Fudan University (2023-56), Zhuhai Center for Maternal and Child Health Care ([2022]01), Jiangmen Central Hospital ([2022]02), and Inner Mongolia Maternity and Child Health Care Hospital ([2023]090). This study is compliant with the Guidance of the Ministry of Science and Technology (MOST) of China for the Review and Approval of Human Genetic Resources. All participants provided written informed consent.

To develop models based on cfDNA fragmentomics for predicting PE, we profiled three types of cfDNA fragmentomics from a plasma sample of a pregnant woman by deep WGS ( ~ 50$\times$) to learn the relationships between these fragmentomics and gene expression levels in blood and placental tissues. Fragmentomic features were established using cfDNA WGS, and their stabilities in relation to gene expression levels in blood and placental tissues were assessed at different sequencing depths (Supplementary Data 1). After determining that a sequencing depth of > 10$\times$ was adequate for analyzing cfDNA fragmentomics, cfDNA profiling data from 1058 participants were generated for this study. These participants include 507 pregnant women who developed early-onset PE (*n* = 138) and late-onset PE (*n* = 369), and 551 healthy controls. To ensure data independence, samples from different hospitals were divided into separate datasets. The training set comprised samples from Obstetrics and Gynecology Hospital, Fudan University, including 56 pregnant women with early-onset PE, 84 pregnant women with late-onset PE, and 146 healthy individuals. The validation set included samples from Zhuhai Center for Maternal and Child Health Care, with 23 pregnant women with early-onset PE, 59 pregnant women with late-onset PE, and 84 healthy individuals. Two independent test sets used exclusively for the final validation of model performance were created using samples from Jiangmen Central Hospital and Inner Mongolia Maternity and Child Health Care Hospital, respectively. The test sets included 23 and 36 pregnant women with early-onset PE, 135 and 91 pregnant women with late-onset PE, and 197 and 124 healthy individuals, respectively (Fig. 2).

Pregnant women with PE. To develop and evaluate models for predicting PE, cfDNA WGS (20–40$\times$) was performed on plasma samples from 507 pregnant women with PE who did not take aspirin during pregnancy. Plasma samples were collected at 12–26 weeks’ gestation. PE was defined as maternal systolic blood pressure ≥ 140 mmHg and/or diastolic blood pressure ≥ 90 mmHg after 20 weeks of gestation, accompanied by one or more of the following new-onset conditions: 1) proteinuria level ≥ 0.3 g/24 h; 2) neurologic complications; 3) platelet count <150,000/µl; 4) kidney injury (creatinine ≥ 90 µmol/liter or ≥ 1 mg/dl); 5) pulmonary edema; 6) liver injury (ALT or AST > 40 IU/liter)^54^. Pregnant women who developed PE before 34 weeks of gestation were classified as early-onset PE, whereas those with onset at or after 34 weeks of gestation were classified as having late-onset PE^3,6^.

Healthy participants. For healthy controls, we randomly selected 146, 84, 197 and 124 plasma samples from pregnant women at four hospitals whose gestational age matched that of pregnant women with early-onset and late-onset PE, respectively (Table 1, Supplementary Tables 1–3 and Supplementary Fig. 2). These pregnant women had uncomplicated pregnancies, delivered at term, and were without severe cardiac, hepatic and renal insufficiency, malignant neoplastic diseases, and chromosomal abnormalities.

### Sequencing data collection

#### Whole-genome sequencing of cfDNA

Five milliliters of maternal peripheral blood were collected into Streck Cell Free DNA BCT^®^ blood collection tubes (Streck, La Vista, NE, USA). For each sample, 200 microliters of maternal plasma were processed for cfDNA extraction using the MGIEasy Circulating DNA Isolation Kit (MGI, Shenzhen, China). The extracted cfDNA was then used to construct a library using the MGIEasy Cell-free DNA Library Prep Kit (MGI, Shenzhen, China). Briefly, the extracted cfDNA underwent end-repair, addition of an “A” tail, and ligation with adapters. The ligated products were subsequently purified and amplified through 15 cycles of PCR. The PCR products were purified, quantitated with a dsDNA Fluorescence Assay Kit (Invitrogen, United States), heat-denatured, and incubated at 37 °C to create ssDNA circles. These circles were subjected to rolling circle amplification to generate DNA nanoballs. Finally, libraries were sequenced on the DNBSEQ-T7 platform (MGI Tech Co., Ltd.), using multiplex sequencing, producing paired-end 100 bp (PE100) reads at a depth of 20–40 $\times$.

#### Transcript expression data in the placenta and whole-blood

Transcript expression levels in the placenta were obtained from RNA-seq data downloaded from The Human Protein Atlas^55^. Genes were divided into eight groups based on their transcripts per million (TPM) values in the placenta (TPM = 0; 0 < TPM ≤ 10; 10 < TPM ≤ 20; 20 < TPM ≤ 30; 30 < TPM ≤ 40; 40 < TPM ≤ 50; 50 < TPM ≤ 60; TPM > 60). Whole-blood gene expression levels were retrieved from the Human 133 A/GNF1H Gene Atlas Database (GSE1133)^56^ and similarly divided into eight groups (expression = 0; 0 <expression ≤ 50; 50 <expression ≤ 100; 100 <expression ≤ 150; 150 <expression ≤ 200; 200 <expression ≤ 250; 250 <expression ≤ 300; and expression > 300) (Supplementary Data 2). Genes with an expression level of 0 in whole blood corresponded to those previously reported as not expressed in any tissues^10^.

## Data analysis methods

### Mapping and alignment quality control

Adapters were trimmed from the 3’ end of the sequencing reads using SOAPnuke filter (2.1.7)^57^. Then, clean reads were aligned to the GRCh38 human reference (GCA_000001405.15) using BWA (0.7.17-r1188)^58^. To ensure reliable downstream analyses, non-unique reads, duplicate reads, and reads with supplementary or secondary alignments were excluded using SAMTOOLS (1.15.1)^59^. Only reads with specific Sequence Alignment/Map (SAM) flags (81, 83, 97, 99, 145, 147, 161, and 163) were retained for cfDNA fragmentomics analysis, which correspond to properly paired, uniquely mapped, and high-quality read pairs in a paired-end sequencing dataset.

### CfDNA fragmentomics analysis

We analyzed three types of cfDNA fragmentomics at transcription start site (TSS) regions, including TSS coverage, TSS score, and Gini coefficient (Fig. 1a–c). The TSS region was defined as a 2-kilobase (kb) window flanking the TSS (-1000 bp to +1000 bp) for each transcript downloaded from NCBI (GRCh38). Transcripts sharing the same transcription start site (TSS) were merged to eliminate redundancy, ensuring that each TSS region was represented only once in the analysis. After determining the adequate sequencing depth required to analyze these fragmentomics as described above, we computed the TSS coverage, TSS score, and Gini coefficient for each transcript in the 1058 cfDNA WGS samples.

TSS coverage. The number of reads mapped to each 2 kb TSS region was calculated using BEDtools (ver. 2.29.2)^60^ (1).

1 $${{{\rm{TSS}}}}_{{{\rm{i}}}}\,{{\rm{coverage}}}=\frac{{{\rm{the}}}\; {{\rm{number}}}\; {{\rm{of}}}{{{\rm{mapped}}}\; {{\rm{reads}}}\; {{\rm{on}}}\; {{\rm{the}}}\; {{\rm{TSS}}}}_{{{\rm{i}}}}\times {10}^{9}}{{{\rm{the}}}\; {{\rm{total}}}\; {{\rm{number}}}\; {{\rm{of}}}\; {{\rm{mapped}}}\; {{\rm{reads}}}\times 2000}$$

TSS score. To evaluate the distribution of mapped reads within each 2 kb TSS region, we divided each region into 20 bins of 100 bp size. The TSS score for each TSS region was computed as the ratio of the mean read depth of the 6 bins closest to the TSS to the mean read depth of the 10 bins furthest from the TSS.

2 $${{{\rm{TSS}}}}_{{{\rm{i}}}}{{\rm{score}}}=\left(\frac{\sum ({{\rm{read}}}\; {{\rm{depth}}}\; {{\rm{of}}}\; {{\rm{midBin}}})/6}{\sum ({{\rm{read}}}\; {{\rm{depth}}}\; {{\rm{of}}}\; {{\rm{sideBin}}})/10}\right)\\ \times \frac{{10}^{9}}{{{\rm{the}}}\; {{\rm{total}}}\; {{\rm{number}}}\; {{\rm{of}}}\; {{\rm{mapped}}}\; {{\rm{reads}}}\times 100}$$

Gini coefficient. We separately calculated the number of 100 bp–300 bp cfDNA fragments mapped to each 2 kb TSS region. The Gini coefficient, a measure of the diversity of cfDNA fragment sizes, was computed for each 2 kb TSS region using the DescTools package in R (4.1.3) with 201 cfDNA fragment numbers (ranging from 100 bp to 300 bp).

The sequencing depth was adjusted for all three types of cfDNA fragmentomics using linear models. For each batch, we also computed the multiple of the median (MoM) values of TSS coverage, TSS score, and Gini coefficient for each TSS region based on the median value of control samples.

#### Spearman correlation analysis

Genes were divided into 500 equal parts based on their expression levels in the placenta or whole blood. For each partition, we calculated the mean gene expression in the placenta or whole blood and the corresponding mean cfDNA fragmentomic metric. The Spearman correlation coefficient between the two variables was calculated across all 500 partitions.

#### Feature selection for each cfDNA fragmentomic

We combined the training and validation datasets for feature selection and employed three methods to select features for each cfDNA fragmentomic (Fig. 2a).

Differential analysis. Stable differential features for each cfDNA fragmentomic (TSS coverage, TSS score, and Gini coefficient) between PE samples and healthy controls were identified by performing 1000 iterations of the Wilcoxon rank sum test on randomly sampled subsets (90% of samples per iteration) (Fig. 3a). The Benjamini-Hochberg (BH) procedure was applied to adjust the *p*-values. For each feature, we calculated the 95% confidence interval (CI) for adjusted *p*-values of 1000 differential analyses. CfDNA fragmentomic features were included in subsequent analyses if they met the following criteria: 1) the adjusted *p*-value (the upper limit of 95% CI) < 0.05; 2) the direction of change between PE samples and healthy controls was consistent in all 1000 iterations. Then, to improve the interpretability of features for each cfDNA fragmentomic, we included differential features in the following analyses if their corresponding genes had been reported in GeneCards^25^, cPE^23^, or dbPEC^24^.

Random forest algorithm. Feature importance was estimated using the ‘BorutaPy’ package for the features, which were selected as described above. The maximum number of iterations was set to 1000. Feature rankings were assigned in descending order and normalized to the maximum value, such that higher output values indicated greater importance. Then, the top 20 features for each cfDNA fragmentomic were selected for the subsequent analysis, respectively.

Regression model. We used the ‘regsubsets’ function from the ‘leaps’ package in R to identify the best subset of the top 20 features for each cfDNA fragmentomic. This process was embedded within a 10-fold cross-validation framework to assess model generalization. In each cross-validation iteration, one fold was designated as the test set, while the remaining nine folds served as the training set. Within each training set, ‘regsubsets’ was applied to evaluate different feature subsets and identify the best-performing combination. The mean squared error (MSE) was then computed for the selected feature subset on the corresponding test set. To assess overall model performance, the MSE values from all 10 folds were averaged. This approach allowed us to determine the optimal feature combination, which minimized the average MSE and enhanced the model’s generalization ability and predictive accuracy.

Interpretation of cfDNA fragmentomics. The Shapley Additive Explanations (SHAP) method from the ‘shapviz’ package in R was used to explain the relative contributions of selected features in random forest models. Features were ordered according to the mean absolute SHAP values across the 10-fold cross-validation.

### Clinical risk factors analysis

For clinical characteristics, the maternal age, height, weight and blood pressure measured at the same gestational age as maternal plasma sampling, as well as parity, gravidity, past medical history and method of conception were collected. The past medical history included the history of chronic hypertension, PE, systemic lupus erythematosus (SLE) and antiphospholipid syndrome.

Body mass index (BMI). The formula for BMI is the weight in kilograms divided by the square of the height in meters.

Mean arterial pressure (MAP)^61^. MAP in females was calculated using the following formula (3). DBP and SBP are abbreviations for diastolic and systolic blood pressure.

3 $${{\rm{MPA}}}={{\rm{DBP}}}+({{\rm{SBP}}}-{{\rm{DBP}}})\times \frac{(27.07+0.181\times {{\rm{DBP}}}+2.303)}{100}$$

Then, MoM values were calculated for maternal age, BMI and MAP, as described above for cfDNA fragmentomic features.

### Pathway enrichment analysis

Pathway enrichment analyses and DisGeNet gene-disease associations for genes associated with differential TSS coverage, scores, or Gini coefficients were performed using Metascape^62^.

### Statistical analysis

The Fisher’s exact test (two-sided) was used to compare categorical variables between PE samples and healthy controls, such as gravidity, parity, past medical history and method of conception. The Wilcoxon rank sum test (two-sided) was utilized to compare continuous variables between two groups, such as maternal age, BMI, MAP and cfDNA fragmentomics. The Kruskal-Wallis test was used to determine whether there was a statistically significant difference among three or more groups. The DeLong’s test (two-sided) was used to compare the area under the curves (AUCs) of two models. The Benjamini-Hochberg (BH) procedure was applied for multiple comparison corrections, with adjusted *p*-value < 0.05 considered statistically significant.

### Models construction and evaluation

Random forest models were developed using cfDNA fragmentomics, with or without maternal risk factors, to predict early-onset or late-onset PE on the training set. Model performance was evaluated on the validation set and test sets, respectively. The 95% confidence intervals of AUCs, sensitivities, specificities, accuracies, negative predictive values (NPVs) and positive predictive values (PPVs) were calculated using 1000 bootstrap samples for the training set, validation set and test sets. Each bootstrap sample had the same size as the original dataset. Receiver operating characteristic (ROC) curves were plotted, and AUC, sensitivity, specificity, accuracy, NPV, and PPV values were calculated by custom scripts in Python.

### Reporting summary

Further information on research design is available in the Nature Portfolio Reporting Summary linked to this article.

## Supplementary information

Supplementary Information

Peer Review File

Description of Additional Supplementary Files

Supplementary Data 1

Supplementary Data 2

Supplementary Data 3

Supplementary Data 4

Supplementary Data 5

Supplementary Data 6

Supplementary Data 7

Supplementary Data 8

Supplementary Data 9

Supplementary Data 10

Supplementary Data 11

Supplementary Data 12

Supplementary Data 13

Supplementary Data 14

Supplementary Data 15

Supplementary Data 16

Supplementary Data 17

Supplementary Data 18

Supplementary Data 19

Supplementary Data 20

Supplementary Data 21

Supplementary Data 22

Supplementary Data 23

Reporting Summary

## Source data

Source Data

## Data Availability

The BED files for cfDNA fragmentomics analysis in pregnant individuals have been deposited in the OMIX, which is part of the Genome Sequence Archive (GSA) infrastructure for human omics data, under accession number OMIX012350. The dataset is available at: https://ngdc.cncb.ac.cn/omix/release/OMIX012350. In accordance with the current regulations of the People’s Republic of China on the administration of human genetic resources (https://flk.npc.gov.cn/index.html), the raw genomic sequencing data contain potentially identifiable genetic information and are therefore available under controlled access. Requests for access should be directed to the corresponding authors and must include a brief description of the intended research purpose and plan. Requests will be reviewed within two weeks. Access will be granted upon approval and completion of a formal data access agreement, which restricts use to non-commercial research purposes and prohibits redistribution to third parties without prior written consent. The key supporting data generated in this study are provided in the article, Supplementary Information and Source data files. Source data are provided with this paper.

## Code availability

All codes and figure source data in this study have been deposited in Code Ocean and are publicly available at 10.24433/CO.7028212.v1.

## References

[1] Yang, Y. et al. Preeclampsia prevalence, risk factors, and pregnancy outcomes in Sweden and China. *JAMA Netw. Open* **4**, e218401 (2021). 33970258 10.1001/jamanetworkopen.2021.8401 PMC8111481

[2] Fasanya, H. O., Hsiao, C. J., Armstrong-Sylvester, K. R. & Beal, S. G. A critical review on the use of race in understanding racial disparities in preeclampsia. *J. Appl Lab Med* **6**, 247–256 (2021). 33227139 10.1093/jalm/jfaa149 PMC8516080

[3] Shi, P. et al. Differences in epidemiology of patients with preeclampsia between China and the US (Review). *Exp. Ther. Med* **22**, 1012 (2021). 34345294 10.3892/etm.2021.10435 PMC8311229

[4] Hoffman, M. K. et al. Low-dose aspirin for the prevention of preterm delivery in nulliparous women with a singleton pregnancy (ASPIRIN): a randomised, double-blind, placebo-controlled trial. *Lancet* **395**, 285–293 (2020). 31982074 10.1016/S0140-6736(19)32973-3 PMC7168353

[5] Rolnik, D. L. et al. Aspirin versus placebo in pregnancies at high risk for preterm preeclampsia. *N. Engl. J. Med* **377**, 613–622 (2017). 28657417 10.1056/NEJMoa1704559

[6] Lisonkova, S. & Joseph, K. S. Incidence of preeclampsia: risk factors and outcomes associated with early- versus late-onset disease. *Am. J. Obstet. Gynecol.* **209**, 544 e541–544 e512 (2013). 10.1016/j.ajog.2013.08.019 23973398

[7] Mandel, P. & Metais, P. [Nuclear acids in human blood plasma]. *C. R. Seances Soc. Biol. Fil.* **142**, 241–243 (1948). 18875018

[8] Ulz, P. et al. Inferring expressed genes by whole-genome sequencing of plasma DNA. *Nat. Genet* **48**, 1273–1278 (2016). 27571261 10.1038/ng.3648

[9] Snyder, M. W., Kircher, M., Hill, A. J., Daza, R. M. & Shendure, J. Cell-free DNA comprises an in vivo nucleosome footprint that informs its tissues-of-origin. *Cell* **164**, 57–68 (2016). 26771485 10.1016/j.cell.2015.11.050 PMC4715266

[10] Guo, Z. et al. Whole-genome promoter profiling of plasma DNA exhibits diagnostic value for placenta-origin pregnancy complications. *Adv. Sci. (Weinh.)* **7**, 1901819 (2020). 32274292 10.1002/advs.201901819 PMC7141029

[11] Esfahani, M. S. et al. Inferring gene expression from cell-free DNA fragmentation profiles. *Nat. Biotechnol.* **40**, 585–597 (2022). 35361996 10.1038/s41587-022-01222-4 PMC9337986

[12] Mathios, D. et al. Detection and characterization of lung cancer using cell-free DNA fragmentomes. *Nat. Commun.* **12**, 5060 (2021). 34417454 10.1038/s41467-021-24994-w PMC8379179

[13] Ulz, P. et al. Inference of transcription factor binding from cell-free DNA enables tumor subtype prediction and early detection. *Nat. Commun.* **10**, 4666 (2019). 31604930 10.1038/s41467-019-12714-4 PMC6789008

[14] Sun, K. et al. Orientation-aware plasma cell-free DNA fragmentation analysis in open chromatin regions informs tissue of origin. *Genome Res.* **29**, 418–427 (2019). 30808726 10.1101/gr.242719.118 PMC6396422

[15] Chan, K. C. et al. Second-generation noninvasive fetal genome analysis reveals de novo mutations, single-base parental inheritance, and preferred DNA ends. *Proc. Natl. Acad. Sci. USA* **113**, E8159–E8168 (2016). 27799561 10.1073/pnas.1615800113 PMC5167168

[16] Jiang, P. et al. Plasma DNA end-motif profiling as a fragmentomic marker in cancer, pregnancy, and transplantation. *Cancer Discov.* **10**, 664–673 (2020). 32111602 10.1158/2159-8290.CD-19-0622

[17] Jiang, P. et al. Detection and characterization of jagged ends of double-stranded DNA in plasma. *Genome Res.* **30**, 1144–1153 (2020). 32801148 10.1101/gr.261396.120 PMC7462074

[18] Xu, C. et al. Non-invasive prediction of fetal growth restriction by whole-genome promoter profiling of maternal plasma DNA: a nested case-control study. *BJOG* **128**, 458–466 (2021). 32364311 10.1111/1471-0528.16292 PMC7818264

[19] Chen, X. et al. Transcriptional start site coverage analysis in plasma cell-free DNA reveals disease severity and tissue specificity of COVID-19 patients. *Front Genet* **12**, 663098 (2021). 34122515 10.3389/fgene.2021.663098 PMC8194351

[20] Kontovazainitis, C. G., Gialamprinou, D., Theodoridis, T. & Mitsiakos, G. Hemostasis in pre-eclamptic women and their offspring: current knowledge and hemostasis assessment with viscoelastic tests. *Diagnostics* **14**, 347 (2024). 38337863 10.3390/diagnostics14030347 PMC10855316

[21] Grzeszczak, K., Lanocha-Arendarczyk, N., Malinowski, W., Zietek, P. & Kosik-Bogacka, D. Oxidative stress in pregnancy. *Biomolecules* **13**, 1768 (2023). 38136639 10.3390/biom13121768 PMC10741771

[22] Escudero, C. et al. Increased placental angiogenesis in late and early onset pre-eclampsia is associated with differential activation of vascular endothelial growth factor receptor 2. *Placenta* **35**, 207–215 (2014). 24508097 10.1016/j.placenta.2014.01.007

[23] Li, X. et al. Identifying preeclampsia-associated genes using a control theory method. *Brief. Funct. Genomics* **21**, 296–309 (2022). 35484822 10.1093/bfgp/elac006 PMC9328024

[24] Uzun, A., Triche, E. W., Schuster, J., Dewan, A. T. & Padbury, J. F. dbPEC: a comprehensive literature-based database for preeclampsia related genes and phenotypes. *Database* **2016**, baw006 (2016). 26946289 10.1093/database/baw006 PMC4779341

[25] Stelzer, G. et al. The genecards suite: from gene data mining to disease genome sequence analyses. *Curr. Protoc. Bioinforma.* **54**, 31–31 (2016). 10.1002/cpbi.5 27322403

[26] De Borre, M. et al. Cell-free DNA methylome analysis for early preeclampsia prediction. *Nat. Med* **29**, 2206–2215 (2023). 37640858 10.1038/s41591-023-02510-5

[27] Wright, D., Wright, A. & Nicolaides, K. H. The competing risk approach for prediction of preeclampsia. *Am. J. Obstet. Gynecol.* **223**, 12–23 e17 (2020). 31733203 10.1016/j.ajog.2019.11.1247

[28] Chappell, L. C., Cluver, C. A., Kingdom, J. & Tong, S. Pre-eclampsia. *Lancet* **398**, 341–354 (2021). 34051884 10.1016/S0140-6736(20)32335-7

[29] Poon, L. C. et al. The International Federation of Gynecology and Obstetrics (FIGO) initiative on pre-eclampsia: a pragmatic guide for first-trimester screening and prevention. *Int J. Gynaecol. Obstet.* **145**, 1–33 (2019). 31111484 10.1002/ijgo.12802 PMC6944283

[30] Chaemsaithong, P., Sahota, D. S. & Poon, L. C. First-trimester preeclampsia screening and prediction. *Am. J. Obstet. Gynecol.* **226**, S1071–S1097 e1072 (2022). 32682859 10.1016/j.ajog.2020.07.020

[31] O’Gorman, N. et al. Competing risks model in screening for preeclampsia by maternal factors and biomarkers at 11–13 weeks of gestation. *Am. J. Obstet. Gynecol.* **214**, 103 e101–103 e112 (2016). 10.1016/j.ajog.2015.08.034 26297382

[32] Chaemsaithong, P. et al. Prospective evaluation of screening performance of first-trimester prediction models for preterm preeclampsia in an Asian population. *Am. J. Obstet. Gynecol.* **221**, 650 e651–650 e616 (2019). 10.1016/j.ajog.2019.09.041 31589866

[33] Hu, J. et al. Prospective evaluation of first-trimester screening strategy for preterm pre-eclampsia and its clinical applicability in China. *Ultrasound Obstet. Gynecol.* **58**, 529–539 (2021). 33817865 10.1002/uog.23645

[34] Dungan, J. S. et al. Noninvasive prenatal screening (NIPS) for fetal chromosome abnormalities in a general-risk population: an evidence-based clinical guideline of the American College of Medical Genetics and Genomics (ACMG). *Genet. Med.* **25**, 100336 (2023). 36524989 10.1016/j.gim.2022.11.004

[35] ACOG Committee Opinion No. 743: low-dose aspirin use during pregnancy. *Obstet. Gynecol.* **132**, e44-e52 (2018). 10.1097/AOG.0000000000002708 29939940

[36] Adil, M. et al. Preeclampsia risk prediction from prenatal cell-free DNA screening. *Nat. Med* **31**, 1312–1318 (2025). 39939524 10.1038/s41591-025-03509-w PMC12003088

[37] Yu, Y. et al. Non-invasive prediction of preeclampsia using the maternal plasma cell-free DNA profile and clinical risk factors. *Front. Med.* **11**, 1254467 (2024). 10.3389/fmed.2024.1254467 PMC11061442 38695016

[38] Moufarrej, M. N. et al. Early prediction of preeclampsia in pregnancy with cell-free RNA. *Nature* **602**, 689–694 (2022). 35140405 10.1038/s41586-022-04410-z PMC8971130

[39] Waller, D. K., Lustig, L. S., Cunningham, G. C., Feuchtbaum, L. B. & Hook, E. B. The association between maternal serum alpha-fetoprotein and preterm birth, small for gestational age infants, preeclampsia, and placental complications. *Obstet. Gynecol.* **88**, 816–822 (1996). 8885920 10.1016/0029-7844(96)00310-9

[40] Cudmore, M. J. et al. Loss of Akt activity increases circulating soluble endoglin release in preeclampsia: identification of inter-dependency between Akt-1 and heme oxygenase-1. *Eur. Heart J.* **33**, 1150–1158 (2012). 21411816 10.1093/eurheartj/ehr065 PMC3341632

[41] Zhou, G. et al. Co-alterations of circadian clock gene transcripts in human placenta in preeclampsia. *Sci. Rep.* **12**, 17856 (2022). 36284122 10.1038/s41598-022-22507-3 PMC9596722

[42] Qian, X. & Zhang, Y. EZH2 enhances proliferation and migration of trophoblast cell lines by blocking GADD45A-mediated p38/MAPK signaling pathway. *Bioengineered* **13**, 12583–12597 (2022). 35609316 10.1080/21655979.2022.2074620 PMC9275956

[43] Zhang, Y., Jiang, G. & Zhang, C. Downregulation of Cullin 3 Ligase Signaling Pathways Contributes to Hypertension in Preeclampsia. *Front. Cardiovasc. Med.* **8**, 654254 (2021). 33928137 10.3389/fcvm.2021.654254 PMC8076533

[44] Kaitu’u-Lino, T. J. et al. Activating transcription factor 3 is reduced in preeclamptic placentas and negatively regulates sFlt-1 (soluble fms-like tyrosine kinase 1), soluble endoglin, and proinflammatory cytokines in the placenta. *Hypertension* **70**, 1014–1024 (2017). 28947613 10.1161/HYPERTENSIONAHA.117.09548

[45] Zhu, D., Huang, J., Gu, X., Li, L. & Han, J. Downregulation of aromatase plays a dual role in preeclampsia. *Mol. Hum. Reprod.* **27**, gaab013 (2021). 33624796 10.1093/molehr/gaab013

[46] Vargas, A. et al. Reduced expression of both syncytin 1 and syncytin 2 correlates with the severity of preeclampsia. *Reprod. Sci.* **18**, 1085–1091 (2011). 21493955 10.1177/1933719111404608

[47] Chen, Z. et al. Placental whole transcriptome expression profile in patients with early-onset, late-onset preeclampsia and gestational diabetes mellitus. *Sci. Rep.* **15**, 19476 (2025). 40461719 10.1038/s41598-025-04836-1 PMC12134284

[48] Vaiman, D., Calicchio, R. & Miralles, F. Landscape of transcriptional deregulations in the preeclamptic placenta. *PLoS ONE* **8**, e65498 (2013). 23785430 10.1371/journal.pone.0065498 PMC3681798

[49] Shi, X. et al. Decitabine improves the clinical manifestations of rats with l-NAME-induced pre-eclampsia: a potential approach to studying pre-eclampsia. *Hypertens. Pregnancy* **34**, 464–473 (2015). 26389732 10.3109/10641955.2015.1074245

[50] Wang, J. et al. Alpha-2-macroglobulin is involved in the occurrence of early-onset pre-eclampsia via its negative impact on uterine spiral artery remodeling and placental angiogenesis. *BMC Med.* **21**, 90 (2023). 36894970 10.1186/s12916-023-02807-9 PMC9999529

[51] Givre, A., Colman-Lerner, A. & Ponce Dawson, S. Modulation of transcription factor dynamics allows versatile information transmission. *Sci. Rep.* **13**, 2652 (2023). 36788258 10.1038/s41598-023-29539-3 PMC9929046

[52] Kasture, V., Sahay, A. & Joshi, S. Cell death mechanisms and their roles in pregnancy-related disorders. *Adv. Protein Chem. Struct. Biol.* **126**, 195–225 (2021). 34090615 10.1016/bs.apcsb.2021.01.006

[53] Lokeswara, A. W., Hiksas, R., Irwinda, R. & Wibowo, N. Preeclampsia: from cellular wellness to inappropriate cell death, and the roles of nutrition. *Front Cell Dev. Biol.* **9**, 726513 (2021). 34805141 10.3389/fcell.2021.726513 PMC8602860

[54] Magee, L. A., Nicolaides, K. H. & von Dadelszen, P. Preeclampsia. *N. Engl. J. Med.* **386**, 1817–1832 (2022). 35544388 10.1056/NEJMra2109523

[55] Sjostedt, E. et al. An atlas of the protein-coding genes in the human, pig, and mouse brain. *Science* **367**, eaay5947 (2020). 32139519 10.1126/science.aay5947

[56] Su, A. I. et al. A gene atlas of the mouse and human protein-encoding transcriptomes. *Proc. Natl. Acad. Sci. USA* **101**, 6062–6067 (2004). 15075390 10.1073/pnas.0400782101 PMC395923

[57] Chen, Y. et al. SOAPnuke: a MapReduce acceleration-supported software for integrated quality control and preprocessing of high-throughput sequencing data. *Gigascience* **7**, 1–6 (2018). 29220494 10.1093/gigascience/gix120 PMC5788068

[58] Li, H. & Durbin, R. Fast and accurate long-read alignment with Burrows-Wheeler transform. *Bioinformatics* **26**, 589–595 (2010). 20080505 10.1093/bioinformatics/btp698 PMC2828108

[59] Li, H. et al. The sequence alignment/map format and SAMtools. *Bioinformatics* **25**, 2078–2079 (2009). 19505943 10.1093/bioinformatics/btp352 PMC2723002

[60] Quinlan, A. R. & Hall, I. M. BEDTools: a flexible suite of utilities for comparing genomic features. *Bioinformatics* **26**, 841–842 (2010). 20110278 10.1093/bioinformatics/btq033 PMC2832824

[61] Grillo, A. et al. Mean arterial pressure estimated by brachial pulse wave analysis and comparison with currently used algorithms. *J. Hypertens.* **38**, 2161–2168 (2020). 32694334 10.1097/HJH.0000000000002564

[62] Zhou, Y. et al. Metascape provides a biologist-oriented resource for the analysis of systems-level datasets. *Nat. Commun.* **10**, 1523 (2019). 30944313 10.1038/s41467-019-09234-6 PMC6447622

