# Supplementary material for: Cell-free DNA fragmentomics for preeclampsia risk assessment
Source: Nat Commun. 2026 May 2;17:5957. doi: 10.1038/s41467-026-72682-4 (PMC13342102; doi:10.1038/s41467-026-72682-4)
Supplement: Supplementary file 1 — Supplementary Information [file 41467_2026_72682_MOESM1_ESM.pdf]

Supplementary information

Cell-free DNA fragmentomics for preeclampsia risk assessment

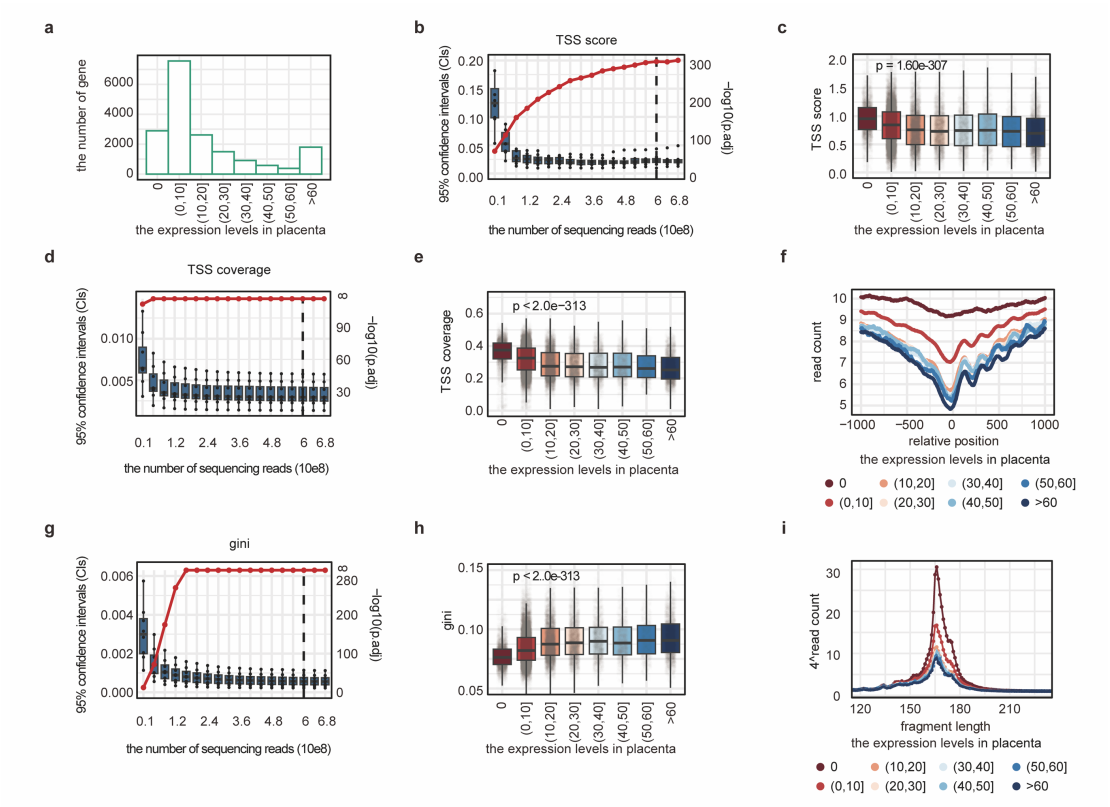

Supplementary Fig. 1|The relationship between cfDNA fragmentomics and gene expression in placentas. **a**, The number of expressed genes across eight groups, categorized by gene expression levels in placentas (TPM=0, >0 and <=10, >10 and <=20, >20 and <=30, >30 and <=40, >40 and <=50, >50 and <=60, >60). The gene expression levels were obtained through RNA sequencing and normalized using Transcripts Per Million (TPM). **b**, Similar to **Fig. 1d,** boxplots depict the 95% confidence interval (CI) of TSS scores within each gene group (left y-axis), while line plots depict the significance of differences in TSS scores among the eight groups using Kruskal-Wallis test (right y-axis). **c**, Boxplots showing TSS scores for different gene groups at 600 million sequencing reads. **d**, Similar to **b**, box and line plots depict the distributions and differences in TSS coverages within each gene group (left y-axis) and among the eight groups of genes (right y-axis), respectively. **e**, Similar to **c**, boxplots depict TSS coverages for different gene groups at 600 million sequencing reads. **f**, Dot and line plots depict the mean number of mapped reads per base at the 2 kb TSS regions of different gene groups. **g**, Similar to **b** and **d**, box and line plots depict the distributions and differences in Gini coefficients within each gene group (left y-axis) and among the eight groups of genes (right y-axis), respectively. **h**, Similar to **c** and **e**, boxplots depict

Gini coefficients for different gene groups at 600 million sequencing reads. **i**, Dot and line plots depict the length distribution of mapping reads at 2 kb TSS regions of different gene groups. The dashed lines in **b**, **d**, and **g** represent the results at 600 million sequencing reads. The Kruskal-Wallis Test was used to compare the significance of differences between three or more groups and adjusted p-values were corrected using the Benjamini-Hochberg (BH) method for multiple comparisons. Box plots (**b-e, g, h**) show the median (centre line, 50 th percentile), with box bounds representing the 25 th (first quartile, Q1) and 75 th (third quartile, Q3) percentiles. Whiskers extend from the minimum to maximum values within Q1-1.5×IQR and Q3+1.5×IQR, where IQR is the interquartile range. Source data are provided as a Source Data file.

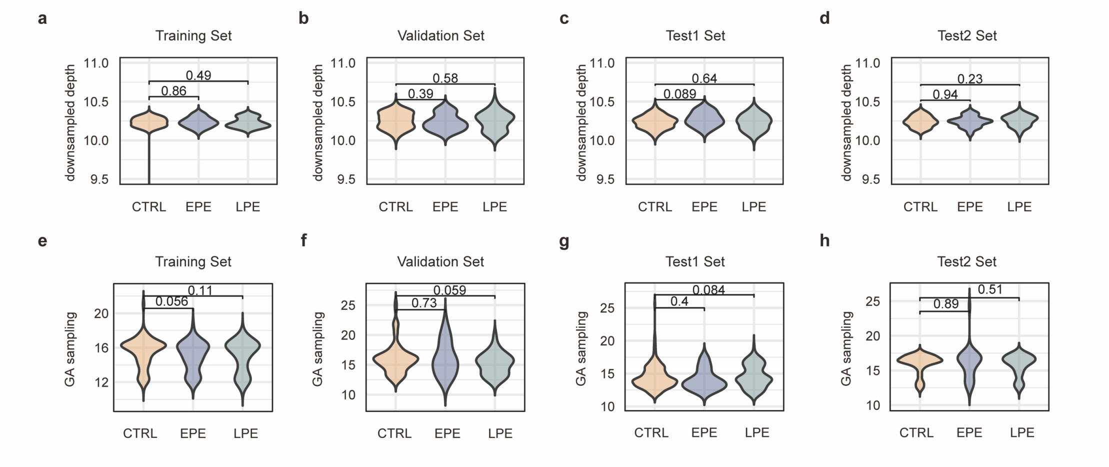

Supplementary Fig. 2|Downsampled depth and gestational age for sampling. The violin plots show downsampled depth of whole genome sequencing of plasma cfDNA (**a-d**) and the gestational age at the time of plasma sampling (**e-h**) from pregnant women at four hospitals, respectively. Violin width reflects data density, with the vertical range representing all observed values (minimum to maximum). CTRL: control; EPE: early-onset preeclampsia; LPE: late-onset preeclampsia. Source data are provided as a Source Data file.

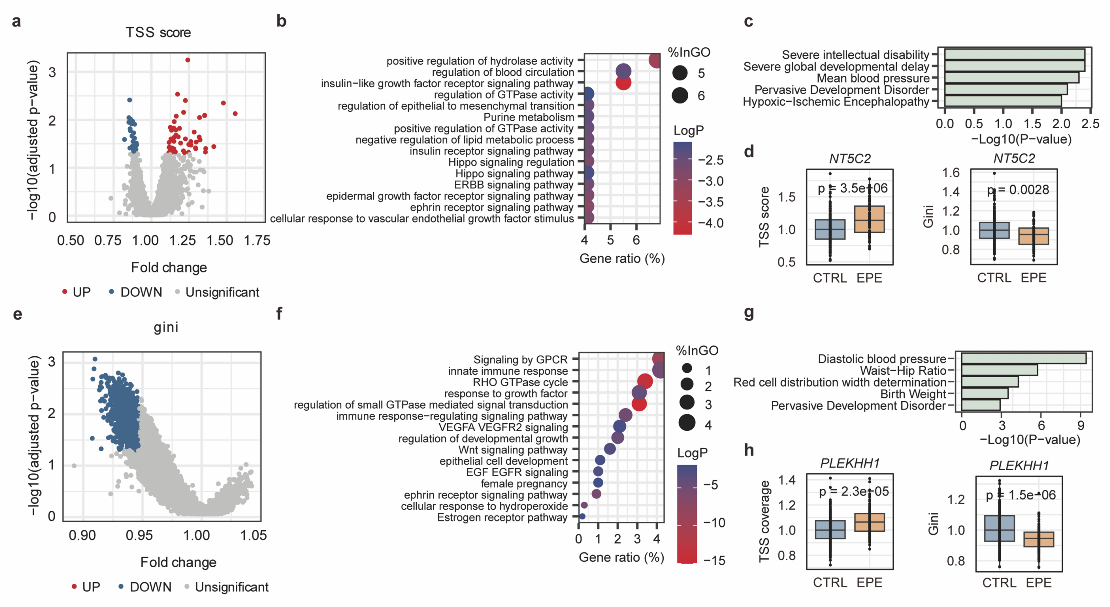

Supplementary Fig. 3|Differential cfDNA fragmentomics analysis between early-onset PE and healthy controls. a, e, Similar to **Fig. 3b**, differential TSS scores and Gini coefficients between early-onset PE samples and controls. TSS regions with significant higher and lower TSS scores (**a**) or Gini coefficients (**e**) in early-onset PE were highlighted across 1000 differential analyses, with the upper 95% CI of adjusted p-values less than 0.05. The mean of fold changes for 1000 differential analyses between early-onset PE samples and controls was calculated. Adjusted p-values were obtained by the two-sided Wilcoxon rank sum test and corrected using the Benjamini-Hochberg (BH) method for multiple comparisons. **b, f**, Enrichment analysis. Enrichment analysis of pathways and biological processes was performed on genes associated with differential TSS scores (**b**) or Gini coefficients (**f**) identified in early-onset PE samples. **c, g**, DisGeNET analysis. DisGeNET analysis of genes with differential TSS scores (**c**) or Gini coefficients (**g**) between early-onset PE samples and controls identified in **a**, or **e**. P-values for functional enrichment were calculated using the accumulative hypergeometric test in Metascape. **d**, **h**, Similar to **Fig. 3e**, boxplots depict TSS scores or TSS coverages and Gini coefficients at TSS regions of specific genes enriched for "Mean blood pressure" or "Diastolic blood pressure" in the DisGeNET database. For example, *NT5C2* (**d**) and *PLEKHH1* (**h**) genes showed significant differences in early-onset PE samples compared to controls. P-values in boxplots were obtained via the two-sided Wilcoxon rank sum test with no correction for multiple comparisons. Panel (**d, h**) includes 79 early-onset PE samples (EPE) and 230 healthy control samples (CTRL). Box plots (**d, h**) show the median (centre line, 50 th percentile), with box bounds representing the 25 th (first quartile, Q1) and 75 th (third quartile, Q3) percentiles. Whiskers extend from the minimum to maximum values within Q1-1.5×IQR and Q3+1.5×IQR, where IQR is the interquartile range. Source data are provided as a Source Data file.

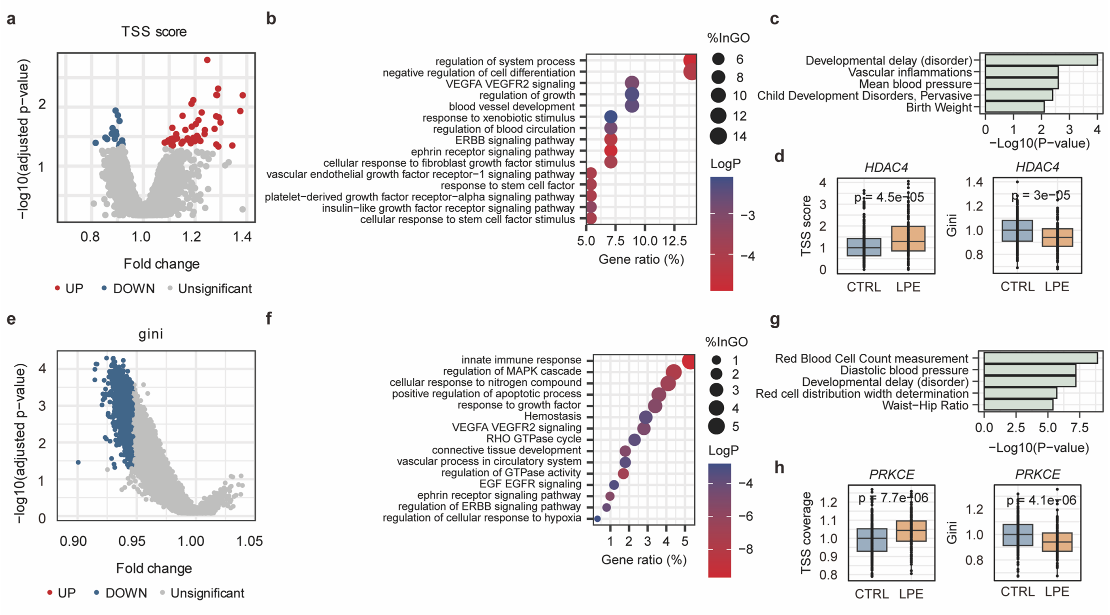

Supplementary Fig. 4|Differential cfDNA fragmentomics analysis between late-onset PE and healthy controls. a, e, Similar to **Fig. 3b**, differential TSS scores and Gini coefficients between late-onset PE samples and controls. TSS regions with significant higher and lower TSS scores (**a**) or Gini coefficients (**e**) in late-onset PE were highlighted across 1000 differential analyses, with the upper 95% CI of adjusted p-values less than 0.05. The mean of fold changes for 1000 differential analyses between late-onset PE samples and controls was calculated. Adjusted p-values were obtained by the two-sided Wilcoxon rank sum test and corrected using the Benjamini-Hochberg (BH) method for multiple comparisons. **b, f**, Enrichment analysis. Enrichment analysis of pathways and biological processes was performed on genes associated with differential TSS scores (**b**) or Gini coefficients (**f**) identified in late-onset PE samples. **c, g**, DisGeNET analysis. DisGeNET analysis of genes with differential TSS scores (**c**) or Gini coefficients (**g**) between late-onset PE samples and controls identified in **a**, or **e**. P-values for functional enrichment were calculated using the accumulative hypergeometric test in Metascape. **d**, **h**, Similar to **Fig. 3e**, boxplots depict TSS scores or TSS coverages and Gini coefficients at TSS regions of specific genes enriched for “Mean blood pressure” or “Diastolic blood pressure” in the DisGeNET database. For example, *HDAC4* (**d**) and *PRKCE* (**h**) genes showed significant differences in late-onset PE samples compared to controls. P-values in boxplots were obtained via the two-sided Wilcoxon rank sum test with no correction for multiple comparisons. Panel (**d, h**) includes 143 late-onset PE samples (LPE) and 230 healthy control samples (CTRL). Box plots (**d, h**) show the median (centre line, 50 th percentile), with box bounds representing the 25 th (first quartile, Q1) and 75 th (third quartile, Q3) percentiles. Whiskers extend from the minimum to maximum values within Q1-1.5×IQR and Q3+1.5×IQR, where IQR is the interquartile range. Source data are provided as a Source Data file.

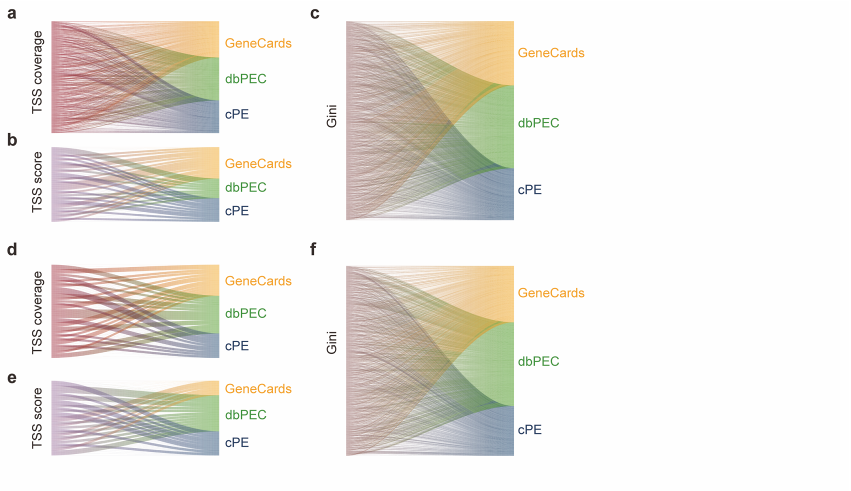

Supplementary Fig. 5|Databases annotation for differential cfDNA fragmentomic features. a-c, The annotation of databases for genes corresponding to differential TSS coverages (**a**), TSS scores (**b**), or Gini coefficients (**c**) between pregnant women with early-onset PE and healthy controls identified in Fig. **3b**, and Supplementary Fig. **3a, e**. **d-f**, Similar to **a-c**, the annotation of databases for genes corresponding to differential TSS coverages (**d**), TSS scores (**e**), or Gini coefficients (**f**) between pregnant women with late-onset PE and healthy controls identified in Fig. **3f**, and Supplementary Fig. **4a, e**. Source data are provided as a Source Data file.

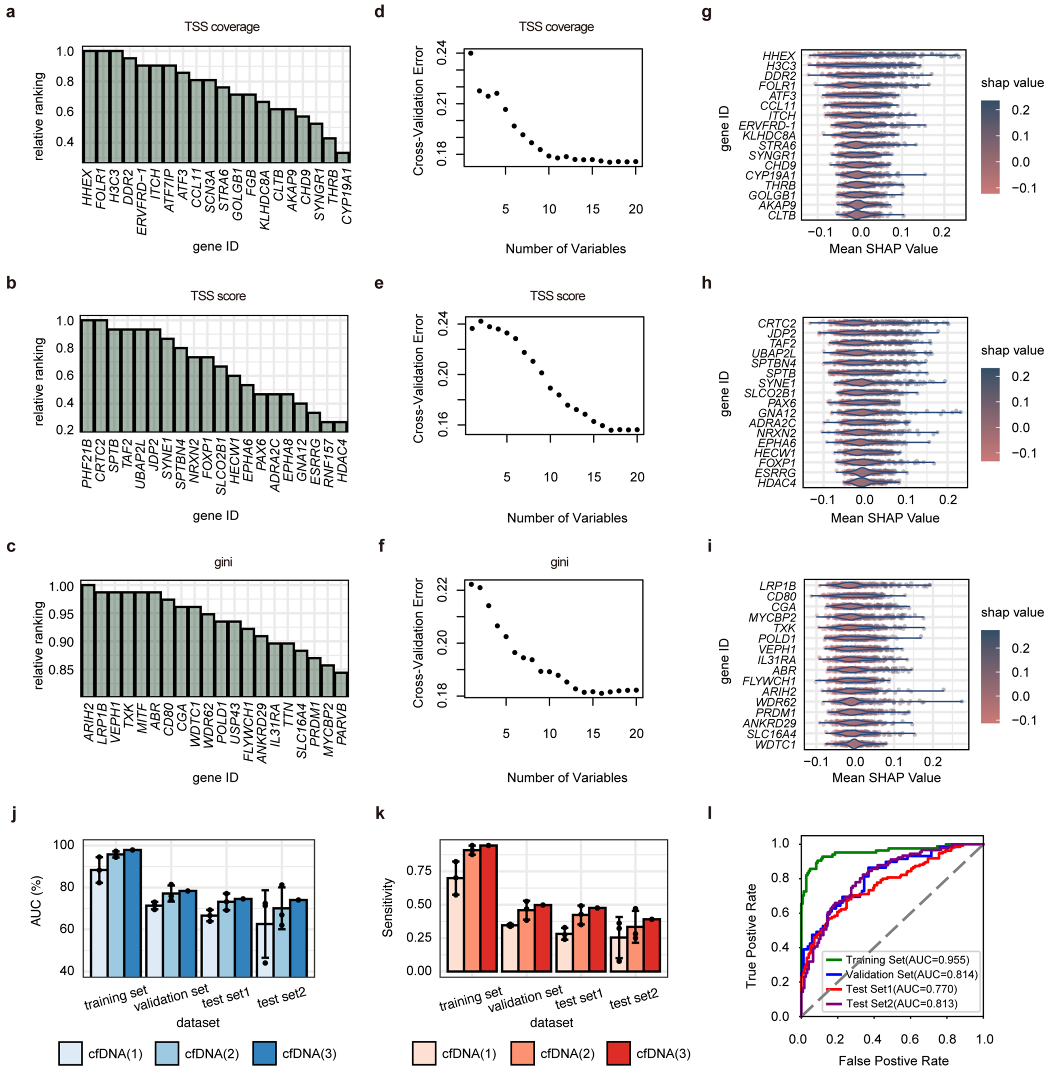

Supplementary Fig. 6|Feature selection for each cfDNA fragmentomic and late-onset PE prediction. Similar to Fig.4, **a-c**, corresponding gene ranking for top 20 TSS coverages (**a**), TSS scores (**b**), and Gini coefficients (**c**) according to their feature importance for each cfDNA fragmentomic. **d-f**, Dot plots depict the mean squared error across each fold during the cross-validation process for models built with distinct subsets of predictors (Methods). The subsets of predictors, ranging in size from 1 to 20, were derived from the top 20 TSS coverages, TSS scores, and Gini coefficients as shown in **a-c**. **g-i**, The SHAP summary plots depict the distribution of contributions of TSS coverages (**g**), TSS scores (**h**), and Gini coefficients (**i**) to late-onset PE prediction, respectively. The y-axis represented genes corresponding to TSS coverages, TSS scores or Gini coefficients of the optimal subsets in **d-f**. **j**, The barplots depict the mean area under the curve (AUC) of late-onset PE models based on different combinations of cfDNA fragmentomic types in the training set, validation set and test sets. **k**, The barplots depict the mean sensitivity of late-onset PE models based on different combinations of cfDNA fragmentomic types in the training set, validation set, and test sets at a false positive rate of 10%. The error bar plots in **j-k** represent the mean ± standard deviation of AUCs or sensitivities for models constructed using one, two, or

all three types of cfDNA fragmentomics. The number of models constructed using one, two, or all three types of cfDNA fragmentomics is three, three, and one, respectively. Specifically, "cfDNA (1)", "cfDNA (2)", and "cfDNA (3)" indicate models that incorporate one, two, and three fragmentomics types, respectively. The "cfDNA (2)" group includes three models built with two types of cfDNA fragmentomics: 1) 17 TSS coverages and 17 TSS scores, 2) 17 TSS coverages and 16 Gini coefficients, 3) 17 TSS scores and 16 Gini coefficients. Similarly, "cfDNA (1)" includes models using each individual fragmentomic type separately, and "cfDNA (3)" refers to models that combine all three types. The detailed performance metrics of each model are provided in Supplementary Data 17. **l**, ROC curves of the optimal late-onset PE model based on cfDNA fragmentomics in the training set, validation set, and test sets. Source data are provided as a Source Data file.

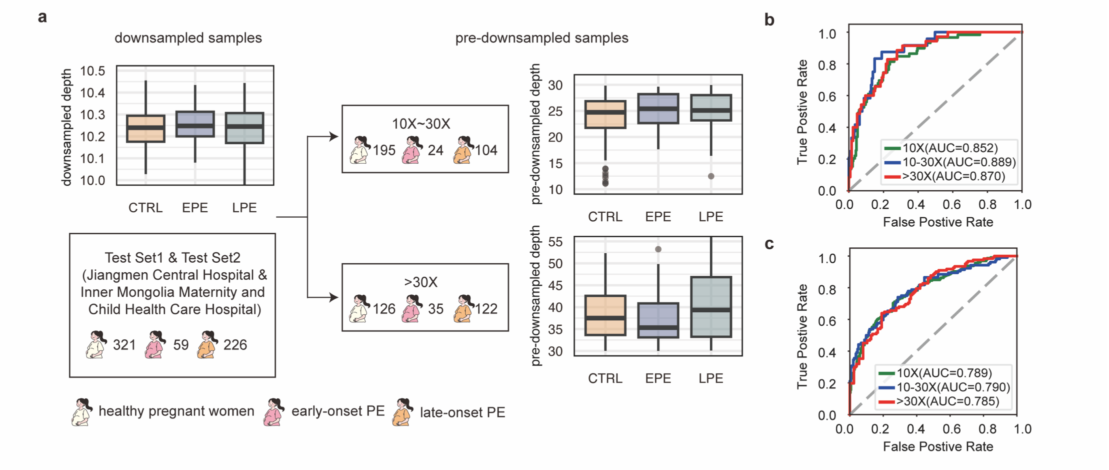

Supplementary Fig. 7|The performance of optimal models for predicting early-onset PE and late-onset PE across read depths greater than 10×. (**a**) Sample redistribution. The schema illustrates the redistribution of test set samples into different datasets based on their pre-downsampled read depths for cfDNA fragmentomics analysis. Boxplots show the distribution of read depth for each dataset. Box plots show the median (centre line, 50 th percentile), with box bounds representing the 25 th (first quartile, Q1) and 75 th (third quartile, Q3) percentiles. Whiskers extend from the minimum to maximum values within Q1-1.5×IQR and Q3+1.5×IQR, where IQR is the interquartile range. CTRL: control; EPE: early-onset preeclampsia; LPE: late-onset preeclampsia. (**b-c**) Model performance. ROC curves of the optimal early-onset PE model (**b**) and late-onset PE model (**c**) based on cfDNA fragmentomics across different read depths. Source data are provided as a Source Data file.

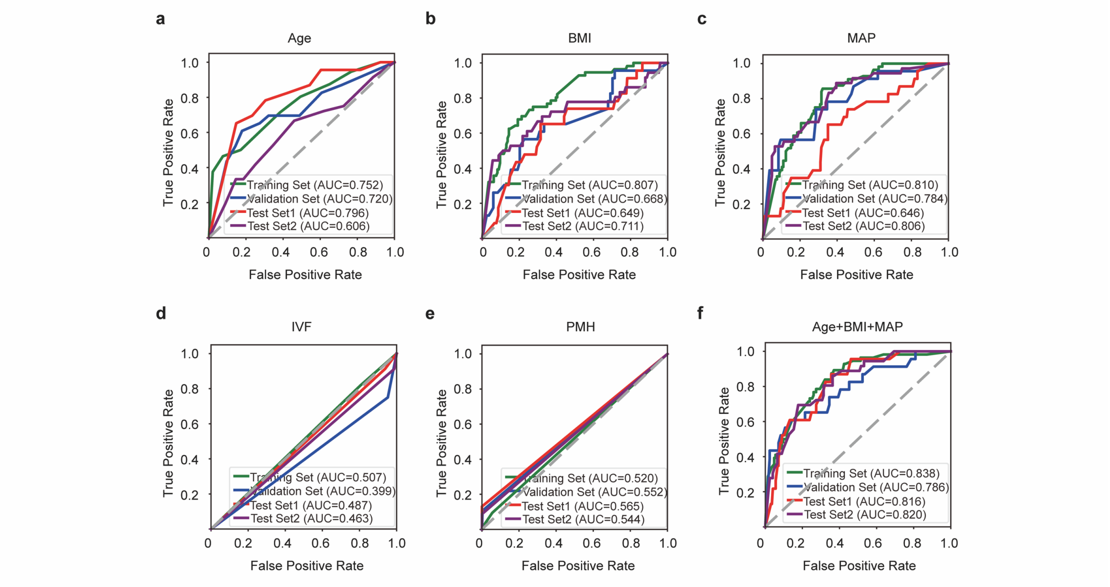

Supplementary Fig. 8| The performance of early-onset PE models based on maternal factors. ROC curves of early-onset PE models based on maternal age (**a**), body mass index (BMI) (**b**), mean arterial pressure (MAP) (**c**), in vitro fertilization (IVF) (**d**), past medical history (PMH) (**e**), and a combination of age, BMI and MAP (**f**) in the training set, validation set, test set1 and test set2. Source data are provided as a Source Data file.

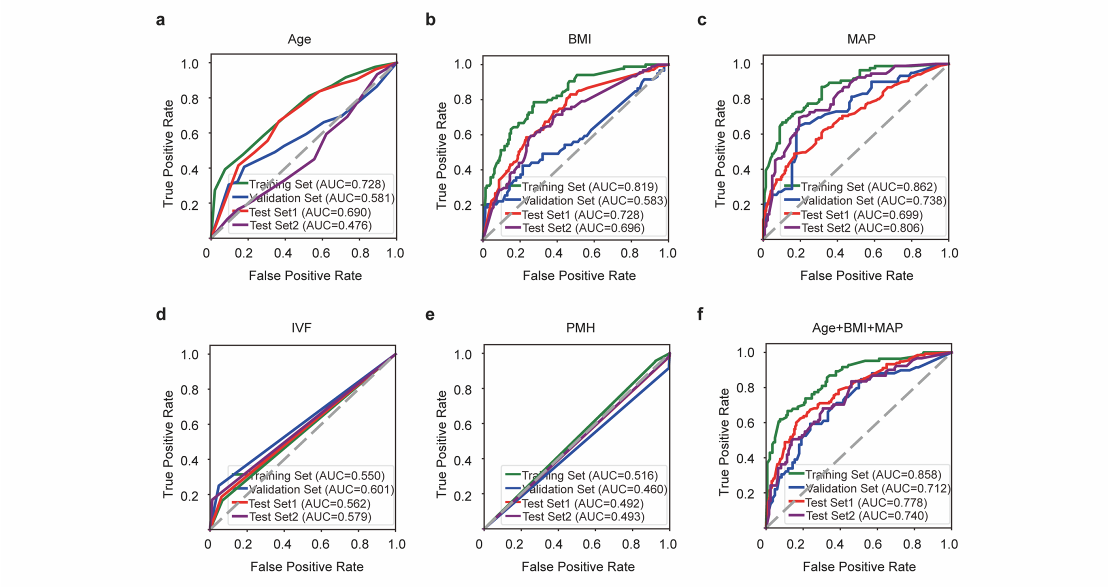

Supplementary Fig. 9| The performance of late-onset PE models based on maternal factors. ROC curves of late-onset PE models based on maternal age (**a**), body mass index (BMI) (**b**), mean arterial pressure (MAP) (**c**), in vitro fertilization (IVF) (**d**), past medical history (PMH) (**e**), and a combination of age, BMI and MAP (**f**) in the training set, validation set, test set1 and test set2. Source data are provided as a Source

Data file.

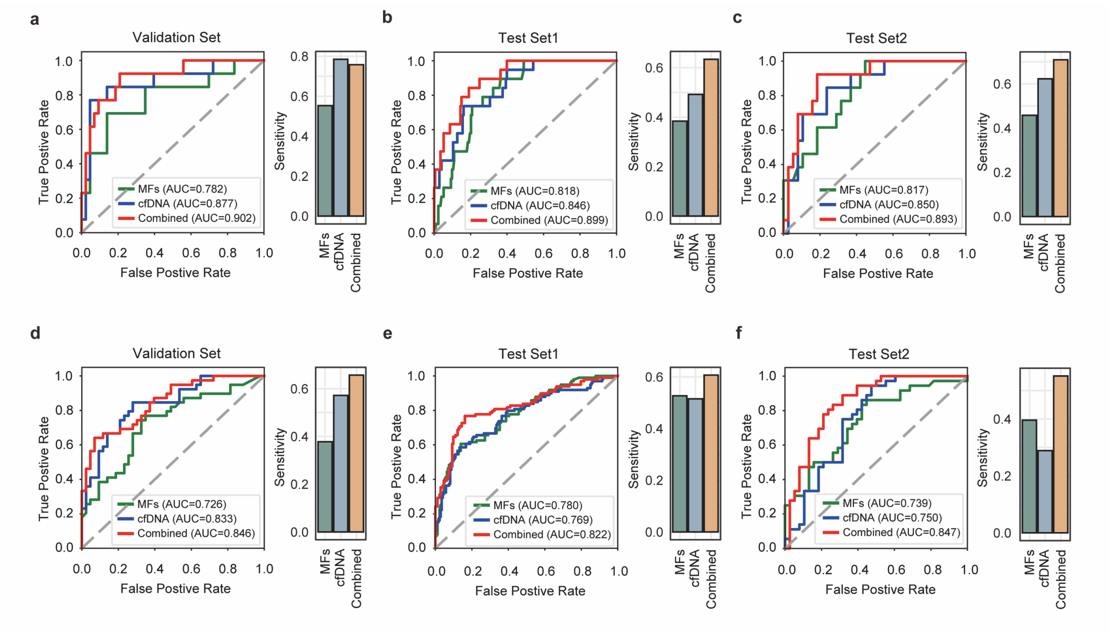

Supplementary Fig. 10| CfDNA fragmentomics improving PE prediction for samples obtained before or at 16 weeks of gestation. a-c, Similar to **Fig. 5f-h**, The plots on the left show ROC curves of early-onset PE models based on cfDNA fragmentomics or maternal factors (MFs) for samples collected before or at 16 weeks of gestation in the validation set (**a**), test set1 (**b**), and test set2 (**c**). The barplots on the right depict sensitivities of early-onset PE models based on cfDNA fragmentomics or MFs in the validation set (**a**), test set1 (**b**), and test set2 (**c**), at a false positive rate of 10%. **d-f**, Similar to **Fig. 5i-k**, The plots on the left show ROC curves of late-onset PE models based on cfDNA fragmentomics or MFs for samples collected before or at 16 weeks of gestation in the validation set (**d**), test set1 (**e**), and test set2 (**f**). The barplots on the right depict sensitivities of early-onset PE models based on cfDNA fragmentomics or MFs in the validation set (**d**), test set1 (**e**), and test set2 (**f**), at a false positive rate of 10%. Source data are provided as a Source Data file.

Supplementary Table 1. Maternal characteristics of early-onset PE and healthy pregnancies in the test sets

	Test Set1			Test Set2		
	Control (n=197)	EPE (n=23)	Pvalue	Control (n=124)	EPE (n=36)	Pvalue
Maternal age (years)	29.00±3.54	35.00±5.03	6.21e-7	31.00±3.61	32.00±4.17	0.11
Gestational age						
at sampling (weeks)	14.00±1.80	13.60±1.56	0.40	16.35±1.40	16.40±2.16	0.89
at delivery (weeks)	39.00±2.46	34.00±4.03	3.13e-13	39.40±2.66	37.05±2.61	3.67e-12
at onset (weeks)	-	29.40±3.44	-	-	30.25±3.71	-
Height (cm)	160.00±5.51	159±5.94	0.44	163.00±5.48	163.50±5.21	0.41
Weight (kg)	54.00±9.87	60.00±7.72	2.61e-3	57.00±9.30	68.90±15.78	1.73e-6
BMI (kg/m^2)	21.23±3.64	23.83±2.62	4.48e-4	21.46±3.05	25.15±5.22	2.83e-7
MAP	86.82±7.90	91.32±8.57	0.01	85.74±9.78	101.40±10.48	1.07e-9
Past medical history	2 (1.02%)	2 (8.70%)	0.06	0 (0.00%)	2 (5.56%)	0.05
Conception method						
In vitro fertiliaztion	3 (1.52%)	2 (8.70%)	0.10	13 (10.48%)	7 (19.44%)	0.27
Natural	194 (98.48%)	21 (91.30%)	0.87	111 (89.52%)	29 (80.56%)	0.78
Parity						
>0	68 (34.52%)	11 (47.83%)	0.41	26 (20.97%)	6 (16.67%)	0.82
>1	2 (1.02%)	1 (4.35%)	0.29	2 (1.61%)	0 (0.00%)	1.00
Gravidity						
>1	88 (44.67%)	18 (78.26%)	0.11	52 (41.94%)	17 (47.22%)	0.74
>2	29 (14.72%)	10 (43.48%)	0.02	19 (15.32%)	7 (19.44%)	0.62

Data were shown as median ± standard deviation values of clinical characteristics or numbers (percentages) of samples. Abbreviations: EPE, early-onset preeclampsia; BMI, body mass index; MAP, mean arterial pressure. The two-sided Fisher's exact test was used to compare gravidity, parity, past medical history and method of conception between PE samples and healthy controls. The two-sided Wilcoxon rank sum test was utilized to compare maternal age, BMI, and MAP between PE samples and healthy controls. Source data are provided as a Source Data file.

Supplementary Table 2. Maternal characteristics of late-onset PE and healthy pregnancies in the training and validation sets

	Training Set			Validation Set		
	Control (n=146)	LPE (n=84)	Pvalue	Control (n=84)	LPE (n=59)	Pvalue
Maternal age (years)	30.00±3.02	32.00±3.69	1.63e-8	30.00±3.76	31.00±4.86	0.05
Gestational age						
at sampling (weeks)	16.10±1.71	15.55±1.82	0.11	15.80±2.56	15.30±1.86	0.06
at delivery (weeks)	39.20±0.87	36.00±1.15	1.49e-36	39.00±0.911	38.00±1.62	5.74e-3
at onset (weeks)	-	35.40±0.83	-	-	37.10±1.84	-
Height (cm)	163.00±5.21	161.65±4.55	0.08	160.00±5.15	159.00±4.69	0.12
Weight (kg)	55.00±6.74	61.90±11.02	4.78e-9	55.00±7.49	56.00±9.79	0.49
BMI (kg/m^2)	20.33±2.26	23.92±3.70	4.47e-13	21.25±2.58	21.78±3.99	0.11
MAP	82.98±10.28	99.54±11.98	4.46e-16	81.46±8.83	90.46±10.00	2.18e-7
Past medical history	0 (0.00%)	7 (8.33%)	1.06e-3	0 (0.00%)	1 (1.69%)	0.42
Conception method						
In vitro fertiliaztion	9 (6.16%)	21 (25.00%)	6.28e-4	1 (1.19%)	11 (18.64%)	1.26e-3
Natural	137 (93.84%)	63 (75.00%)	0.31	83 (98.81%)	48 (81.36%)	0.46
Parity						
>0	55 (37.67%)	17 (20.24%)	0.05	29 (34.52%)	24 (40.58%)	0.63
>1	3 (2.05%)	2 (2.38%)	1.00	3 (3.57%)	5 (8.47%)	0.28
Gravidity						
>1	78 (53.42%)	26 (30.95%)	0.04	37 (44.05%)	27 (45.76%)	1.00
>2	31 (21.23%)	15 (17.86%)	0.74	14 (16.67%)	16 (27.12%)	0.23

Data were shown as median ± standard deviation values of clinical characteristics or numbers (percentages) of samples. Abbreviations: LPE, late-onset preeclampsia; BMI, body mass index; MAP, mean arterial pressure. The two-sided Fisher's exact test was used to compare gravidity, parity, past medical history and method of conception between PE samples and healthy controls. The two-sided Wilcoxon rank sum test was utilized to compare maternal age, BMI, and MAP between PE samples and healthy controls. Source data are provided as a Source Data file.

Supplementary Table 3. Maternal characteristics of late-onset PE and healthy pregnancies in the test sets

	Test Set1			Test Set2		
	Control (n=197)	LPE (n=135)	Pvalue	Control (n=124)	LPE (n=91)	Pvalue
Maternal age (years)	29.00±3.54	32.00±4.59	1.29e-8	31.00±3.61	30.00±3.94	0.29
Gestational age						
at sampling (weeks)	14.00±1.80	14.30±1.72	0.08	16.35±1.40	16.40±1.51	0.51
at delivery (weeks)	39.00±2.46	37.50±1.69	1.13e-13	39.40±2.66	38.10±1.34	5.97e-11
at onset (weeks)	-	37.30±1.88	-	-	37.30±1.70	-
Height (cm)	160.00±5.51	160.00±5.51	0.37	163.00±5.48	162.00±4.86	0.83
Weight (kg)	54.00±9.87	60.00±12.07	1.41e-7	57.00±9.30	62.60±12.60	2.99e-5
BMI (kg/m^2)	21.23±3.64	23.31±4.49	6.39e-9	21.46±3.05	23.88±4.56	2.86e-6
MAP	86.82±7.90	93.02±9.99	1.00e-8	85.74±9.78	99.23±8.53	6.53e-16
Past medical history	2 (1.02%)	2 (1.48%)	1.00	0 (0.00%)	7 (7.69%)	2.89e-3
Conception method						
In vitro fertiliaztion	3 (1.52%)	23 (17.04%)	1.67e-6	13 (10.48%)	6 (6.59%)	0.47
Natural	194 (98.48%)	112 (82.96%)	0.33	111 (89.52%)	85 (93.41%)	0.84
Parity						
>0	68 (34.52%)	34 (25.19%)	0.20	26 (20.97%)	13 (14.29%)	0.38
>1	2 (1.02%)	5 (3.70%)	0.13	2 (1.61%)	1 (1.10%)	1.00
Gravidity						
>1	88 (44.67%)	84 (62.22%)	0.09	52 (41.94%)	35 (38.46%)	0.80
>2	29 (14.72%)	44 (32.59%)	2.69e-3	19 (15.32%)	15 (16.48%)	0.85

Data were shown as median ± standard deviation values of clinical characteristics or numbers (percentages) of samples. Abbreviations: LPE, late-onset preeclampsia; BMI, body mass index; MAP, mean arterial pressure. The two-sided Fisher's exact test was used to compare gravidity, parity, past medical history and method of conception between PE samples and healthy controls. The two-sided Wilcoxon rank sum test was utilized to compare maternal age, BMI, and MAP between PE samples and healthy controls. Source data are provided as a Source Data file.
