# Supplementary material for: Cell-free DNA fragmentomics for preeclampsia risk assessment
Source: Nat Commun. 2026 May 2;17:5957. doi: 10.1038/s41467-026-72682-4 (PMC13342102; doi:10.1038/s41467-026-72682-4)
Supplement: Supplementary file 27 — Reporting Summary [file 41467_2026_72682_MOESM27_ESM.pdf]

Corresponding author(s): Chenming Xu
Last updated by author(s): Feb 20, 2025

Reporting Summary

Nature Portfolio wishes to improve the reproducibility of the work that we publish. This form provides structure for consistency and transparency in reporting. For further information on Nature Portfolio policies, see our Editorial Policies and the Editorial Policy Checklist.

Statistics

For all statistical analyses, confirm that the following items are present in the figure legend, table legend, main text, or Methods section.

n/a	Confirmed	
☐	☒	The exact sample size (n) for each experimental group/condition, given as a discrete number and unit of measurement
☐	☒	A statement on whether measurements were taken from distinct samples or whether the same sample was measured repeatedly
☐	☒	The statistical test(s) used AND whether they are one- or two-sided<br>*Only common tests should be described solely by name; describe more complex techniques in the Methods section.*
☐	☒	A description of all covariates tested
☐	☒	A description of any assumptions or corrections, such as tests of normality and adjustment for multiple comparisons
☐	☒	A full description of the statistical parameters including central tendency (e.g. means) or other basic estimates (e.g. regression coefficient) AND variation (e.g. standard deviation) or associated estimates of uncertainty (e.g. confidence intervals)
☐	☒	For null hypothesis testing, the test statistic (e.g. F, t, r) with confidence intervals, effect sizes, degrees of freedom and P value noted<br>*Give P values as exact values whenever suitable.*
☒	☐	For Bayesian analysis, information on the choice of priors and Markov chain Monte Carlo settings
☒	☐	For hierarchical and complex designs, identification of the appropriate level for tests and full reporting of outcomes
☐	☒	Estimates of effect sizes (e.g. Cohen's d, Pearson's r), indicating how they were calculated

Our web collection on statistics for biologists contains articles on many of the points above.

Software and code

Policy information about availability of computer code

Data collection	All samples analyzed in this study were retrospectively collected from 1,058 pregnant women aged 20-45 years who were enrolled at four hospitals in China from Jan 1, 2019 to Jun 30, 2022, including the Obstetrics & Gynecology Hospital of Fudan University, Zhuhai Center for Maternal and Child Health Care, Jiangmen Central Hospital, and Inner Mongolia Maternity and Child Health Care Hospital. Libraries of cfDNA WGS were sequenced on the DNBSEQ-T7 platform (MGI Tech Co, Ltd), using multiplex sequencing, producing paired-end 100 bp (PE100) reads at a depth of 20-40×.
Data analysis	Adaptors were trimmed from the 3' end of the sequencing reads using SOAPnuke filter (2.1.7). Then, clean reads were aligned to the GRCh38 human reference (GCA_000001405.15) using BWA (0.7.17-r1188). To ensure reliable downstream analyses, non-unique reads, duplicate reads, and reads with supplementary or secondary alignments were excluded using SAMTOOLS (1.15.1). Only reads with specific Sequence Alignment/Map (SAM) flags (81, 83, 97, 99, 145, 147, 161, and 163) were retained for cfDNA fragmentomics analysis, which correspond to properly paired, uniquely mapped, and high-quality read pairs in a paired-end sequencing dataset. All codes and figure source data in this study have been deposited in Code Ocean and are publicly available at https://doi.org/10.24433/CO.7028212.v1.

For manuscripts utilizing custom algorithms or software that are central to the research but not yet described in published literature, software must be made available to editors and reviewers. We strongly encourage code deposition in a community repository (e.g. GitHub). See the Nature Portfolio guidelines for submitting code & software for further information.

Data

Policy information about availability of data

All manuscripts must include a data availability statement. This statement should provide the following information, where applicable:

- Accession codes, unique identifiers, or web links for publicly available datasets
- A description of any restrictions on data availability
- For clinical datasets or third party data, please ensure that the statement adheres to our policy

The BED files for cfDNA fragmentomics analysis in pregnant individuals have been deposited in the OMIX, which is part of the Genome Sequence Archive (GSA) infrastructure for human omics data, under accession number OMIX012350. The dataset is available at: https://ngdc.cncb.ac.cn/omix/release/OMIX012350. In accordance with the current regulations of the People's Republic of China on the administration of human genetic resources (https://flk.npc.gov.cn/index.html), the raw genomic sequencing data contain potentially identifiable genetic information and are therefore available under controlled access. Requests for access should be directed to the corresponding authors and must include a brief description of the intended research purpose and plan. Requests will be reviewed within two weeks. Access will be granted upon approval and completion of a formal data access agreement, which restricts use to non-commercial research purposes and prohibits redistribution to third parties without prior written consent. The key supporting data generated in this study are provided in the article, Supplementary Information and Source data files. Source data are provided with this paper.

Research involving human participants, their data, or biological material

Policy information about studies with human participants or human data. See also policy information about sex, gender (identity/presentation), and sexual orientation and race, ethnicity and racism.

Reporting on sex and gender	All participants are pregnant women.
Reporting on race, ethnicity, or other socially relevant groupings	All participants are pregnant women of Chinese descent.
Population characteristics	The participants include 507 pregnant women who developed early-onset PE (n=138) and late-onset PE (n=369), and 551 healthy controls.
Recruitment	All samples analyzed in this study were retrospectively collected from 1,058 pregnant women aged 20-45 years who were enrolled at four hospitals in China from Jan 1, 2019 to Jun 30, 2022, including the Obstetrics & Gynecology Hospital of Fudan University, Zhuhai Center for Maternal and Child Health Care, Jiangmen Central Hospital and Inner Mongolia Maternity and Child Health Care Hospital.
Ethics oversight	This study was approved by the Ethics Committee of Beijing Genomics Institute (BGI) (BGI-IRB 22134-T1), as well as the respective Ethics Committees of the participating hospitals: Obstetrics & Gynecology Hospital of Fudan University (2023-56), Zhuhai Center for Maternal and Child Health Care ([2022]01), Jiangmen Central Hospital ([2022]02), and Inner Mongolia Maternity and Child Health Care Hospital ([2023]090). All participants provided written informed consent.

Note that full information on the approval of the study protocol must also be provided in the manuscript.

Field-specific reporting

Please select the one below that is the best fit for your research. If you are not sure, read the appropriate sections before making your selection.

☒ Life sciences ☐ Behavioural & social sciences ☐ Ecological, evolutionary & environmental sciences

For a reference copy of the document with all sections, see nature.com/documents/nr-reporting-summary-flat.pdf

Life sciences study design

All studies must disclose on these points even when the disclosure is negative.

Sample size	The sample size in this study was determined based on clinical representativeness and study design requirements rather than a formal statistical power calculation, and the rationale is detailed as follows: A total of 1,058 participants were included in this study. To ensure the clinical generalizability of our sample collection, pregnant women aged 20–45 years in the disease group were strictly recruited from four hospitals between January 1, 2019 and June 30, 2022, in accordance with established diagnostic criteria for preeclampsia (PE). Gestation-matched control participants were randomly selected, with a case-to-control ratio of approximately 1:1.
Data exclusions	No data were excluded from this study.
Replication	Samples from each hospital were analyzed as separate independent datasets. Two additional hospitals were designated as two completely independent test sets to ensure a more accurate validation of the PE model performance.
Randomization	For healthy controls, we randomly selected 146, 84, 197 and 124 plasma samples from pregnant women at four hospitals whose gestational age matched that of pregnant women with early-onset and late-onset PE, respectively.

Blinding	The investigators were blinded to group allocation during data collection and/or analysis

Reporting for specific materials, systems and methods

We require information from authors about some types of materials, experimental systems and methods used in many studies. Here, indicate whether each material, system or method listed is relevant to your study. If you are not sure if a list item applies to your research, read the appropriate section before selecting a response.

Materials & experimental systems

n/a	Involved in the study
☒	☐ Antibodies
☒	☐ Eukaryotic cell lines
☒	☐ Palaeontology and archaeology
☒	☐ Animals and other organisms
☒	☐ Clinical data
☒	☐ Dual use research of concern
☒	☐ Plants

Methods

n/a	Involved in the study
☒	☐ ChIP-seq
☒	☐ Flow cytometry
☒	☐ MRI-based neuroimaging

Plants

Seed stocks	*Report on the source of all seed stocks or other plant material used. If applicable, state the seed stock centre and catalogue number. If plant specimens were collected from the field, describe the collection location, date and sampling procedures.*
Novel plant genotypes	*Describe the methods by which all novel plant genotypes were produced. This includes those generated by transgenic approaches, gene editing, chemical/radiation-based mutagenesis and hybridization. For transgenic lines, describe the transformation method, the number of independent lines analyzed and the generation upon which experiments were performed. For gene-edited lines, describe the editor used, the endogenous sequence targeted for editing, the targeting guide RNA sequence (if applicable) and how the editor was applied.*
Authentication	*Describe any authentication procedures for each seed stock used or novel genotype generated. Describe any experiments used to assess the effect of a mutation and, where applicable, how potential secondary effects (e.g. second site T-DNA insertions, mosiacism, off-target gene editing) were examined.*
