# Supplementary material for: Cell-free DNA fragmentomics for preeclampsia risk assessment
Source: Nat Commun. 2026 May 2;17:5957. doi: 10.1038/s41467-026-72682-4 (PMC13342102; doi:10.1038/s41467-026-72682-4)
Supplement: Supplementary file 2 — Peer Review File [file 41467_2026_72682_MOESM2_ESM.pdf]

nature portfolio

Peer Review File

Cell-free DNA fragmentomics for preeclampsia risk assessment

Corresponding Author: Professor Chenming Xu

This file contains all reviewer reports in order by version, followed by all author rebuttals in order by version.

A version of this paper was originally rejected for publication by Nature Communications, however that decision was reconsidered after appeal by the authors.

Version 0:

Reviewer comments:

Reviewer #1

(Remarks to the Author)
The aim of this study was to optimize maternal plasma cell-free DNA (cfDNA) promoter profiling for the accurate screening of preeclampsia. First, the authors have studied the relationship between gene expression in placentas or maternal blood cells as well as three types of cfDNA fragmentomics at transcription start sites in different sequencing depths. At a 10X read depth, an association was detected with reasonable analytical potential. Then, at this threshold, the study proved value for all three types of cfDNA fragmentomics in identifying preeclamptic pregnancies in a case-control design involving 918 patient samples. As a subsequent step, maternal risk factors were also integrated into the predictive models, increasing their value. Overall, the combined models, validated on two separate datasets, had 73.9% sensitivity for early-onset and 58.7% for late-onset preeclampsia at a 9% false positive rate. The authors concluded that these results underscore the potential of cfDNA fragmentomics in clinical preeclampsia risk assessment.
The manuscript has several merits since it uses large datasets to evaluate the three types of established cfDNA fragmentomics in different sequencing depths for preeclampsia diagnostics. The study is well designed, the manuscript is logically written, the overwhelming data is presented in intelligible fashion and the results are interpreted correctly. However, there are several issues which necessitate major revision, which I list here:
1. In spite the thousands of loci analyzed, there was no adjustment for false discovery rate, only nominal p values were presented in all comparisons. This needs to be fundamentally corrected for false discovery rate and all the data reanalyzed accordingly.
2. It is not clear how many loci's TSS were included into the analysis from which the biologically plausible ones were selected using various databases.
3. The selection of differential expression included several steps, which seems to be overcomplicated. Can this be modified for a more simplistic fashion?
4. Several figures lack important information and are not self-explanatory. For example, there is a lack of definition of the gene groups depicted on bar charts on Figure 1A,B,C.
5. The correlation coefficients between gene expressions and fragmentomics indices are very low on Figure 1D (between -0.1 – 0.1 and -0.06 – 0.06), and it is not clear from the figure whether these are significant either.
6. On Figure 1E, a non-linear association is shown between the depth of sequencing and the number of sequencing reads, although it was expected to be linear. Can an explanation be given?
7. The patient samples were collected between 12-26 weeks of gestation in spite it was claimed on lines 428-429 that "blood drawing at 12-16 weeks of pregnancy is challenging for pregnant women to accept." It is not clear how many samples were still collected in the given timeperiod. The distribution of patient samples according to gestational age at blood draw needs to be explained on a (supplementary) figure.
8. The sample collection time-window is very wide. There are huge gestational-age specific changes in maternal/fetal/placental physiology and also in maternal blood biomarker concentrations with advancing gestation. Therefore, either a narrower time-window for blood sampling should have been applied, or the gestational age should have been adjusted in all analyses. Without these, gestational age at sample collection is a huge confounding factor.
9. Since meaningful preeclampsia prevention can be achieved only before 16 weeks of gestation, which the authors also referred for on line 426, a study using samples only collected before 16th weeks of gestation would only be meaningful for preeclampsia screening worldwide.

10. A biochemical blood test (PIGF) combined with ultrasound and maternal risk factors provide better screening measures for preeclampsia (please see in The International Federation of Gynecology and Obstetrics (FIGO) initiative on pre-eclampsia: A pragmatic guide for first-trimester screening and prevention, https://obgyn.onlinelibrary.wiley.com/doi/10.1002/ijgo.12802) than the method described here. In addition, cfDNA fragmentomics seems to be complicated and more expensive compared to this current clinical standard. The authors need to mention these aspects and make a convincing claim in the discussion why their method would be preferable.

Reviewer #2

(Remarks to the Author)
In this paper, the authors present interesting findings that show that simple fragmentomic measures at transcription start sites (coverage, relative coverage depletion, and fragment size entropy) are predictive of preeclampsia. They show results for a large cohort of early- and late-onset PE pregnancies and control samples collected at 12-26 weeks gestation. They also show that the combination of fragmentomics-based features and maternal risk factors allows more accurate predictions than either method alone.

The work in general is interesting and seems relevant for the field of prenatal diagnostics. There are, however, several major issues:

Overall, the manuscript lacks clarity, and some aspects are not easy to follow.

This is especially true for the machine learning procedures:
1. It is unclear whether the full training+validation set (80% of all samples) or only the validation set (20% of all samples) were used for feature selection. This will have an impact on the reported performance values on the validation set. If both sets were used for feature selection, this would explain the generally higher performance of the validation vs test set, and needs to be explicitly stated. In that case, the validation set performance would not be very informative, as it is biased.
2. “cross validation” usually implies multiple runs of model testing that are then averaged. Was this done here?
3. Fig 4 g,h) This is unclear to me, but seems important. What is meant by “based on different combinations of cfDNA fragmentonics in the training set, validation set and test set”. Why is the dashed line drawn for the best test dataset (the mean/median seems potentially more appropriate)? Also, in the text, what is meant by “optimal model”? If this means that this is the maximum model performance over the different random dataset splits, this is not an acceptable metric to report. If, on the other hand, this relates to different sets of features, this needs to be explained in more detail.
4. “Furthermore, we observed an increase in AUC with increasing cfDNA fragmentomics.” This is unclear to me.
5. A false positive rate of 9% may seem like a low number to many readers, but if this method is intended for screening applications, this may be too high given the relatively low prevalence. This should be discussed. For instance, are follow-up tests available for cases with positive test results?

Data availability: Raw data should be made available via restricted access databases, and anonymized mapped .bam files and downstream data should be made publicly available.

Code availability: Analysis source code should be made available to the reviewers (and later publicly).

Previous literature regarding the use of cfDNA for PE prediction (especially those using cfRNA and cfDNAme) is insufficiently discussed. There is a performance comparison to the method from De Borre et al., however the authors do not mention the difference in sample collection time. Samples in De Borre et al. were collected 9-14 weeks of gestation, whereas this study deals with samples collected at 12-26 weeks gestation. Indeed, the De Borre et al. study mentions that collection time has a major impact on performance (“Consequently, samples obtained from week 12 onwards showed a better performance, with an AUC of 0.792 versus 0.629 for samples obtained before week 12”). Therefore, it seems this comparison is not entirely fair.

Cross-center analysis: It is good that the authors mention as a limitation of the study that cross-center comparison is not possible, but it is unfortunate that such a comparison is not possible.

Fragmentomics methods: The authors use the Gini coefficient to measure the fragment length diversity. Why did the authors not follow the PFE method described by Esfahani et al? It includes a normalization step that may be important and may therefore lead to improved performance. Also, line 108: This is phrased as if it were a novel concept, but it is not - Esfahani et al should be cited here. In general, the fragmentomics metrics used here are rather simplistic, and it could (optionally) be interesting to explore the use of other methods/tools for fragmentomics analysis (e.g. see those explored in Esfahani et al., or other tools used in oncology).

Minor:
There are several grammar and spelling mistakes. The authors should go through the manuscript carefully to remove those. Also the axis legends etc. need to be polished.

The chosen read depth is confusing. The authors show that read depths around 10x are optimal, but then sequenced the samples to significantly higher depths (20-40x). Why? It should be made clear that the reported performance values (ROC-AUC etc) were reported for more deeply sequenced samples.

Fig 3: To me, the illustration suggested that the analysis was done 1000 * 1000 times, but according to the methods only the sampling is repeated 1000 times. Consider removing the second “repeat 1000 times” annotation under the arrow.

Fig 1: In general, the clarity of this figure could be improved, and it could be made more concise.
f) What is meant by “gene abundances”?
g) “d” as y axis legend is unclear, and I find this plot hard to follow
n) Dots are too large and overlap, so hard to assess.

Methods: This sentence is unclear to me: “TSS regions of transcripts with the same TSS were merged, and redundancy removed”.

Introduction, line 68: Consider rephrasing.

Reviewer #3

(Remarks to the Author)
In the current manuscript the authors explore the utility of “cfDNA fragmentomics” = several cfDNA fragmentomic features for preeclampsia (PE) risk assessment. The similar approach of so called “cfDNA promotor profiling” has been previously explored by Guo et al., 2020 (PMID: 32274292) for detection of different pregnancy complications (including preeclampsia), or for foetal growth restriction (Xu et al., 2021, PMID: 32364311). In the listed publications, as well in the current study, the comparison of coverage profiles in the promoter areas (around the transcriptions start sites, TSS) between cases and controls are used for identification of differential read depths potentially associated with the differential gene expression in the tissues of origin, which are then used to build classifiers to predict pregnancy complications. In the current study, in addition to TSS coverage, the authors also bring TSS score, which corresponds to the read distribution within the TSS region (Chen et al., 2021) and Gini coefficient, relating to the diversity of the cfDNA fragment lengths. If previous studies were performed on low-pass sequencing data (0.3X) generated routinely during NIPT (and, f.e, demonstrated sensitivity of 81.7% at AUC=0.825 (0.770-0.880) for PE prediction in a study by Guo et al., 2020), then in the current study the authors suggest that an average coverage of 10X is required for the proposed fragmentomic analyses. As a result, by using genome-wide cfDNA data sequenced 20-40X from healthy pregnant and cases with early- and late-onset PE, the authors by creating the combined fragmentomic and clinical (maternal factor) feature-built models achieved the “mean sensitivities of 73.9% (area under the curve (AUC)=0.92)) for early-onset and 58.7% (AUC=0.83) for late-onset PE prediction”, respectively.
After reading the results section, it is then clear that the model built using only maternal factors (MF) performs better than fragmentomic-based, and addition of the fragmentomic features slightly improves the model characteristics for PE prediction. The main driving factor based on maternal characteristics might be associated with the distinct clinical characteristics of the cases and control groups? Although both cohorts have the matched gestational age, then the maternal age, BMI, and mean arterial pressure are significantly higher in the PE group, which might drive machine learning MF-based predictions? Also, this might be the biological reason why the predictive power for training vs validation sets is so different for fragmentomic approaches and do not generalize across testing sets, as in pregnant with high BMI, the foetal fraction is normally lower than in healthy pregnancies, moreover, high arterial pressure also affects cfDNA levels, which might hinder or affect the analysis? What do authors think about it?
The important question, what is the added value of the proposed “cfDNA fragmentomics”, which requires 10X depth sequencing and how the authors foresee implementation of such analysis in the clinical routine, considering that such deep sequencing is not routinely used for NIPT?
In general, the paper is vaguely written, and sometimes it’s difficult to follow the flow and interpretation of the results. Abstract would require additional information to introduce the theory of coverage and gene expression behind to the reader and additional explanation on the approaches used in the study are required. For example, the authors refer to “all three types of cfDNA fragmentomics”, but what metrics they refer to are unclear. The introduction section also requires reworking. The intro in the result section is better fit in the introduction section, as after reading the introduction the reader still has no clear picture on the fragmentomic theory behind. Figure legends also should be adapted to make it more clear to the reader to understand the results and the meaning of each plot (check also wording on the plots). Description of early and late PE could also go to introduction section from discussion art.

The paper describes 3 cfDNA fragmentomic features located in/around TSS sites. What is the overlap between TSS regions by using 3 different cfDNA fragmentomic features? If the same genes are differently regulated in PE-affected pregnancies, there should be overlap in TSS regions identified using different fragmentomic features? If not, what could be the reason for it?
Also, although the authors acknowledge that by using the secondary selection of the regions by selection only PE-specific once (database search) and top-ranked once might be a limitation, did the authors also evaluate the performance of the predictive models using the initial list of the differential TSS sites from the discovery phase?

As a first step, the authors “determine a reasonable depth for cfDNA WGS” by sequencing 50X the cfDNA from healthy pregnant to evaluate the required for the analysis sequencing depth. They estimate that the 10X coverage would be required

for the cfDNA fragmentomic analysis, however, then they still proceed with the higher coverage for other samples "Subsequently, we performed WGS (~20-40X) of plasma cfDNA from 476 healthy pregnant women, 109 pregnant women..". Is there any reason behind? Are these samples then down sampled to the 10X cov?

Minor remarks:
- Check the concordance of the presented results? E.g. in discussion:
At this threshold, the early- and late-onset PE models developed in this study based on MFs and cfDNA fragmentomics showed good performances for predicting early- and late-onset PE in the test set (AUCs: 0.923 and 0.828), achieving sensitivities of 68.7% and 58.4%, respectively, at a 9% false-positive rate. In abstract: With a false positive rate of 9%, the combined models exhibited mean sensitivities of 73.9% (area under the curve (AUC)=0.92) for early-onset and 58.7% (AUC=0.83)

- Figure 1 a-c is graphical representation of the study design (overview of the fragmentomic features used for classifiers), which could be combined with the Figure 2?
- Typo: "Among these cfDNA fragnentomic approaches", and later on "fragmentonics" and its variation throughout the text?
- "Among these cfDNA fragnentomic approaches, both the promoter profiling of maternal plasma cfDNA and cfDNA concentration" – does concentration relate to fragmentomics approaches?
- "In plasma, cfDNA levels derived from active promoters in tissues or blood cells are lower than those from inactive promoters due to the presence or absence of nucleosome occupancies. However, this type of cfDNA fragmentomic has not been thoroughly explored for differences in cfDNA levels in plasma at various distances from the transcription start site (TSS)." – Isn't the TSS depth coverage analysis is explored in ref 17? Or what authors refer to?
- How the diversity of cfDNA fragment lengths is calculated? Its unclear from the text and not well described in the methods.
Methods:
- The use of RNA-seq data for blood and placenta is poorly understood (also in result section). How do authors do the correlation analysis?
- Ligated with and "A" tail - what does it mean?
- Explain the samtools FLAG sets used for analysis better.

Version 1:

Reviewer comments:

Reviewer #1

(Remarks to the Author)
The manuscript has been significantly improved according to the suggestions of the reviewers which were followed meticulously by the authors. This is appreciated as well as all the efforts made in this study. However, there are still several issues which require revisions before the paper can be accepted for publication:
1. One of the reviewers questioned why a 9% FPR was used and the authors reasoned that "many studies on PE screening adopt a false positive rate of 10% to calculate sensitivity, which makes it easier to compare results across studies. For these reasons, we chose to use the second approach, focusing on the sensitivity at a 10% false positive rate. In fact, for this study, the sensitivity at a 9% false positive rate is very similar to that at 10%, so we chose to report the 9% false". However, it is not understandable why 9% was chosen and not 10% to enable direct comparisons with other studies. I do not suggest rewriting the whole paper but at least a good reason needs to be provided why exactly 9% was chosen.
2. I pointed out earlier that a simple biochemical blood test (PlGF) combined with ultrasound and maternal risk factors provide better screening measures for PE than the presented very tedious and expensive method. In the meantime, a study was published in Nature Medicine showing the value of low coverage prenatal cfDNA WGS data for the quantification of maternal and fetal tissue signatures using nucleosome accessibility and its potential use for the very good prediction of PE before 16 weeks of gestation. The authors need to discuss their findings considering this newly published study, to compare the strengths and limitations of both methods and the real clinical utility of their methodology in the light of these parallel developments.
3. In Table 1, it would be better to define "parity" and "gravidity" instead of "birth history" and "pregnancy history" to better adhere with the literature.
4. It is confusing that PE was defined by the onset of symptoms, however, Table 1 provides gestational ages at delivery, and EPE medians are almost higher than the 34-week cutoff for EPE. Therefore, I suggest providing gestational ages for both onset and delivery, as well.
5. Starting from line 331 (especially between lines 400-450), overwhelming amounts of numbers are included into the text, which is thus very difficult to follow. This needs to be fundamentally changed.

(Remarks on code availability)

Reviewer #2

(Remarks to the Author)
Several of my concerns were addressed, and the manuscript is clearer now. The multi-center evaluation is especially valuable.

I do still have major concerns especially about unfounded claims that the paper makes:

Major:

- The sequencing depth used in this study is still not presented adequately. In the methods section and in the response to reviewers, the authors mention that samples were sequenced to 20-40x, which I now assume is correct. However, in the abstract (line 49) and discussion (507), the authors write as if all their results were obtained at a threshold of 600 million reads (which they determined as optimal in initial experiments). This is strongly misleading, as it suggests that the classification performance they obtain comes from sequencing at 10x, while in reality it comes from sequencing at 20-40x. I see two options: Either completely rephrase to avoid making such claims, or perform a down-sampling analysis on all sequencing samples (randomly draw fragments to reach 10x coverage), and report all metrics on this down-sampled dataset. In either case, this should also be discussed in the Discussion.

- Line 154: It is claimed: "Overall, these results indicated that at a sequencing depth of 600 million reads (~10X final read depth), TSS coverage, TSS score and Gini coefficient metrics could reliably discriminate genes with different expression levels in tissues and blood.". This is vastly overstated. Even for the strongest and weakest-expressed genes, the boxes of the boxplots overlap. The performance is clearly worse than "reliable discrimination", and this claim needs to be phrased much more conservatively.

- The authors do not intend to share sequencing data due to privacy concerns. However, I do not see a reason not to share .bam file in anonymized form, such that fragmentomics information (start and end coordinates of each fragment) is provided, but the DNA sequence is replaced with the reference sequence. .bed files or similar of fragment start and end coordinates would also suffice.

- Line 391: "Notably, these results were comparable to or even better than the predictive performance of cfDNA methylation (cfDNAme)26, or cfRNA profiles27. " and especially line 512 "The performance of the early-onset PE model outperformed other omics-based approaches26,27. "- It is not adequately discussed that different sampling occurred during different gestational ages, which makes it hard to compare. In my opinion, "outperformed" is not justified here without further in-depth comparisons.

- Code documentation and organization should be improved. For instance, I could not easily find the code for the machine learning procedures.

Minor:
- The impact of a 9% false positive rate in clinical practice should be discussed
- Line 102: Revise grammar
- Table 1: What do the superscript values mean? This is not explained.
- Fig 4 j-k: Real data points should be shown in addition to or instead of the error bars. This would allow assessment whether e.g. the best 2-featuretype model is equal to the 3-feature-tpye model
- Line 430: Provide the performance values or improvement values

(Remarks on code availability)
Code documentation and organization should be improved. For instance, I could not find the code for the machine learning procedures. The workflow is not explained and structured well enough to easily reproduce the results.

E.g. Which input files are needed?

Also, the markdown is not rendered properly on Github

Reviewer #4

(Remarks to the Author)
Thank you for the opportunity to review your paper. I was asked primarily to comment on whether Reviewer 3's comments were addressed.

COMMENT 1: Regarding performance models, impact of maternal factors, etc.
I appreciate the authors attention to responding to this critique, including adding additional cases and sites. Their results do suggest that their approach to incorporate cfDNA fragmentomics improves preeclampsia prediction above maternal risk factors alone. I have a few remaining questions / concerns:
• When were the clinical parameters such as BMI and MAP assessed?
• Fig 4 and Fig 5, you report AUCs of 1.0 for training, with subsequently substantially lower AUCs for validation and test sets. This is a red flag for model overfitting, suggesting that the model has memorized the training data, instead of learning generalizable patterns. This is likely to be an issue with assessment on a broader population (i.e., less "clean" control

group). How do the authors explain this?
• With respect to the “gestational-age matched controls” what were the outcomes of this control group? Did they only include those pregnancies ending in term delivery without hypertensive complications? If so, this would make the control group “cleaner” than what you would expect in a real population (i.e., if they excluded pregnancies ending in preterm birth for other reasons, or complicated by other pregnancy outcomes).
• Fig 4j-k: I found the explanation about what cfDNA(1), (2), and (3) mean still to be confusing. The text suggests that cfDNA(2) includes “two types of cfDNA fragmentomics”, but then your further example includes 3 different combinations of cfDNA fragmentomic features (TSS coverage, TSS scores, Gini coefficients). This needs to be clarified.

COMMENT 2: Concerns regarding need for 10X depth when this is not routinely performed during NIPT.
• I appreciate the importance of sequencing depth, however, a recent publication also utilized cfDNA fragmentomic analysis and these authors used low-coverage whole genome sequencing data (Adil, et al, Nature Medicine, 2025, PMID 39939524) at around 0.5X that also evaluated preeclampsia prediction. This study also included a less “clean” control population. I recommend the authors explain how the reader should position their manuscript in light of this recent publication. What is their approach adding in light of others showing similar results with less sequencing depth and a more "real world" control population?

COMMENT 3: Regarding readability of the paper.
• I did not read the prior versions, but the readability of this manuscript is appropriate – however, see comment regarding confusion with Fig 4 legend. I do recommend changing the icon used to represent pregnant individuals throughout the Figures. The current icon is not pictorially representative of pregnant people and may be considered offensive.

COMMENT 4: Regarding overlap between TSS regions by using 3 different cfDNA fragmentomic features.
• No additional comments here.

COMMENT 5: Regarding evaluation of performance of the predictive models using the initial list of differential TSS sites from the discovery phase.
• No additional comments here.

COMMENT 6: Regarding determination of a "reasonable depth for cfDNA WGS" by sequencing 50X which lead to determining that 10X was sufficient, but then ultimately using 20-40X for their study.
• I agree with the prior reviewer about these concerns and did not find that this was adequately addressed. If you determined that 10X was sufficient, why then do you use 20-40X to develop and evaluate models for predicting PE (Line 625)? This goes back to prior points about translatability about your findings as 10-40X is not standard depth sequencing for NIPT. I appreciate that these are precious samples and you wanted to maximize their use – could you have considered downsampling? To me, this remains a major limitation of your approach and decreasing the innovation potential. Also, you should clearly state the median sequencing depth for each of your cohorts (training, validation, both test sets) as in the current set up, you are stating that 10X is sufficient, but that is not what you ultimately used for PE prediction.

COMMENT 7 (minor remark): Regarding concordance of presented figures.
• No additional comments here, except that I recommend you use a different icon to represent pregnant people.

COMMENT 8 (minor remark): Regarding Figure 1a-c
• No additional comments here, except that I recommend you use a different icon to represent pregnant people.

COMMENT 9 (minor remark): regarding typo
• No additional comments.

COMMENT 10 (minor remark): Regarding concentration.
• No additional comments.

COMMENT 11 (minor remark): Regarding TSS depth coverage
• Figure 2 a-c, for the x-axis for your bar plots, why is there is an arrow? You are looking at two distinct groups – TSS regions of active vs. inactive genes. What is the difference between Fig 2a and 2b? The x and y-axes are the same and the plots of the red and blue lines also look the same. What is the yellow shading and the green shading? Please explain this better in the figure legend.

COMMENT 12 (minor remark): Diversity of cfDNA fragment lengths.
• I had a hard time following what the Gini coefficient was used for? My understanding is that this quantifies diversity of cfDNA fragments in a sample. How did this specific metric help with designing your classifier towards your ultimate goal (PE prediction)? Consider elaborating on this in the Discussion to explain why this was important. Also Line 102 is an incomplete sentence: “The Gini coefficient, which quantifies the size distribution of cfDNA fragments at transcription start sites (TSS).” This is why there is further confusion about what the Gini metric is serving in your study.

COMMMENT 13 (minor remark): Regarding RNA-seq data
• No additional comments.

COMMENT 14 (minor remark): Regarding what "A" tail means

• No additional comments.

COMMENT 15 (minor remark): Regarding samtools FLAG
• No additional comments.

ADDITIONAL COMMENTS:
Line 495 the authors state that “PE prediction before 12 weeks has shown limited efficacy” and they reference the DeBorre paper that evaluated PE prediction using cfDNA methylation. I don’t believe their statement is supported by this reference as it’s unclear what they mean by “efficacy”. Certainly, earlier screening would allow for institution of interventions earlier. So why do they think that earlier screening is not “efficacious”? FMF screening can occur in the first trimester and many consider this to be “efficacious” (hence used in clinical practice). As such, I’m not sure I agree with the authors comment here as currently worded.

(Remarks on code availability)
I did not review the code.

Version 2:

Reviewer comments:

Reviewer #1

(Remarks to the Author)
The authors have performed the requested modifications point-by-point addressing all concerns and the manuscript can now be considered for publication.

(Remarks on code availability)

Reviewer #2

(Remarks to the Author)
A few of my points have not been fully addressed:

1. In the interest of making the data produced in this study maximally useful for other to build on, I still think that it feasible that the authors share .bam file in anonymized form, such that fragmentomics information (start and end coordinates of each fragment) is provided, but the DNA sequence is replaced with the reference sequence, or .bed files or fragment start and end coordinates. This data can easily be generated from the same source data that has been used to generate the new analyses in this revision.

2. I still think that the comparison to the data from De Borre et al. is not entirely fair. The authors now write:
“Notably, our PE 566 models based on cfDNA fragmentomics alone were better than other omics-based approaches26,37. For example, the early-onset PE model based on cfDNA methylation alone showed lower predictive performance for samples collected before 16 weeks of gestation (AUC: 0.752)26, compared to our early-onset PE model based on cfDNA fragmentomics alone, which achieved a higher AUC of 0.852 for samples collected at the same gestational age in test sets. “
See my original comment from the first submission: “Samples in De Borre et al. were collected 9-14 weeks of gestation, whereas this study deals with samples collected at 12-26 weeks gestation. Indeed, the De Borre et al. study mentions that collection time has a major impact on performance (“Consequently, samples obtained from week 12 onwards showed a better performance, with an AUC of 0.792 versus 0.629 for samples obtained before week 12”). Therefore, it seems this comparison is not entirely fair.”
Unless I am missing something, the comparisons the authors make here is on the performance of samples 9-14 weeks (De Borre) and 12-16 weeks (their study). This is very much not the “same gestational age” – only two weeks out of a total of 8 overlap. This needs to be clarified, and the explicit claim “our PE models based on cfDNA fragmentomics alone were better than other omics-based approaches26,37.” is not valid. This is now the third time I am asking for a fair comparison here. Care should be taken with the comparison to ref. 37 as well, which I have not evaluated in detail.
3. “In Table 1, the superscript values represented the gestational age (in days) at the time of sample collection, delivery, and onset of PE. “ I find this a very confusing way of visualizing the data. Consider using separate rows instead.
Additionally:
4. “we observed variability in model performance across different validation datasets when using NIPT data” -> “We observed” is confusing to me, as this sentence is just citing results from other studies. Also, this sentence and the following seem to be somewhat understating the importance of the cited studies.

(Remarks on code availability)

Reviewer #4

(Remarks to the Author)
COMMENT 1-1: Regarding when were the clinical parameters such as BMI and MAP assessed?
No additional comments.

COMMENT 1-2: Regarding concerns about overfitting.
I remain confused about the first aspect of your approach – combining training and validation sets for model selection. How is this maintaining data independence? If you combined the training and validation sets, then should you not just call this your training set; and then Test Set 1 and Test Set 2 are your validation sets? Also, if you combine the training and validation sets for feature selection, how is the reader supposed to interpret Figure 4l, Figure 5 d-f, and Figure 5i?

COMMENT 1-3: Regarding control cohort.
Thank you for this clarification, however, it remains unclear if these participants had uncomplicated pregnancies that delivered at term. The latter part about term deliveries is important and needs to be included in the description of the cohort. Table 1 suggests that these were all term deliveries. Additionally, I would further temper your language in the Discussion as say that this "likely limits the generalizability of our findings to broader clinical populations."

COMMENT 1-4: Regarding Figure 4j-k legend.
No additional comments.

COMMENT 2: Concerns regarding need for 10X depth when this is not routinely performed during NIPT.
Thank you for addressing this. What you're ultimately saying, however, is that your approach does not work well on lower coverage data (below 10X). So although this exploratory approach works on 10X data, performance at lower depths is suboptimal and not comparable to the approach chosen by Adil et al. I think this remains a major and critical limitation of the novelty of your findings in light of this recent publication. You could have mitigated this by further downsampling to 0.5X to compare your results with theirs, but based on your response here, it seems that it will be inferior. Furthermore, the NIPT data used in Adil et al was paired-end sequencing, so your statement at the end of this paragraph is misleading. Overall, this aspect of the manuscript remains a significant issue.

It is also unclear what is meant by, "we observed variability in model performance across different validation datasets using NIPT data". Do you mean that this is the performance reported in these other studies? If so, I would change the language to be clearer (not something you "observed", but what is reported in these other papers). Also, these AUCs (0.80-0.92) are quite favorable, and comparable to your reported AUCs in your "test sets" (0.84-0.85 for early-onset PE, and 0.75-0.77 for late-onset PE). This again suggests that with higher sequencing depth, your approach does not necessarily afford any gains.

Regarding the control population, as discussed in our responses to previous comments (COMMENT 1-3): See comment above about whether these were all term deliveries. This needs to be added to the manuscript.

COMMENT 3: Regarding readability of the paper.
No additional comments.

COMMENT 6: Regarding determination of a "reasonable depth for cfDNA WGS" by sequencing 50X which lead to determining that 10X was sufficient, but then ultimately using 20-40X for their study. I appreciate the changes you've made to downsample to 10X. However, I think the issue remains that your approach works well at 10X, but not lower than this. And this depth is not standard sequencing depth for NIPT. I think you need to clarify if/why your approach offers any advantage over other published studies that have performed cfDNA fragmentomics for PE risk stratification with much lower sequencing depths with comparable performance. It is not surprising that higher depth will provide higher accuracy, so the advantage of your approach is unclear.

COMMENT 11 (minor remark): Regarding TSS depth coverage
No additional comments.

COMMENT 12: Regarding diversity of cfDNA fragment lengths (Gini coefficient)
No additional comments.

ADDITIONAL COMMENTS:
The one thing I will continue to point out here, is that although you correctly report that DeBorre found suboptimal performance before 12 weeks gestation, you also did not sample before this gestational age. In fact, Table 1 indicates that your mean GA at sampling is around 16 weeks. As such, you can't really compare your data to Deborre et al as you did not include samples from that early in gestation (or at least very few). This is also intriguing as if you propose this could be a method offered with NIPT (if sequencing costs continue to decrease), however, you mention that NIPT is routinely performed between 10-12 weeks in China. You also note that an additional blood draw at 12-16 weeks "might be challenging", but this is what your approach would require.

Also, what do you mean by "…using samples obtained during NIPT, as well as those collected before or at 16 weeks of gestation." For the second part, are these samples that were not collected for NIPT? What were these samples collected for?

(Remarks on code availability)

Version 3:

Reviewer comments:

Reviewer #4

(Remarks to the Author)
Comment 1-2:
Thank you for this clarification. No additional comments.

Comment 1-3:
Thank you for editing your text. No additional comments.

Comment 2:
Thank you for editing your text. No additional comments.

Comment 6:
Thank you for editing your text. No additional comments.

Additional comments:
Thank you for this clarification. No additional comments.

(Remarks on code availability)

Open Access This Peer Review File is licensed under a Creative Commons Attribution 4.0 International License, which permits use, sharing, adaptation, distribution and reproduction in any medium or format, as long as you give appropriate credit to the original author(s) and the source, provide a link to the Creative Commons license, and indicate if changes were made.
In cases where reviewers are anonymous, credit should be given to 'Anonymous Referee' and the source.
The images or other third party material in this Peer Review File are included in the article’s Creative Commons license,

unless indicated otherwise in a credit line to the material. If material is not included in the article's Creative Commons license and your intended use is not permitted by statutory regulation or exceeds the permitted use, you will need to obtain permission directly from the copyright holder.
To view a copy of this license, visit https://creativecommons.org/licenses/by/4.0/

Reviewers' comments:

Reviewer #1 (Remarks to the Author):

The aim of this study was to optimize maternal plasma cell-free DNA (cfDNA) promoter profiling for the accurate screening of preeclampsia. First, the authors have studied the relationship between gene expression in placentas or maternal blood cells as well as three types of cfDNA fragmentomics at transcription start sites in different sequencing depths. At a 10X read depth, an association was detected with reasonable analytical potential. Then, at this threshold, the study proved value for all three types of cfDNA fragmentomics in identifying preeclamptic pregnancies in a case-control design involving 918 patient samples. As a subsequent step, maternal risk factors were also integrated into the predictive models, increasing their value. Overall, the combined models, validated on two separate datasets, had 73.9% sensitivity for early-onset and 58.7% for late-onset preeclampsia at a 9% false positive rate. The authors concluded that these results underscore the potential of cfDNA fragmentomics in clinical preeclampsia risk assessment.
The manuscript has several merits since it uses large datasets to evaluate the three types of established cfDNA fragmentomics in different sequencing depths for preeclampsia diagnostics. The study is well designed, the manuscript is logically written, the overwhelming data is presented in intelligible fashion and the results are interpreted correctly. However, there are several issues which necessitate major revision, which I list here:
1. In spite the thousands of loci analyzed, there was no adjustment for false discovery rate, only nominal p values were presented in all comparisons. This needs to be fundamentally corrected for false discovery rate and all the data reanalyzed accordingly.
Author response: Thank you for your helpful comment. We completely agree with the importance of correcting for multiple comparisons. Based on feedback from other reviewers and the editor, we have now divided the datasets by individual hospital and applied the Benjamini-Hochberg FDR correction to our analysis. We have reanalyzed the data accordingly and believe this will improve the robustness of our findings.

2. It is not clear how many loci's TSS were included into the analysis from which the biologically plausible ones were selected using various databases.
Author response: Thank you for your comment. We have now clarified the number of TSS loci included in the third subsection of "Result" section. For early-onset PE prediction, 277 TSS coverages, 163 TSS scores, and 1,687 Gini coefficients were included, while 291 TSS coverages, 147 TSS scores, and 1,809 Gini coefficients were retained for late-onset PE prediction. These features corresponding to genes reported in PE-associated databases, including cPE, dbPEC, or GeneCards.

3. The selection of differential expression included several steps, which seems to be overcomplicated. Can this be modified for a more simplistic fashion?
Author response: Thank you for the suggestion. We understand your concern about the complexity of the differential expression selection process. The reason we used bootstrap repeated sampling 1000 times was to ensure the stability and reproducibility of the selected features, aiming to improve the robustness of our results.

4. Several figures lack important information and are not self-explanatory. For example, there is a lack of definition of the gene groups depicted on bar charts on Figure 1A,B,C.

Author response: Thank you for your feedback regarding the clarity of our figures. Based on suggestions from other reviewers and the editor, we have now updated Figure 1a-c to Figure 2a-c. The bar charts in Figure 2a-c illustrate the results of three different cfDNA fragmentomics metrics: TSS coverage, TSS score and Gini coefficient. These metrics are used to assess coverage patterns and size distributions of cfDNA fragments at 2kb regions of the top 500 active (blue) and top 500 inactive (red) genes, as shown in the left schematic illustration. Here, we would like to emphasize that these figures are schematic representations and not exact reproductions of our results. The purpose of these figures is to offer a conceptual overview of the data rather than to provide detailed numerical results. We have also updated the figure legend to provide clearer description.

5. The correlation coefficients between gene expressions and fragmentomics indices are very low on Figure 1D (between -0.1 – 0.1 and -0.06 – 0.06), and it is not clear from the figure whether these are significant either.

Author response: Thank you for your comment. The low correlation coefficients observed in Figure 1d (between -0.1 to 0.1 and -0.06 to 0.06) are likely due to the use of Pearson correlation analysis on both expressed and unexpressed genes. While the fragmentomics indices, centered around transcription start sites (TSSs), are intended to reflect gene expression, they could also capture DNA fragments resulting from other biological processes, such as degradation. This could explain the weaker correlation between cfDNA fragmentomics and gene expression in placenta or whole blood. This opinion is also discussed in the "Discussion" section. In response to your comment, we have reanalyzed the data using Spearman correlation, which provided stronger correlations, ranging from 0.63 to 0.89 and 0.48 to 0.72. This approach seems to better capture the relationship between the fragmentomics indices and gene expression.

Additionally, we would like to emphasize that the cfDNA fragment levels at TSS regions in plasma represent a combination of multiple biological mechanisms, including nucleosome and transcription factor occupancy, as well as cell death and degradation processes. Therefore, the reliability of cfDNA fragmentomics indices may vary, and higher sequencing depth can help improve their accuracy. We observed that the relationship between gene expression and fragmentomics indices reaches a plateau once sufficient sequencing depth is achieved, with no significant changes observed with further increases in depth. This suggests that the relationship between cfDNA fragmentomics and gene expression has been sufficiently captured at this point and adequate sequencing depth is necessary for analyzing cfDNA fragmentomics.

6. On Figure 1E, a non-linear association is shown between the depth of sequencing and the number of sequencing reads, although it was expected to be linear. Can an explanation be given?

Author response: Thank you for your question. The non-linear association observed occurs because, as sequencing depth increases, a point of saturation is eventually reached, where adding more depth no longer significantly increases the number of unique reads. This is because most of cfDNA fragments have already been sequenced multiple times, and further sequencing does not provide much additional information. Moreover, technical factors, including library preparation protocols, sequencing platforms, and bioinformatics pipelines can also impact the relationship between final

depth (after removing low-quality reads and duplicates) and the number of sequencing reads. These factors can affect the efficiency and accuracy of sequencing, further complicating the expected linear relationship. Given these complexities, determining the appropriate sequencing depth is crucial, especially for applications like liquid biopsy and non-invasive prenatal testing, where accurate detection and quantification of cfDNA are essential.

7. The patient samples were collected between 12-26 weeks of gestation in spite it was claimed on lines 428-429 that “blood drawing at 12-16 weeks of pregnancy is challenging for pregnant women to accept.” It is not clear how many samples were still collected in the given timeperiod. The distribution of patient samples according to gestational age at blood draw needs to be explained on a (supplementary) figure.
Author response: Thank you for pointing out the need for clarification. We have now included a supplementary figure (Supplementary Fig. 2) that illustrates the distribution of plasma samples according to gestational age at the time of blood draw.

8. The sample collection time-window is very wide. There are huge gestational-age specific changes in maternal/fetal/placental physiology and also in maternal blood biomarker concentrations with advancing gestation. Therefore, either a narrower time-window for blood sampling should have been applied, or the gestational age should have been adjusted in all analyses. Without these, gestational age at sample collection is a huge confounding factor.
Author response: Thank you for your insightful comment. Combined both questions 9 and 10, we made overall improvements to our analysis. First, we examined whether cfDNA fragmentomic features selected for constructing the PE prediction model changed significantly with gestational age. We found only a few cfDNA fragmentomic features in optimal early-onset or late-onset PE models showed significant changes as gestation progressed, suggesting that these changes might occur as early as the first trimester (Supplementary Table 18-19). Based on this, we hypothesize that the features we have selected might also be applicable for PE prediction before or at 16 weeks of gestation. To validate this hypothesis, we have now included results from a model using data from 16 weeks and earlier. Only slight declines were observed in the performances of early-onset and late-onset PE models. The early-onset PE model for samples obtained before or at 16 weeks' gestation yielded AUCs of 0.920 (95% CI: 0.918-0.922), 0.835 (95% CI: 0.832-0.838), and 0.834 (95% CI: 0.830-0.839), with sensitivities of 0.651 (95% CI: 0.640-0.663), 0.552 (95% CI: 0.545-0.560), and 0.574 (95% CI: 0.563-0.585) at a 9% FPR across validation and test sets (Fig. 5d). Similarly, the late-onset PE model for samples obtained before or at 16 weeks of gestation displayed comparable performance to samples collected between 12 and 26 weeks of gestation, with AUCs of 0.831 (95% CI: 0.828-0.834), 0.795 (95% CI: 0.793-0.797), and 0.765 (95% CI: 0.761-0.762) (Fig. 5e). Sensitivities at a 9% FPR were 0.535 (95% CI: 0.526-0.543), 0.477 (95% CI: 0.473-0.480), and 0.450 (95% CI: 0.444-0.456) in the validation and test sets, respectively. These contents now have included in the fourth subsection of "Result" section.

9. Since meaningful preeclampsia prevention can be achieved only before 16 weeks of gestation, which the authors also referred for on line 426, a study using samples only collected before 16th weeks of gestation would only be meaningful for preeclampsia screening worldwide.

Author response: We recognize the clinical significance of screening before 16 weeks of gestation. As mentioned in response to question 8, although our study includes samples from 12 to 26 weeks, we have now explored the potential implications of earlier screening. To provide more relevant information, we have included a subgroup analysis of samples collected before or at 16 weeks in the fourth subsection of "Results" section.

10. A biochemical blood test (PlGF) combined with ultrasound and maternal risk factors provide better screening measures for preeclampsia (please see in The International Federation of Gynecology and Obstetrics (FIGO) initiative on pre-eclampsia: A pragmatic guide for first-trimester screening and prevention, https://obgyn.onlinelibrary.wiley.com/doi/10.1002/ijgo.12802) than the method described here. In addition, cfDNA fragmentomics seems to be complicated and more expensive compared to this current clinical standard. The authors need to mention these aspects and make a convincing claim in the discussion why their method would be preferable.
Author response: Thank you for your insightful comments. We fully acknowledge that a biochemical blood test (PlGF) combined with ultrasound and maternal risk factors provide a reliable and widely adopted standard for PE screening and have discussed this method in the "Discussion" section. While cfDNA fragmentomics is indeed more complex and expensive than the standard biochemical tests, we see potential for this approach to offer valuable insights that are not captured by traditional methods. One key advantage is the ability of cfDNA fragmentomics to provide molecular-level information that reflects changes in gene expression and genomic features associated with PE. Furthermore, although the technique is currently more costly, we anticipate that as sequencing technologies become more affordable and accessible, the deeper sequencing depth required for cfDNA fragmentomics could become widely accepted in clinical settings, including for applications such as non-invasive prenatal testing (NIPT).

Reviewer #2 (Remarks to the Author):

In this paper, the authors present interesting findings that show that simple fragmentomic measures at transcription start sites (coverage, relative coverage depletion, and fragment size entropy) are predictive of preeclampsia. They show results for a large cohort of early- and late-onset PE pregnancies and control samples collected at 12-26 weeks gestation. They also show that the combination of fragmentomics-based features and maternal risk factors allows more accurate predictions than either method alone.

The work in general is interesting and seems relevant for the field of prenatal diagnostics. There are, however, several major issues:

Overall, the manuscript lacks clarity, and some aspects are not easy to follow.

This is especially true for the machine learning procedures:
1. It is unclear whether the full training+validation set (80% of all samples) or only the validation set (20% of all samples) were used for feature selection. This will have an impact on the reported

performance values on the validation set. If both sets were used for feature selection, this would explain the generally higher performance of the validation vs test set, and needs to be explicitly stated. In that case, the validation set performance would not be very informative, as it is biased.
Author response: Thank you for your valuable feedback. Combining with advice from other reviewers and editor, we have revised our study design to enhance the robustness and generalizability of our findings (Fig.2). Specifically, we have now divided the datasets by individual hospital to ensure the independence of hospital datasets, which will also strengthen the multi-center applicability of our results. You are correct in pointing out that both the training and validation sets (samples from two hospitals) were used for feature selection, which might have led to the higher performance of the validation vs test sets. To address this and ensure a more accurate validation of the PE model performance, we have now designated data from two additional hospitals as two completely independent test sets, which were not involved in the feature selection process.

2. “cross validation” usually implies multiple runs of model testing that are then averaged. Was this done here?
Author response: Thank you for raising this important point. We agree that the term “cross-validation” may not have been used accurately in the context of our feature selection process. To clarify, we have revised this description to “feature-selection” in Fig.2, in the second subsection of the “Results” section and the “Method” section. For feature selection, we performed multiple random samplings and 10-fold cross-validation to identify stable feature combinations. Specifically, we performed 1,000 iterations of the Wilcoxon rank sum test for differential analysis, each time using a randomly sampled subsets (90% of samples per iteration). We calculated the mean squared error values for each of 10 folds and averaged them to evaluate the overall performance of the different feature subsets.

3. Fig 4 g,h) This is unclear to me, but seems important. What is meant by “based on different combinations of cfDNA fragmentonics in the training set, validation set and test set”. Why is the dashed line drawn for the best test dataset (the mean/median seems potentially more appropriate)? Also, in the text, what is meant by “optimal model”? If this means that this is the maximum model performance over the different random dataset splits, this is not an acceptable metric to report. If, on the other hand, this relates to different sets of features, this needs to be explained in more detail.
Author response: Thank you for your insightful comments. We acknowledge that the original description in the figure and text was unclear, and we’ve made revisions to improve clarity. First, we have updated Fig. 4 g,h to Fig. 4 j,k. The bar plots show the mean AUC and sensitivity of PE models based on different combinations of cfDNA fragmentomics types. The error bar plot represent the mean ± standard deviation of AUCs or sensitivities for models based on different combinations of cfDNA fragmentomics over the same type number of cfDNA fragmentomics. Regarding the AUCs and sensitivities in Fig 4 j,k were the performance of models based on different sets of features. “cfDNA (1)”, “cfDNA (2)”, and “cfDNA (3)” correspond to one, two, and three types of cfDNA fragmentomics, respectively. For example, the “cfDNA (2)” bar plot shows the mean AUC of three models, each based on a different set of features: 1) 16 TSS coverages and 8 TSS scores, 2) 16 TSS coverages and 8 Gini coefficients, 3) 8 TSS scores and 8 Gini coefficients (Supplementary

Table 16). Regarding the term “optimal model”, we defined this as the model that achieved the highest performance on the validation dataset based on a specific feature set. Now, we also updated the figure legend and the relevant section of the “Results” to include this description in more detail.

4. “Furthermore, we observed an increase in AUC with increasing cfDNA fragmentomics.” This is unclear to me.
Author response: Thank you for your feedback. We agree that the original sentence was unclear, and we have revised it for better clarity. The revised sentence now is “When comparing the performances of models based on different combinations of cfDNA fragmentomics, we observed a progressive increase in the area under the curve (AUC) as the number of cfDNA fragmentomics included in the model for prediction early-onset PE increased.” The graphical representation of this sentence is shown in Fig. 4j, where the height of the bar plot increases as more cfDNA fragmentomic features are included into the model.

5. A false positive rate of 9% may seem like a low number to many readers, but if this method is intended for screening applications, this may be too high given the relatively low prevalence. This should be discussed. For instance, are follow-up tests available for cases with positive test results?
Author response: Thank you for raising this important point. We did consider the issue of false positive rates before. We focused on two approaches for selecting sensitivity and specificity: (1) the value where the sum of sensitivity and specificity is maximized, and (2) the sensitivity at a fixed false positive rate (in this case, 9%). The first approach can be challenging to compare other studies, as different articles reported different values for the sensitivity and specificity. Additionally, many studies on PE screening adopt a false positive rate of 10% to calculate sensitivity, which makes it easier to compare results across studies. For these reasons, we chose to use the second approach, focusing on the sensitivity at a 10% false positive rate. In fact, for this study, the sensitivity at a 9% false positive rate is very similar to that at 10%, so we chose to report the 9% false positive result. However, we also have the results for other false positive thresholds, though we did not present them all in the paper due to the large volume of tables generated from the 1000 bootstrap iterations.

Data availability: Raw data should be made available via restricted access databases, and anonymized mapped .bam files and downstream data should be made publicly available.
Author response: Thank you for your suggestion. To ensure data accessibility while respecting ethical and privacy regulations, the unprocessed cfDNA fragmentomic feature data has been made available through the Genome Sequence Archive (GSA) (Project ID: PRJCA035151). However, the raw sequencing data cannot be shared due to privacy restrictions. The key data supporting our findings are included within the article and its supplementary materials. These descriptions have also added in the “Data availability” section.

Code availability: Analysis source code should be made available to the reviewers (and later publicly).
Author response: Thank you for your suggestion. The analysis source code has been uploaded to

GitHub and is now available at the following link: https://github.com/wenqiuxu/cfDNA-fragmentomics

Data and Code Availability:** We confirm that our raw sequencing data and analysis code will be made available through appropriate repositories.

Previous literature regarding the use of cfDNA for PE prediction (especially those using cfRNA and cfDNAme) is insufficiently discussed. There is a performance comparison to the method from De Borre et al., however the authors do not mention the difference in sample collection time. Samples in De Borre et al. were collected 9-14 weeks of gestation, whereas this study deals with samples collected at 12-26 weeks gestation. Indeed, the De Borre et al. study mentions that collection time has a major impact on performance (“Consequently, samples obtained from week 12 onwards showed a better performance, with an AUC of 0.792 versus 0.629 for samples obtained before week 12”). Therefore, it seems this comparison is not entirely fair.
Author response: Thank you for your thoughtful feedback. We acknowledge that our initial comparison with the study by De Borre et al. did not adequately address the differences in sample collection time, and we appreciate your highlighting this issue. To address this concern, we have made two key revisions to our paper: 1) Validation for Early Gestational Samples: While our initial analysis focused on samples collected between 12 and 26 weeks of gestation, we have now also validated the predictive capability of cfDNA fragmentomics for samples collected at or before 16 weeks. Importantly, only slight declines in performance were observed for both early-onset and late-onset PE models for samples collected at or before 16 weeks. For example, the early-onset PE model for samples collected at or before 16 weeks yielded AUCs of 0.920 (95% CI: 0.918-0.922), 0.835 (95% CI: 0.832-0.838), and 0.834 (95% CI: 0.830-0.839), with sensitivities of 0.651 (95% CI: 0.640-0.663), 0.552 (95% CI: 0.545-0.560), and 0.574 (95% CI: 0.563-0.585) at a 9% false positive rate across validation and test sets (Fig. 5d). These results are comparable to those seen with cfRNA and cfDNA methylation (cfDNAme), which further strengthens the robustness of our findings. 2) Internal features Comparison: We have also emphasized comparisons within our dataset, focusing on the performance of models that combine cfDNA fragmentomics with maternal risk factors (MFs) versus those that based on either cfDNA fragmentomics or MFs alone. These combined models demonstrated significantly improved performance, highlighting the value of integrating both cfDNA fragmentomics and maternal risk factors for PE prediction.

Cross-center analysis: It is good that the authors mention as a limitation of the study that cross-center comparison is not possible, but it is unfortunate that such a comparison is not possible.
Author response: Thank you for your comments. We acknowledge that cross-center comparison is an important aspect and appreciate your understanding of this limitation in our initial submission. To enhance the robustness and multi-center applicability of our findings, we have now revised our study design. In the revised analysis, we increased the number of samples and allocated data from different hospitals into separate datasets. Specifically, we designated data from two hospitals as completely independent test sets, ensuring the independence of each center and validating the generalizability of our models. We believe this improvement strengthens the overall robustness of

our findings.

Fragmentomics methods: The authors use the Gini coefficient to measure the fragment length diversity. Why did the authors not follow the PFE method described by Esfahani et al? It includes a normalization step that may be important and may therefore lead to improved performance. Also, line 108: This is phrased as if it were a novel concept, but it is not - Esfahani et al should be cited here. In general, the fragmentomics metrics used here are rather simplistic, and it could (optionally) be interesting to explore the use of other methods/tools for fragmentomics analysis (e.g. see those explored in Esfahani et al., or other tools used in oncology).

Author response: Thank you for your valuable feedback and for pointing out the use of the PFE method described by Esfahani et al. We initially attempted to use the full PFE method for data analysis, but we encountered significant challenges. The computational time required for processing each sample exceeded one week, which placed a heavy demand on our resources and overall study. As a result, we built our own script based on feature principles. Regarding normalization, we explored different standardization approaches before and chose to correct for sequencing depth using model fitting. We acknowledge that our analysis focuses on a more limited fragmentomics metrics compared to the PFE method. The current study emphasizes features related to transcription start sites (TSS), as these are more directly interpretable in terms of gene associations and their potential relevance to disease. However, we have intended to explore additional types of cfDNA fragmentomics, including those mentioned in your minor revision comment. While we initially determined that a final read depth of around 10x is adequate, we sequenced the samples to read depths of 20-40X, as limiting the analysis to 10X might result in the loss of some genetic information. We would continue to explore other cfDNA fragmentomics features and their potential relationship to preeclampsia, and we have added this limitation in the "Discussion" section.

Minor:

There are several grammar and spelling mistakes. The authors should go through the manuscript carefully to remove those. Also the axis legends etc. need to be polished.

The chosen read depth is confusing. The authors show that read depths around 10x are optimal, but then sequenced the samples to significantly higher depths (20-40x). Why? It should be made clear that the reported performance values (ROC-AUC etc) were reported for more deeply sequenced samples.

Author response: Thank you for your insightful question. This is an important consideration that other reviewers have also raised. In our initial evaluation, we determined that a minimum coverage of 10X was required to obtain stable and reliable results for cfDNA fragmentomic analysis. This 10X depth serves as a lower threshold, ensuring that key fragmentomic features are adequately detected.

However, we chose to sequence the larger cohort at 20-40X WGS coverage based on the following consideration. These plasma cfDNA samples are highly valuable, and we did not want to limit their use solely to fragmentomic analysis. Higher-depth sequencing enables broader genomic investigations, including genetic studies that require greater coverage to detect rare variants,

structural variations, or other genetic features that may not be reliably identified at lower depths. While 10X depth was determined as the minimum requirement for fragmentomics analysis, we also considered the long-term research potential of deeper sequencing data. Given the costs associated with sequencing and the possibility of future analyses beyond cfDNA fragmentomics, we decided that sequencing at 20-40X would be a cost-effective strategy while enhancing the comprehensiveness and versatility of the dataset.

Fig 3: To me, the illustration suggested that the analysis was done 1000 * 1000 times, but according to the methods only the sampling is repeated 1000 times. Consider removing the second “repeat 1000 times” annotation under the arrow.

Author response: Thank you very much for your careful review and helpful suggestion regarding Fig. 3. We truly appreciate your attention to detail, which has helped us improve the clarity of our illustration. We agree to remove the second "repeat 1000 times" annotation under the arrow and have revised the figure.

Fig 1: In general, the clarity of this figure could be improved, and it could be made more concise.

f) What is meant by “gene abundances”?

g) “d” as y axis legend is unclear, and I find this plot hard to follow

n) Dots are too large and overlap, so hard to assess.

Author response: Thank you for your valuable feedback. We have made the following revisions to improve the clarity and conciseness of Figure 1.

Fig.1f: The term “gene abundances” referred to the results of gene expression obtained from microarray analysis. To enhance clarity, we have revised this term to “the expression levels” and provided further explained in the figure legend.

Fig.1g: The left y-axis label represented the 95% confidence intervals (CIs) of cfDNA fragmentomic values within each gene group. We recognize that the abbreviation “ci” may have been unclear, so we have updated the label to explicitly state “95% confidence intervals (CIs).”

Fig.1n: We acknowledge that the dot size made it difficult to assess individual data points. To improve visibility, we have adjusted the dot size and transformed the y-axis values into a power scale for better readability.

Methods: This sentence is unclear to me: “TSS regions of transcripts with the same TSS were merged, and redundancy removed”.

Author response: Thank you for your comment. We apologize for the lack of clarity in this sentence. To clarify, transcripts sharing the same transcription start site (TSS) were merged to eliminate redundancy, ensuring that each TSS region was represented only once in the analysis. We also have revised the sentence in the “Methods” section.

Introduction, line 68: Consider rephrasing.

Author response: Thank you for your comment. We have revised this sentence.

Reviewer #3 (Remarks to the Author):

In the current manuscript the authors explore the utility of "cfDNA fragmentomics" = several cfDNA fragmentomic features for preeclampsia (PE) risk assessment. The similar approach of so called "cfDNA promotor profiling" has been previously explored by Guo et al., 2020 (PMID: 32274292) for detection of different pregnancy complications (including preeclampsia), or for foetal growth restriction (Xu et al., 2021, PMID: 32364311). In the listed publications, as well in the current study, the comparison of coverage profiles in the promoter areas (around the transcriptions start sites, TSS) between cases and controls are used for identification of differential read depths potentially associated with the differential gene expression in the tissues of origin, which are then used to build classifiers to predict pregnancy complications. In the current study, in addition to TSS coverage, the authors also bring TSS score, which corresponds to the read distribution within the TSS region (Chen et al., 2021) and Gini coefficient, relating to the diversity of the cfDNA fragment lengths. If previous studies were performed on low-pass sequencing data (0.3X) generated routinely during NIPT (and, f.e, demonstrated sensitivity of 81.7% at AUC=0.825 (0.770-0.880) for PE prediction in a study by Guo et al., 2020), then in the current study the authors suggest that an average coverage of 10X is required for the proposed fragmentomic analyses. As a result, by using genome-wide cfDNA data sequenced 20-40X from healthy pregnant and cases with early- and late-onset PE, the authors by creating the combined fragmentomic and clinical (maternal factor) feature-built models achieved the "mean sensitivities of 73.9% (area under the curve (AUC)=0.92)) for early-onset and 58.7% (AUC=0.83) for late-onset PE prediction", respectively.
After reading the results section, it is then clear that the model built using only maternal factors (MF) performs better than fragmentomic-based, and addition of the fragmentomic features slightly improves the model characteristics for PE prediction. The main driving factor based on maternal characteristics might be associated with the distinct clinical characteristics of the cases and control groups? Although both cohorts have the matched gestational age, then the maternal age, BMI, and mean arterial pressure are significantly higher in the PE group, which might drive machine learning MF-based predictions? Also, this might be the biological reason why the predictive power for training vs validation sets is so different for fragmentomic approaches and do not generalize across testing sets, as in pregnant with high BMI, the foetal fraction is normally lower than in healthy pregnancies, moreover, high arterial pressure also affects cfDNA levels, which might hinder or affect the analysis? What do authors think about it?
Author response: Thank you very much for your thoughtful and insightful feedback. We appreciate your detailed observations regarding the performance of the models based on maternal factors (MF) versus cfDNA fragmentomics features. We acknowledge that, statistically, the model based on maternal factors (MF) and cfDNA fragmentomics provides only a modest significant improvement in the early-onset preeclampsia (PE) prediction when compared to the model based on maternal factors (MF) or cfDNA fragmentomics alone. However, we observed a more significant improvement in the late-onset PE model when fragmentomic features were incorporated. We believe that the limited statistical significance observed in the early-onset PE model may be due to the smaller sample size, which could have impacted the robustness of the results. To address this

limitation, we have increased the sample size and revised our study design to enhance the robustness and generalizability of our findings (Fig.2). Specifically, we have now divided the datasets by individual hospital to ensure the independence of hospital datasets, which will also strengthen the multi-center applicability of our results. Additionally, to ensure more accurate validation of the PE model performance, we have now designated data from two additional hospitals as two completely independent test sets, which were not involved in the feature selection process.

We also agree with your observation that maternal characteristics—such as age, BMI, and mean arterial pressure—play a crucial role in the model's predictive power. In our study, pregnant women with PE were carefully selected from four hospitals based on strict clinical criteria, while control samples were randomly selected and matched for gestational age. This approach ensures that the differences in maternal factors between the PE and control groups were not artificially induced. However, as you rightly pointed out, elevated maternal age, BMI, and blood pressure are well-established risk factors for PE, and these factors may indeed be driving the maternal factor-based predictions in our models. We also recognize that these maternal factors, particularly high BMI and elevated blood pressure, can affect cfDNA levels, including the fetal fraction. In pregnancies with higher BMI, for example, the fetal fraction tends to be lower, which could affect the accuracy of cfDNA-based models. These factors suggest, on the one hand, that finding stable cfDNA fragmentomics features is necessary. On the other hand, we believe that both cfDNA fragmentomics and maternal clinical risk factors are important and should not be overlooked. While we acknowledge our current sample size may not be sufficient for large-scale screening, we believe the findings still offer valuable insights for clinical practice, particularly in the Chinese population. We have also added this description into the third paragraph of "Discussion" section.

The important question, what is the added value of the proposed "cfDNA fragmentomics", which requires 10X depth sequencing and how the authors foresee implementation of such analysis in the clinical routine, considering that such deep sequencing is not routinely used for NIPT?

Author response: Thank you for your question. We understand that the proposed cfDNA fragmentomics analysis, which requires a 10X sequencing depth at least, might raise concerns regarding its clinical feasibility, particularly considering that such deep sequencing is not routinely used for NIPT. In any sequencing experiment, the depth (or the number of reads generated) plays a crucial role in determining how comprehensively the genomic or transcriptomic information is captured. Adequate sequencing depth is essential to achieving saturation. This is particularly important for analyzing low-abundance features, rare variants, or subtle differences in expression-issues that are often encountered in studies like cfDNA fragmentomics or RNAseq. In the context of RNAseq, having a saturating sequencing depth is critical for: 1) Detecting low-abundance features such as rare transcripts. 2) Improving the accuracy and reproducibility of gene expression measurements. 3) Avoiding sampling biases, which ensures that the analysis is more representative of the true biological state.

For cfDNA fragmentomics, the principle is similar. If the sequencing depth is inadequate, it could result in an incomplete or biased view of cfDNA levels, particularly in regions of interest, like the transcription start sites (TSS). This would be like to random sampling, where not enough of the

relevant cfDNA fragments are captured, potentially leading to inaccurate or unreliable downstream analyses, such as differential expression analysis or the predictive power of our models. A 10X sequencing depth ensures robust and accurate coverage of the cfDNA fragments, allowing for more reliable predictions, especially when analyzing subtle or low-abundance fragmentomic features.

We recognize that such deep sequencing is not currently routine in NIPT, where lower sequencing depths (e.g., 0.3X) are typically used. However, our approach is aimed at achieving a higher level of precision and robustness for specific applications like PE prediction, where the detection of subtle differences in cfDNA fragment patterns could significantly improve the predictive power and clinical utility of the model.

In the future, we foresee that as sequencing technologies continue to advance, and as the clinical utility of cfDNA fragmentomics becomes clearer, the integration of such deep sequencing could become more feasible. Additionally, reductions in deep sequencing costs could make such deep sequencing more accessible, supporting its integration into routine clinical workflows. We also anticipate that combining cfDNA fragmentomics with other clinical diagnostics—such as genetic disease detection—would maximize the added value of deep sequencing, particularly for high-risk pregnancies or specific conditions.

In general, the paper is vaguely written, and sometimes it’s difficult to follow the flow and interpretation of the results. Abstract would require additional information to introduce the theory of coverage and gene expression behind to the reader and additional explanation on the approaches used in the study are required. For example, the authors refer to “all three types of cfDNA fragmentomics”, but what metrics they refer to are unclear. The introduction section also requires reworking. The intro in the result section is better fit in the introduction section, as after reading the introduction the reader still has no clear picture on the fragmentomic theory behind. Figure legends also should be adapted to make it more clear to the reader to understand the results and the meaning of each plot (check also wording on the plots). Description of early and late PE could also go to introduction section from discussion art.

Author response: Thank you very much for your thoughtful feedback. We’ve made several revisions to enhance the overall readability. Specifically, we’ve moved the introductory material from the "Results" section, as well as the description of early-onset and late-onset PE from the "Discussion" section, into the "Introduction" section. This should provide readers with a clearer understanding of the background theory in the "Introduction" section. We’ve also updated figure legends, ensuring they provide better explanations of the results. Additionally, we’ve updated the abstract to introduce three types of cfDNA fragmentomics.

The paper describes 3 cfDNA fragmentomic features located in/around TSS sites. What is the overlap between TSS regions by using 3 different cfDNA fragmentomic features? If the same genes are differently regulated in PE-affected pregnancies, there should be overlap in TSS regions identified using different fragmentomic features? If not, what could be the reason for it?

Author response: Thank you for your thoughtful question. The original list of TSS regions analyzed

across the three cfDNA fragmentomic features was indeed the same and included all the TSS sites recorded in the NCBI database. However, after performing differential analysis and 10-fold cross-validation to select more important features for our predictive model, the final set of TSS regions corresponding to the three cfDNA fragmentomic features did not overlap. We acknowledge that this lack of overlap is an intriguing point, and we have now discussed this in the fourth paragraph of the "Discussion" section. In previous, when we explored the relationship between the cfDNA fragment levels at TSS regions and gene expression, we observed that not all patterns met our initial hypotheses. For example, elevated cfDNA signals at the TSS region of the *EGFR* gene in PE pregnancies were consistent with increased *EGFR* mRNA expression in preeclamptic placentas. We hypothesize that several biological mechanisms may influence the relationship between cfDNA coverage at TSS regions in plasma and gene expression in cells: 1) transcription factors (TFs) or other binding factors could occupy the TSS regions of highly expressed genes, which displace nucleosomes and may increase the abundance of shorter cfDNA fragments in plasma. Moreover, the concentration of TF binding has been shown to impact gene expression. 2) The increased placental autophagy and apoptosis rates in PE pregnancies could result in higher cell death and the release of more cfDNA fragments, including those from TSS regions of tissue-specific genes. This complex interplay of biological mechanisms—nucleosome occupancy, TF binding, cell death, and cfDNA degradation—could all contribute to variations in cfDNA fragment levels at TSS regions, further complicating the relationship between cfDNA fragment abundance and gene expression.

Thus, we believe that the non-overlapping TSS regions identified using different fragmentomic features reflect the fact that cfDNA fragmentomics captures a combined result of these diverse biological processes, and not just the gene expression levels alone. This further emphasizes the importance of adequate sequencing depth to ensure that these biological mechanisms are captured comprehensively and accurately.

Also, although the authors acknowledge that by using the secondary selection of the regions by selection only PE-specific once (database search) and top-ranked once might be a limitation, did the authors also evaluate the performance of the predictive models using the initial list of the differential TSS sites from the discovery phase?

Author response: Thank you for your question. We did evaluate the performance of the predictive models using the initial list of differential TSS sites, and we found that the performance was lower compared to the models based on the PE-specific selection (database search) and top-ranked features. Specifically, the early-onset PE model based on all differential TSS coverages, TSS scores, or Gini coefficients yielded AUCs of 0.7046 and 0.7635, 0.6636 and 0.6154, 0.7150 and 0.7434 in test sets, whereas the model based on selected features (PE-specific and top-ranked) achieved AUCs of 0.7848 and 0.7359, 0.7195 and 0.6817, 0.7415 and 0.7824. Similarly, for the late-onset PE model, the performance based on all differential TSS coverages, or Gini coefficients resulted in AUCs of 0.6853 and 0.6708, 0.7385 and 0.7429 in test sets, whereas the model based on the selected features performed better, with AUCs of 0.7392 and 0.7372, 0.7422 and 0.7443. These results suggest that the feature selection approach we used significantly improved model performance.

As a first step, the authors "determine a reasonable depth for cfDNA WGS" by sequencing 50X the cfDNA from healthy pregnant to evaluate the required for the analysis sequencing depth. They estimate that the 10X coverage would be required for the cfDNA fragmentomic analysis, however, then they still proceed with the higher coverage for other samples "Subsequently, we performed WGS (~20-40X) of plasma cfDNA from 476 healthy pregnant women, 109 pregnant women..". Is there any reason behind? Are these samples then down sampled to the 10X cov?

Author response: Thank you for your insightful question. This is an important consideration that other reviewers have also raised. In our initial evaluation, we determined that a minimum coverage of 10X was required to obtain stable and reliable results for cfDNA fragmentomic analysis. This 10X depth serves as a lower threshold, ensuring that key fragmentomic features are adequately detected.

However, we chose to sequence the larger cohort at 20-40X WGS coverage based on the following consideration. These plasma cfDNA samples are highly valuable, and we did not want to limit their use solely to fragmentomic analysis. Higher-depth sequencing enables broader genomic investigations, including genetic studies that require greater coverage to detect rare variants, structural variations, or other genetic features that may not be reliably identified at lower depths. While 10X depth was determined as the minimum requirement for fragmentomics analysis, we also considered the long-term research potential of deeper sequencing data. Given the costs associated with sequencing and the possibility of future analyses beyond cfDNA fragmentomics, we decided that sequencing at 20-40X would be a cost-effective strategy while enhancing the comprehensiveness and versatility of the dataset.

Minor remarks:

- Check the concordance of the presented results? E.g. in discussion:

At this threshold, the early- and late-onset PE models developed in this study based on MFs and cfDNA fragmentomics showed good performances for predicting early- and late-onset PE in the test set (AUCs: 0.923 and 0.828), achieving sensitivities of 68.7% and 58.4%, respectively, at a 9% false-positive rate. In abstract: With a false positive rate of 9%, the combined models exhibited mean sensitivities of 73.9% (area under the curve (AUC)=0.92) for early-onset and 58.7% (AUC=0.83)

Author response: Thank you for pointing this out. To ensure consistency between the abstract and the "Discussion" section, we have revised this description in the abstract and "Discussion" section.

- Figure 1 a-c is graphical representation of the study design (overview of the fragmentomic features used for classifiers), which could be combined with the Figure 2?

Author response: Thank you for your suggestion. We agree that combining Figure 1a–c, which provides an overview of the fragmentomic features, with Figure 2. We have also made its change in the manuscript.

- Typo: "Among these cfDNA fragnentomic approaches", and later on "fragmentonics" and its variation throughout the text?

Author response: Thank you for your comment. We sincerely apologize for the inconsistencies with the term “cfDNA fragmentomics.” We have corrected the spelling errors, including the use of “fragmentonics” and other variations throughout the manuscript, ensuring consistency in terminology.

- “Among these cfDNA fragnentomic approaches, both the promoter profiling of maternal plasma cfDNA and cfDNA concentration” – does concentration relate to fragmentomics approaches?

Author response: Thank you for pointing out the ambiguity. We did not use cfDNA concentration as one of cfDNA fragmentomic approach. We realized that this sentence was unclear. We have revised the description in the “Introduction” section to better clarify this point.

- “In plasma, cfDNA levels derived from active promoters in tissues or blood cells are lower than those from inactive promoters due to the presence or absence of nucleosome occupancies. However, this type of cfDNA fragmentomic has not been thoroughly explored for differences in cfDNA levels in plasma at various distances from the transcription start site (TSS).” – Isn’t the TSS depth coverage analysis is explored in ref 17? Or what authors refer to?

Author response: Thank you for your comment. You are correct that TSS depth coverage analysis has been explored in reference 17. However, what we are referring to here is the TSS score, which is a distinct cfDNA fragmentomic metric. The TSS score specifically illustrates the distribution of cfDNA levels at various distances from the TSS (Fig. 2b, formula 2 in the "Methods" section). This particular approach has not been explored in reference 17.

- How the diversity of cfDNA fragment lengths is calculated? Its unclear from the text and not well described in the methods.

Author response: Thank you for your comment. The diversity of cfDNA fragment lengths is calculated using the Gini coefficient, which we implemented using the DescTools package. To make our approach clearer, we have now uploaded all the relevant code to GitHub, as suggested by other reviewers. We hope this makes the methodology more transparent.

Methods:

- The use of RNA-seq data for blood and placenta is poorly understood (also in result section). How do authors do the correlation analysis?

Author response: Thank you for your comment and for pointing out the need for clarification. The gene expression levels data for blood and placenta were downloaded from published articles. Based on suggestions from other reviewers and the editor, we used Spearman correlation to capture the relationship between the fragmentomics indices and gene expression. We’ve now provided more detailed information in the “Methods” section to explain how we performed the correlation analysis.

- Ligated with and “A” tail - what does it mean?

Author response: Thank you for your comment and for pointing this out. After end-repair, an

adenine (A) base is added to the 3' end of the DNA fragments. This "A" tail is essential because sequencing adapters typically have a complementary thymine (T) base, which allows for efficient ligation of the adapters to the cfDNA fragments. We now revised this description to “addition of an "A" tail”.

- Explain the samtools FLAG sets used for analysis better.

Author response: Thank you for your thoughtful question regarding our selection of SAM flags for cfDNA fragmentomics analysis. We specifically retained reads with SAM flags 81, 83, 97, 99, 145, 147, 161, and 163, as these correspond to properly paired, uniquely mapped, and high-quality read pairs in a paired-end sequencing dataset. This description have also been added in the “Methods” section.

Each of these flags represents reads that meet the following criteria:

1) Properly paired: The read and its mate are mapped to the same chromosome and within a reasonable insert size.

2) Mapped in a forward or reverse orientation: These flags ensure that both read 1 and read 2 are considered, maintaining the correct orientation for fragment size calculations.

3) Not secondary or supplementary alignments: This helps avoid redundant or ambiguous mappings.

4) Not unmapped: Ensures that only confidently mapped reads contribute to the analysis.

These criteria are essential for accurate cfDNA fragmentomics analysis, as they minimize noise from misaligned or low-quality reads while ensuring the precise measurement of fragment sizes.

REVIEWER COMMENTS

Reviewer #1 (Remarks to the Author):

The manuscript has been significantly improved according to the suggestions of the reviewers which were followed meticulously by the authors. This is appreciated as well as all the efforts made in this study. However, there are still several issues which require revisions before the paper can be accepted for publication:

1. One of the reviewers questioned why a 9% FPR was used and the authors reasoned that “many studies on PE screening adopt a false positive rate of 10% to calculate sensitivity, which makes it easier to compare results across studies. For these reasons, we chose to use the second approach, focusing on the sensitivity at a 10% false positive rate. In fact, for this study, the sensitivity at a 9% false positive rate is very similar to that at 10%, so we chose to report the 9% false”. However, it is not understandable why 9% was chosen and not 10% to enable direct comparisons with other studies. I do not suggest rewriting the whole paper but at least a good reason needs to be provided why exactly 9% was chosen.

Author response: We thank the reviewer for raising this important point. We fully agree that using a 10% false positive rate (FPR) is the standard in the field and facilitates direct comparison across studies. To address this, we have now **standardized all sensitivity results to correspond to a 10% false positive rate** throughout the manuscript. This includes updates to the Abstract, Results, Figures, and Supplementary Tables.

2. I pointed out earlier that a simple biochemical blood test (PlGF) combined with ultrasound and maternal risk factors provide better screening measures for PE than the presented very tedious and expensive method. In the meantime, a study was published in Nature Medicine showing the value of low coverage prenatal cfDNA WGS data for the quantification of maternal and fetal tissue signatures using nucleosome accessibility and its potential use for the very good prediction of PE before 16 weeks of gestation. The authors need to discuss their findings considering this newly published study, to compare the strengths and limitations of both methods and the real clinical utility of their methodology in the light of these parallel developments.

Author response: We thank the reviewer for these important comments. Now, we have addressed these points in the revised Discussion section. First, we discussed the Fetal Medicine Foundation (FMF) first trimester prediction model in detail **in the first paragraph of Discussion section**, as follows:

> “*The Fetal Medicine Foundation (FMF) first trimester prediction model based on maternal risk factors, mean arterial pressure, uterine artery pulsatility index (UtA-PI), and serum biomarkers such as PIGF has been externally validated in diverse prospective cohorts and is regarded as a reliable standard for early screening of PE*[29-33]*. However, its predictive performance varies across populations. At a false-positive rates of 10%, detection rates for preterm PE were reported at 56.4% in Chinese and*

64.0% in Asian populations[32-33]. Moreover, the application of UtA-PI, despite its potential, has not been widely adopted, particularly in China, due to its demanding and complex measurement technique." This is the reason why we want to explore other approaches to predict PE.

Second, We have carefully studied the recent publication by Adil, et al. (Nature Medicine, 2025, PMID 39939524), which also demonstrated the potential of low-coverage cfDNA WGS (~0.5X) for PE prediction through analysis of nucleosome positioning. We acknowledge the importance of this study and have now discussed **its findings in the second paragraph of the Discussion** as follows:

"*Although previous and recent studies have showed the feasibility of building PE prediction models using low-depth cfDNA WGS data (0.3X or 0.5X) (PMID: 32274292, 39939524), which leveraged existing clinical screenings without requiring additional sampling or sequencing, we observed variability in model performance across different validation datasets when using NIPT data (AUC: 0.898 versus 0.804 (PMID: 32274292), and AUC: 0.850 versus 0.920 (PMID: 39939524)). We hypothesized that this variability might be attributed to the instability of feature at low sequencing depths. Similarly, in our previous work using ultra-low-depth NIPT data (0.1X), originally intended for fetal aneuploidy screening, fluctuations in model performance were observed across three validation cohorts from three different hospitals (AUC: 0.790 versus 0.760 versus 0.600) (PMID: 38695016). Additionally, the NIPT data routinely used for fetal aneuploidy screening, whether 0.1X or 0.5X, are generated using single-end sequencing. Compared with paired-end sequencing data, single-end sequencing data cannot determine the fragment length of maternal plasma cfDNA, limiting the obtainable fragmentomic information.*" Therefore, we considered it necessary to explore the reasonable minimum sequencing depth required for relatively stable and reliable cfDNA fragmentomics analysis.

3. In Table 1, it would be better to define "parity" and "gravidity" instead of "birth history" and "pregnancy history" to better adhere with the literature.

Author response: Thank you for your comment. We have revised **Table 1 and Extended Data Table 1-3** to replace "birth history" with "parity" and "pregnancy history" with "gravidity", in accordance with standard obstetric terminology.

4. It is confusing that PE was defined by the onset of symptoms, however, Table 1 provides gestational ages at delivery, and EPE medians are almost higher than the 34-week cutoff for EPE. Therefore, I suggest providing gestational ages for both onset and delivery, as well.

Author response: Thank you for pointing this out. We have now added gestational ages at both onset and delivery in **Table 1 and Extended Data Table 1-3** to improve clarity. As suggested, we also clarified in the **Methods section** that classification of early-onset and late-onset preeclampsia was based on the gestational age at onset of symptoms, rather than the gestational age at delivery.

5. Starting from line 331 (especially between lines 400-450), overwhelming amounts of numbers are included into the text, which is thus very difficult to follow. This needs to be fundamentally changed.
Author response: We appreciate the reviewer's insightful comment. We agree that the high density of numerical values, particularly when including both 95% confidence intervals (CIs) and statistical significance levels (P values), may affect readability. However, considering that the 95% CIs for model performance metrics are critical for interpreting clinical utility and model stability, we have retained them in the main text. To address the reviewer's concern, we have carefully revised the affected sections, particularly **the second and third paragraphs of the Results section titled "CfDNA fragmentomics improves PE prediction."** For sections with large amounts of information, we have restructured the text by clearly separating descriptions for each gestational age group and model combination, and by using more concise and comparative language to summarize key findings. We hope these revisions improve clarity while preserving important quantitative detail.

Reviewer #2 (Remarks to the Author):

Several of my concerns were addressed, and the manuscript is clearer now. The multi-center evaluation is especially valuable.
I do still have major concerns especially about unfounded claims that the paper makes:
Major 1: The sequencing depth used in this study is still not presented adequately. In the methods section and in the response to reviewers, the authors mention that samples were sequenced to 20-40x, which I now assume is correct. However, in the abstract (line 49) and discussion (507), the authors write as if all their results were obtained at a threshold of 600 million reads (which they determined as optimal in initial experiments). This is strongly misleading, as it suggests that the classification performance they obtain comes from sequencing at 10x, while in reality it comes from sequencing at 20-40x. I see two options: Either completely rephrase to avoid making such claims, or perform a down-sampling analysis on all sequencing samples (randomly draw fragments to reach 10x coverage), and report all metrics on this down-sampled dataset. In either case, this should also be discussed in the Discussion.
Author response: We sincerely appreciate this important observation, which was also raised by other reviewers and editors. To ensure logical consistency and clarity, all analyses from the second Results section onward (including differential fragmentomic analysis, feature selection, and model construction and validation) were performed on data downsampled to ~10X coverage through reanalysis, as this was determined to be the reasonable minimum read depth required for relatively stable cfDNA fragmentomics analysis. We have now clearly stated this in **the Results section titled**

“CfDNA-fragmentomic features associated with PE” as follows:

"*As we established that a final read depth of at least 10X is required for relatively stable estimation of cfDNA fragmentomics, all data were downsampled to 10X to ensure consistency across the following analyses*".

In addition to evaluating the 10X-trained model on two test sets using the corresponding 10X-downsampled data, we further validated their performance on the original, pre-downsampled datasets. For pre-downsampled datasets, these two test sets were stratified by sequencing depth (10-30X and >30X), and yielded comparable or even improved predictive performance relative to the 10X-downsampled data, suggesting robustness across a range of cfDNA sequencing depths (**Extended Data Fig. 7, Supplementary Table 18-19**). These contents have been presented in the **Results section titled “Evaluation of cfDNA fragmentomics for PE prediction”** as follows:

“*Notably, the optimal models for early-onset PE and late-onset PE, developed using datasets downsampled to 10X coverage, also achieved comparable or even improved performance when evaluated on pre-downsampled samples from test sets, suggesting generalizability across read depths greater than 10X (Extended Data Fig. 7, Supplementary Table 18-19).*”

Major 2: Line 154: It is claimed: “Overall, these results indicated that at a sequencing depth of 600 million reads (~10X final read depth), TSS coverage, TSS score and Gini coefficient metrics could reliably discriminate genes with different expression levels in tissues and blood.”. This is vastly overstated. Even for the strongest and weakest-expressed genes, the boxes of the boxplots overlap. The performance is clearly worse than “reliable discrimination”, and this claim needs to be phrased much more conservatively.

Author response: We agree that the original statement was overstated. We have revised the sentence **in the second paragraph of Results section** to:

“*Overall, these results indicated that at a sequencing depth of 600 million reads (~10X final read depth), TSS coverage, TSS score, and Gini coefficient metrics showed modest but observable differences between genes with different expression levels in tissues and blood.*”

Additionally, we also clarified in the **Discussion-Limitations section** that ~10X final read depth does not represent the optimal depth for cfDNA fragmentomics analyses and predictive model development. The revised content as follows:

“*Third, while our analyses demonstrated that fragmentomic metrics, such as TSS coverage, TSS score, and Gini coefficient, showed statistically significant difference between gene expression categories at a read depth of ~10X, we acknowledged that this depth does not necessarily represent the optimal threshold for cfDNA fragmentomics analyses and predictive model development. Instead, the ~10X read depth might represent the reasonable minimum depth required for relatively stable estimation of cfDNA fragmentomic metrics, at which biologically informative signals could be extracted and used for risk prediction. Further studies leveraging a broader*

spectrum of sequencing depths and alternative modeling strategies might uncover more optimal analytical frameworks."

Major 3: The authors do not intend to share sequencing data due to privacy concerns. However, I do not see a reason not to share .bam file in anonymized form, such that fragmentomics information (start and end coordinates of each fragment) is provided, but the DNA sequence is replaced with the reference sequence. .bed files or similar of fragment start and end coordinates would also suffice.

Author response: We appreciate the reviewer's suggestion and fully understand the importance of data transparency. However, due to the large data volume (>100T raw data) and memory limitations during the initial analysis phase, we did not retain intermediate files such as .bam or .bed files containing only fragment start and end coordinates without sequence information. The raw sequencing data have been deposited in the CNGB Nucleotide Sequence Archive (CNSA, https://db.cngb.org/cnsa) under the accession number [accession number: CNP0005047]. Due to national regulations and participant privacy considerations, these data are hosted on a secure server and require controlled access. Researchers interested in accessing the raw data are encouraged to contact the corresponding authors with a brief description of their intended research. Access will be granted to qualified investigators upon approval and completion of a data access agreement, in accordance with the data-sharing policies approved by our institutional review board and data governance committee. We have updated the "Data Availability" section in the manuscript to reflect this. In addition, source data supporting each figure panel have been provided. All code and figure source data, including input and output files for each panel, also are available on Code Ocean (https://codeocean.com/capsule/7028212/tree).

- Line 391: "Notably, these results were comparable to or even better than the predictive performance of cfDNA methylation (cfDNAme)26, or cfRNA profiles27. " and especially line 512 "The performance of the early-onset PE model outperformed other omics-based approaches26,27. "- It is not adequately discussed that different sampling occurred during different gestational ages, which makes it hard to compare. In my opinion, "outperformed" is not justified here without further in-depth comparisons.

Author response: Thank you for the reviewer's insightful comment regarding the challenges of directly comparing model performance across studies with differing gestational ages at sample collection. To avoid overinterpretation in the Results section, we have removed the sentence: "*Notably, these results were comparable to or even better than the predictive performance of cfDNA methylation (cfDNAme)26, or cfRNA profiles27*." Detailed comparisons with other omics-based approaches have now been included **in the third paragraph of the Discussion section**, as follows:

"*Notably, our PE models based on cfDNA fragmentomics alone were better than other omics-based approaches*[26,37]*. For example, the early-onset PE model based on cfDNA methylation alone showed lower predictive performance for samples collected*

before 16 weeks of gestation (AUC: 0.752)[26], compared to our early-onset PE model based on cfDNA fragmentomics alone, which achieved a higher AUC of 0.852 for samples collected at the same gestational age in test sets. Likewise, both our cfDNA fragmentomics-based models for early-onset (AUC: 0.852) and late-onset PE (AUC: 0.789) outperformed the PE model based on cfRNA profiles alone, which reported AUCs of 0.710, 0.720, and 0.740 across validation cohorts for samples obtained at the same gestational age[37]."

- Code documentation and organization should be improved. For instance, I could not easily find the code for the machine learning procedures.

Author response: We thank the reviewer for this helpful comment. We acknowledge that the organization and accessibility of code could be further improved. Initially, due to file size limitations encountered when using GitHub, some components (particularly large input/output files and full machine learning workflows) could not be uploaded there. To address this, we have now made all code and figure source data, including scripts for machine learning procedures and the corresponding input/output files for each figure panel, publicly available on Code Ocean (https://codeocean.com/capsule/7028212/tree), which supports larger file sizes and offers a structured environment for reproducible research. We have also updated the "Code Availability" section in the manuscript accordingly.

Minor 1: The impact of a 9% false positive rate in clinical practice should be discussed

Author response: We thank the reviewer for raising this important point, which was also mentioned by other reviewers. We fully agree that using a 10% false positive rate (FPR) is the standard in the field and facilitates direct comparison across studies. To address this, we have now **standardized all sensitivity results to correspond to a 10% false positive rate** throughout the manuscript. This includes updates to the Abstract, Results, Figures, and Supplementary Tables. We also agree that this 10% FPR should be discussed. In **the third paragraph of the Discussion section**, we have added a brief discussion to address this point.

"*A 10% false positive rate might be relatively high, resulting in unnecessary anxiety and additional follow-up testing. However, this rate remains within the range observed for other clinically accepted prenatal screening tests, such as first-trimester combined screening for preeclampsia[30-33].*"

Minor 2: Line 102: Revise grammar

Author response: We thank the reviewer for the helpful suggestion. We have revised the sentence as follows:

"*To capture this variation, we used the Gini coefficient to quantify the diversity of cfDNA fragment lengths at each 2kb TSS region, reflecting the variability in fragment size distributions.*"

Minor 3: Table 1: What do the superscript values mean? This is not explained.

Author response: We thank the reviewer for pointing this out. In Table 1, the superscript values represented the gestational age (in days) at the time of sample collection, delivery, and onset of PE. We have now added explanations of these superscripts in the **Table 1 and Extended Data Table 1-3** to avoid confusion.

Minor 4: Fig 4 j-k: Real data points should be shown in addition to or instead of the error bars. This would allow assessment whether e.g. the best 2-featuretype model is equal to the 3-feature-tpye model

Author response: We thank the reviewer for the suggestion. We have now updated **Fig. 4j-k** and **Extended Data Fig.6j-k** to include individual data points for each model result, in addition to the error bars.

Minor 5: Line 430: Provide the performance values or improvement values

Author response: We thank the reviewer for the suggestion. We have now included the corresponding performance values and statistical significance results in the revised manuscript. Specifically, we added the following sentence in the **Results section titled "CfDNA fragmentomics improves PE prediction"**:

> "*Importantly, performance improvements of early-onset PE model were also observed in a subgroup of participants whose samples were collected before or at 16 weeks' gestation, although these improvements were not statistically significant ($P_{combined\ vs\ MFs}$=0.092, 0.630, and 0.237, and $P_{combined\ vs\ cfDNA\ fragmentomics}$=0.578, 0.030, and 0.157 in validation and test sets, respectively). In this early-gestation subgroup, the combined model achieved AUCs of 0.902 (95% CI: 0.898-0.905), 0.900 (95% CI: 0.898-0.902), and 0.893 (95% CI: 0.890-0.896) in the validation and test sets, respectively.*"

Reviewer #2 (Remarks on code availability):

Code documentation and organization should be improved. For instance, I could not find the code for the machine learning procedures. The workflow is not explained and structured well enough to easily reproduce the results.

E.g. Which input files are needed?

Also, the markdown is not rendered properly on Github

Author response: We thank the reviewer for this helpful comment. We acknowledge that the organization and accessibility of code could be further improved. Initially, due to file size limitations encountered when using GitHub, some components (particularly large input/output files and full machine learning workflows) could not be uploaded there. To address this, we have now made all code and figure source data, including scripts for machine learning procedures and the corresponding input/output files for each figure panel, publicly available on Code Ocean

(https://codeocean.com/capsule/7028212/tree), which supports larger file sizes and offers a structured environment for reproducible research. We have also updated the “Code Availability” section in the manuscript accordingly.

Reviewer #4 (Remarks to the Author):

Thank you for the opportunity to review your paper. I was asked primarily to comment on whether Reviewer 3's comments were addressed.

COMMENT 1-1: Regarding performance models, impact of maternal factors, etc.
I appreciate the authors attention to responding to this critique, including adding additional cases and sites. Their results do suggest that their approach to incorporate cfDNA fragmentomics improves preeclampsia prediction above maternal risk factors alone. I have a few remaining questions / concerns:
• When were the clinical parameters such as BMI and MAP assessed?
Author response: We thank the reviewer for this observation. To consistent with the cfDNA fragmentomics analyses, clinical parameters such as BMI and MAP measured at the same gestational age as maternal plasma sampling were collected. We acknowledged that this detail was not clearly described in the original submission. Accordingly, we have added this description in the **Online Methods titled *“Clinical risk factors analysis”* section** to clarify this point for improved transparency.

COMMENT 1-2: Fig 4 and Fig 5, you report AUCs of 1.0 for training, with subsequently substantially lower AUCs for validation and test sets. This is a red flag for model overfitting, suggesting that the model has memorized the training data, instead of learning generalizable patterns. This is likely to be an issue with assessment on a broader population (i.e., less “clean” control group). How do the authors explain this?
Author response: We appreciate the reviewer’s insightful comment regarding the potential overfitting indicated by the AUC of 1.0 in the training set. To mitigate overfitting and ensure generalizability of the model, we implemented several strategies during model development:
1) Combined Training and Validation for Parameter Tuning:
To avoid overly optimistic performance on the training set, we performed model selection and hyperparameter tuning using a combined training and validation set (X_cross, y_cross). The final model was then independently evaluated on two test sets.
2) Stratified Cross-Validation:
During the grid search (GridSearchCV), we employed stratified 5-fold cross-validation (StratifiedKFold(n_splits=5, shuffle=True, random_state=42)) to ensure balanced distribution of outcome classes within each fold, thus reducing sampling bias and enhancing robustness.
3) Regularization via min_samples_leaf:

We carefully tuned the min_samples_leaf hyperparameter of the Random Forest classifier, incrementally increasing it to values such as 20, 30, 40, and 50. This approach limits the complexity of the model by requiring a minimum number of samples per leaf, thereby helping to reduce the risk of overfitting.

Through these efforts, we were able to **avoid the occurrence of an AUC of 1.0** in the training set (Fig. 4, Fig. 5, Extended Data Fig. 6, Supplementary Table 16-17). However, we acknowledge that a degree of overfitting may still exist, which is a recognized limitation when working with relatively small or highly curated datasets. We have now **added a discussion of this limitation in the revised Discussion section:**

> "*Finally, although we adopted several strategies to reduce model overfitting, such as stratified cross-validation during parameter tuning and setting larger values for the 'min_samples_leaf' parameter in the random forest classifier, we acknowledged that a degree of overfitting might still exist. Such overfitting is a known limitation when using relatively small or highly curated datasets. Future prospective studies in larger and more diverse populations were needed to further assess the generalizability of our predictive models.*"

COMMENT 1-3: With respect to the "gestational-age matched controls" what were the outcomes of this control group? Did they only include those pregnancies ending in term delivery without hypertensive complications? If so, this would make the control group "cleaner" than what you would expect in a real population (i.e., if they excluded pregnancies ending in preterm birth for other reasons, or complicated by other pregnancy outcomes).

Author response: We thank the reviewer for this important comment. The gestational-age matched control group consisted of pregnant women who did not have any hypertensive disorders, other pregnancy complications, chromosomal abnormalities, malignancies, or severe cardiac, hepatic, or renal insufficiency. This description was included in the **Online methods "Study overview and Participants" section.** We acknowledged that this definition resulted in a relatively "clean" control group compared to what may be encountered in real-world populations. However, given that this was a retrospective study aiming to explore early signals of PE from cfDNA fragmentomics, we sought to minimize potential confounding by excluding other maternal or fetal complications. We have now **added this point as a limitation in the revised Discussion section:**

> "*First, as a retrospective study, the samples in our research might not fully represent the overall population. In particular, the control group was defined to exclude not only hypertensive disorders but also other pregnancy complications and maternal comorbidities, resulting in a cleaner cohort than might be expected in real-world settings. This approach was chosen to minimize potential confounding factors in this exploratory phase, but it might limit the generalizability of our findings to broader clinical populations.*"

COMMENT 1-4: Fig 4j-k: I found the explanation about what cfDNA(1), (2), and (3) mean still to be confusing. The text suggests that cfDNA(2) includes “two types of cfDNA fragmentomics”, but then your further example includes 3 different combinations of cfDNA fragmentomic features (TSS coverage, TSS scores, Gini coefficients). This needs to be clarified.

Author response: We thank the reviewer for this important comment. We agree that our previous explanation may have caused confusion between the number of cfDNA fragmentomic types used in a model and the number of different feature combinations. To address this, we have revised the corresponding **Figure legend (Fig. 4j-k and Extended Data Fig.6j-k)** in the manuscript:

> *“The error bar plots in j-k represent the mean ± standard deviation of AUCs or sensitivities for models constructed using combinations of one, two, or all three types of cfDNA fragmentomics. Specifically, “cfDNA (1)”, “cfDNA (2)”, and “cfDNA (3)” indicate models that incorporate one, two, and three fragmentomics types, respectively. For example, the “cfDNA (2)” group includes three models built with two types of cfDNA fragmentomics: 1) 16 TSS coverages and 14 TSS scores, 2) 16 TSS coverages and 16 Gini coefficients, 3) 14 TSS scores and 16 Gini coefficients. Similarly, “cfDNA (1)” includes models using each individual fragmentomic type separately, and “cfDNA (3)” refers to models that combine all three types. The detailed performance metrics of each model are provided in Supplementary Table 16 ”*

COMMENT 2: Concerns regarding need for 10X depth when this is not routinely performed during NIPT.

• I appreciate the importance of sequencing depth, however, a recent publication also utilized cfDNA fragmentomic analysis and these authors used low-coverage whole genome sequencing data (Adil, et al, Nature Medicine, 2025, PMID 39939524) at around 0.5X that also evaluated preeclampsia prediction. This study also included a less “clean” control population. I recommend the authors explain how the reader should position their manuscript in light of this recent publication. What is their approach adding in light of others showing similar results with less sequencing depth and a more "real world" control population?

Author response: We thank the reviewer for this important comment. We have carefully studied the recent publication by Adil, et al. (Nature Medicine, 2025, PMID 39939524), which also utilized cfDNA fragmentomic analysis based on low-coverage whole genome sequencing (~0.5X). We appreciated this research. Although previous and recent studies have demonstrated the feasibility of building PE prediction models using low-depth cfDNA WGS data (0.3X or 0.5X) (PMID: 32274292, 39939524), which leveraged existing clinical screenings without requiring additional sampling or sequencing, we observed variability in model performance across different validation datasets when using NIPT data (AUC: 0.898 versus 0.804 (PMID: 32274292), and AUC: 0.850 versus 0.920 (PMID: 39939524)). **We hypothesized that this variability might be attributed to the instability of feature at low sequencing depths.** Similarly, in our

previous work using ultra-low-depth NIPT data (0.1X), originally intended for fetal aneuploidy screening, fluctuations in model performance were observed across three validation cohorts from three different hospitals (AUC: 0.790 versus 0.760 versus 0.600) (PMID: 38695016). Additionally, the NIPT data routinely used for fetal aneuploidy screening, whether 0.1X or 0.5X, are generated using single-end sequencing. Compared with paired-end sequencing data, single-end sequencing data cannot determine the fragment length of maternal plasma cfDNA, limiting the obtainable fragmentomic information. Therefore, we considered it necessary to explore the reasonable minimum sequencing depth required for relatively stable and reliable cfDNA fragmentomics analysis. These relative descriptions have been added to **the second paragraph of the Discussion section.**

Regarding the control population, as discussed in our responses to previous comments (COMMENT 1-3), we acknowledge that our control group was relatively "clean" compared to what might be encountered in real-world populations. However, given that this was a retrospective study aiming to explore early signals of PE from cfDNA fragmentomics, we sought to minimize potential confounding by excluding other maternal or fetal complications. We have now **added this point as a limitation in the revised Discussion section:**

> *"First, as a retrospective study, the samples in our research might not fully represent the overall population. In particular, the control group was defined to exclude not only hypertensive disorders but also other pregnancy complications and maternal comorbidities, resulting in a cleaner cohort than might be expected in real-world settings. This approach was chosen to minimize potential confounding factors in this exploratory phase, but it might limit the generalizability of our findings to broader clinical populations."*

COMMENT 3: Regarding readability of the paper.

• I did not read the prior versions, but the readability of this manuscript is appropriate – however, see comment regarding confusion with Fig 4 legend. I do recommend changing the icon used to represent pregnant individuals throughout the Figures. The current icon is not pictorially representative of pregnant people and may be considered offensive.

Author response: We thank the reviewer for these comments. We have revised the Figure legends for **Fig. 4j-k and Extended Data Fig. 6j-k** to improve clarity regarding the combinations of cfDNA fragmentomic types used in each model. Additionally, we appreciate the reviewer's suggestion regarding the icon used to represent pregnant individuals in the Figures. We have now updated the icon throughout the manuscript to ensure it is more appropriate and inclusive.

COMMENT 4: Regarding overlap between TSS regions by using 3 different cfDNA fragmentomic features.

• No additional comments here.

COMMENT 5: Regarding evaluation of performance of the predictive models using the initial list of differential TSS sites from the discovery phase.
• No additional comments here.

COMMENT 6: Regarding determination of a "reasonable depth for cfDNA WGS" by sequencing 50X which lead to determining that 10X was sufficient, but then ultimately using 20-40X for their study.
• I agree with the prior reviewer about these concerns and did not find that this was adequately addressed. If you determined that 10X was sufficient, why then do you use 20-40X to develop and evaluate models for predicting PE (Line 625)? This goes back to prior points about translatability about your findings as 10-40X is not standard depth sequencing for NIPT. I appreciate that these are precious samples and you wanted to maximize their use – could you have considered downsampling? To me, this remains a major limitation of your approach and decreasing the innovation potential. Also, you should clearly state the median sequencing depth for each of your cohorts (training, validation, both test sets) as in the current set up, you are stating that 10X is sufficient, but that is not what you ultimately used for PE prediction.
Author response: We sincerely appreciate this important comment, which was also raised by other reviewers. To ensure logical consistency and clarity, now all analyses from the second Results section onward (including differential fragmentomic analysis, feature selection, and model construction and validation) were **performed on data downsampled to ~10X coverage through reanalysis**, as this was determined to be the reasonable minimum read depth required for relatively stable cfDNA fragmentomics analysis. We have now clearly stated this in **the Results section titled "CfDNA-fragmentomic features associated with PE"** as follows:

> "*As we established that a final read depth of at least 10X is required for relatively stable estimation of cfDNA fragmentomics, all data were downsampled to 10X to ensure consistency across the following analyses*".

The median sequencing depth for each cohort (training, validation, and both test sets) in the downsampled data is shown in **Extended Data Fig. 2a-c**. To further evaluate model robustness, we also tested the performance of models were trained on 10X-downsampled data on the original, pre-downsampled test sets, stratified by sequencing depth (10-30X and >30X). The sequencing depths for pre-downsampled test sets were included in the **(Extended Data Fig. 7, Supplementary Table 3)**. These analyses showed that model performance was comparable or even improved when applied to higher-depth samples (**Extended Data Fig. 7; Supplementary Tables 18-19**), supporting generalizability across a range of sequencing depths.

Next, we acknowledge that our earlier description may have overstated the sufficiency of 10X, and we have revised Abstract, Introduction, and Result sections to describe it as the "*reasonable minimum sequencing depth required for relatively stable and reliable cfDNA fragmentomics analysis*". It may not be the optimal depth for cfDNA

fragmentomics analyses and predictive model development. This limitation is now explicitly discussed in the **revised Discussion**, where we also note that further studies using broader depth ranges and alternative modeling strategies may help determine optimal conditions for clinical implementation.

COMMENT 7 (minor remark): Regarding concordance of presented figures.

• No additional comments here, except that I recommend you use a different icon to represent pregnant people.

Author response: We have now updated the icon throughout the manuscript to ensure it is more appropriate and inclusive.

COMMENT 8 (minor remark): Regarding Figure 1a-c

• No additional comments here, except that I recommend you use a different icon to represent pregnant people.

Author response: We have now updated the icon throughout the manuscript to ensure it is more appropriate and inclusive.

COMMENT 9 (minor remark): regarding typo

• No additional comments.

COMMENT 10 (minor remark): Regarding concentration.

• No additional comments.

COMMENT 11 (minor remark): Regarding TSS depth coverage

• Figure 2 a-c, for the x-axis for your bar plots, why is there is an arrow? You are looking at two distinct groups – TSS regions of active vs. inactive genes. What is the difference between Fig 2a and 2b? The x and y-axes are the same and the plots of the red and blue lines also look the same. What is the yellow shading and the green shading? Please explain this better in the figure legend.

Author response: We appreciate the reviewer's careful reading and valuable suggestions. we have removed the arrow from the x-axis in the bar plots of Figure 2a-c to avoid confusion, as the plots compare two distinct groups and do not represent a continuous variable. We have also separately clarified panels 2a and 2b: while both share the same axes and display read depth distributions around TSS regions, panel 2a presents overall TSS coverage at 2kb TSS regions of active (blue) and inactive genes (red), whereas panel 2b provides a schematic illustrating how the TSS score is calculated. Specifically,

> *"For TSS score calculation, each 2kb TSS region was divided into 20 bins of 100bp size. In panel* ***b****, the yellow and green shaded areas represent the 10 bins furthest from the TSS and 6 bins closest to the TSS, respectively. The TSS score for each TSS region was computed as the ratio of the mean read depth in the green bins to that in the yellow bins."*

We have revised the figure legend accordingly to improve clarity.

COMMENT 12 (minor remark): Diversity of cfDNA fragment lengths.

• I had a hard time following what the Gini coefficient was used for? My understanding is that this quantifies diversity of cfDNA fragments in a sample. How did this specific metric help with designing your classifier towards your ultimate goal (PE prediction)? Consider elaborating on this in the Discussion to explain why this was important. Also Line 102 is an incomplete sentence: "The Gini coefficient, which quantifies the size distribution of cfDNA fragments at transcription start sites (TSS)." This is why there is further confusion about what the Gini metric is serving in your study.

Author response: We thank the reviewer's question on the rationale and utility of the Gini coefficient in our study. The Gini coefficient was originally developed in the field of economics. Its primary purpose was to measure income inequality or wealth distribution within a population. Now, the Gini coefficient has been adapted for use in many other disciplines beyond economics. In your study, the Gini coefficient was used to quantify the diversity of cfDNA fragment lengths at each 2kb TSS region, reflecting the variability in fragment size distributions. We reasoned that cfDNA fragments at the TSS regions of active genes are more likely to undergo random cleavage, resulting in a broader and more heterogeneous fragment size distribution, while those at TSS regions of inactive genes tend to show less variation due to nucleosome occupancy, leading to more homogeneous fragment sizes. Therefore, the Gini coefficient serves as a useful metric to capture this difference in fragmentation diversity, which may reflect underlying biological states associated with placental dysfunction in preeclampsia (PE). We have revised these descriptions **in the Introduction section**.

To clarify this point, we have also discussed this content **in the Discussion section as follows:**

> *"In addition to fragment abundance, we also examined cfDNA fragment length diversity, quantified using the Gini coefficient at TSS regions. This metric might also reflect chromatin accessibility and cleavage dynamics that differ between active and inactive genomic regions. Given that PE is associated with dysregulated gene expression and abnormal cell death in placental or blood cells, the Gini coefficient might also capture these underlying biological changes. Indeed, TSS regions of LRP1B and TFDP2 genes exhibited more homogeneous fragment sizes in pregnant women with PE, consistent with their expression in placentas[46,47]. When combined with fragment coverage and other fragmentomics, it might offer additional predictive value for PE detection. These findings highlighted the multifaceted nature of cfDNA fragmentomics and the importance of integrating both abundance- and length diversity-based metrics to gain deeper insights into the pathophysiology of PE."*

In addition, we have corrected the incomplete sentence on Line 102. The revised sentence now reads:

> *"To capture this variation, we used the Gini coefficient to quantify the diversity of cfDNA fragment lengths at each 2kb TSS region, reflecting the variability in fragment*

size distributions."

COMMMENT 13 (minor remark): Regarding RNA-seq data
• No additional comments.

COMMENT 14 (minor remark): Regarding what "A" tail means
• No additional comments.

COMMENT 15 (minor remark): Regarding samtools FLAG
• No additional comments.

ADDITIONAL COMMENTS:
Line 495 the authors state that "PE prediction before 12 weeks has shown limited efficacy" and they reference the DeBorre paper that evaluated PE prediction using cfDNA methylation. I don't believe their statement is supported by this reference as it's unclear what they mean by "efficacy". Certainly, earlier screening would allow for institution of interventions earlier. So why do they think that earlier screening is not "efficacious"? FMF screening can occur in the first trimester and many consider this to be "efficacious" (hence used in clinical practice). As such, I'm not sure I agree with the authors comment here as currently worded.
Author response: Thank you for your insightful comment. We agree that the FMF first-trimester screening model is widely utilized and considered clinically effective across diverse populations. We acknowledge that our original wording might have caused confusion. In our revision, we have clarified that our original statement regarding "limited efficacy" was specifically referring to the performance of cfDNA methylation-based methods, as demonstrated in the study by DeBorre et al., which reported reduced predictive performance when samples were collected before 12 weeks of gestation compared to those collected later. To avoid misunderstanding, we have revised **the first paragraph of the Discussion section** to discuss in more detail the limitations of specific molecular approaches (e.g., cfDNA methylation) and the well-established clinical value of early screening frameworks such as the FMF model.

Reviewer #4 (Remarks on code availability):
I did not review the code.
Author response: We thank the reviewer for this comment. We acknowledge that the organization and accessibility of code could be further improved. Initially, due to file size limitations encountered when using GitHub, some components (particularly large input/output files and full machine learning workflows) could not be uploaded there. To address this, we have now made all code and figure source data, including scripts for machine learning procedures and the corresponding input/output files for each figure panel, publicly available on Code Ocean

(https://codeocean.com/capsule/7028212/tree), which supports larger file sizes and offers a structured environment for reproducible research. We have also updated the “Code Availability” section in the manuscript accordingly.

REVIEWER COMMENTS

Reviewer #2 (Remarks to the Author):
1. In the interest of making the data produced in this study maximally useful for other to build on, I still think that it feasible that the authors share .bam file in anonymized form, such that fragmentomics information (start and end coordinates of each fragment) is provided, but the DNA sequence is replaced with the reference sequence, or .bed files or fragment start and end coordinates. This data can easily be generated from the same source data that has been used to generate the new analyses in this revision.
Author response: We thank the reviewer for this constructive suggestion to enhance data accessibility and reproducibility. In response, we have deposited the BED files (including sequencing read start and end coordinates) for cfDNA fragmentomics analysis in pregnant individuals to the Genome Sequence Archive (GSA) under Project ID: PRJCA035151. The dataset can be accessed via the following reviewer link: https://ngdc.cncb.ac.cn/omix/preview/joOqg60o.
We have also updated the Data Availability section of the manuscript as follows:

> *"The BED files for cfDNA fragmentomics analysis in pregnant individuals have been made available through the Genome Sequence Archive (GSA) (Project ID: PRJCA035151)."*

2. I still think that the comparison to the data from De Borre et al. is not entirely fair. The authors now write:
"Notably, our PE 566 models based on cfDNA fragmentomics alone were better than other omics-based approaches[26,37]. For example, the early-onset PE model based on cfDNA methylation alone showed lower predictive performance for samples collected before 16 weeks of gestation (AUC: 0.752)[26], compared to our early-onset PE model based on cfDNA fragmentomics alone, which achieved a higher AUC of 0.852 for samples collected at the same gestational age in test sets. "
See my original comment from the first submission: "Samples in De Borre et al. were collected 9-14 weeks of gestation, whereas this study deals with samples collected at 12-26 weeks gestation. Indeed, the De Borre et al. study mentions that collection time has a major impact on performance ("Consequently, samples obtained from week 12 onwards showed a better performance, with an AUC of 0.792 versus 0.629 for samples obtained before week 12"). Therefore, it seems this comparison is not entirely fair."
Unless I am missing something, the comparisons the authors make here is on the performance of samples 9-14 weeks (De Borre) and 12-16 weeks (their study). This is very much not the "same gestational age" – only two weeks out of a total of 8 overlap. This needs to be clarified, and the explicit claim "our PE models based on cfDNA fragmentomics alone were better than other omics-based approaches[26,37]." is not valid. This is now the third time I am asking for a fair comparison here. Care should be taken with the comparison to ref. 37 as well, which I have not evaluated in detail.
Author response: We sincerely appreciate the reviewer's thoughtful comments and for

pointing out the disparity in gestational age between our study and the De Borre et al. study. We fully agree that the previous studies, including the work by De Borre et al. and others, represent highly valuable and influential contributions that have laid a strong foundation for the development of cfDNA- and other-omics-based approaches for PE prediction. **We also acknowledge that our previous comparison was not fair, and we have now revised the text to remove such comparative language and to more accurately reflect the value and significance of these prior studies.**

Revised third paragraph of Discussion section:

> *"Although our study explored the predictive performance of PE models based on cfDNA fragmentomics for samples collected during 12-16 weeks of gestation, with an average AUC of 0.852 in test sets, we acknowledge that this performance might appear relatively higher, as clinical sampling in the participating hospitals typically occurs after 12 weeks of gestation. In contrast, De Borre et al. reported that an early-onset PE model based on cfDNA methylation alone achieved AUCs of 0.752 for samples collected between 9-14 weeks and 0.629 for those collected before 12 weeks.* ***Their study provided valuable insights into the role of cfDNA methylation in PE prediction, especially at earlier gestational ages[26].*** *Additionally, PE prediction using cfRNA profiles showed AUCs of 0.710, 0.720, and 0.740 across different validation cohorts for samples collected between 5 and 16 weeks of gestation[38].* ***Together, these studies contributed significantly to the growing body of knowledge on earlier PE detection using various omics approaches.*** *Importantly, we also appreciate the recent work by Adil et al.[36], which represents an influential contribution to the cfDNA-based PE prediction. Adil et al. reported an overall AUC of 0.84 (AUC of 0.92 for EPE and 0.79 for LPE-PB) for predicting PE requiring preterm delivery based on 0.5X pair-end sequencing data in a closely approximating real-world cohort, with samples collected at a median of 12.2 weeks (range, 8.1-16.0 weeks). Our study was in preparation and revision during the publication of their work. While our study required approximately 10X sequencing depth, which is higher than the depth used in routine NIPT workflows, it explored fragmentomic markers detectable at different sequencing depth, representing a complementary promising direction in cfDNA-based preeclampsia prediction rather than a replacement for approaches optimized for ultra-low-depth NIPT data. We believe that, for basic research and early-stage clinical investigations, examining cfDNA fragmentomic signals at different sequencing depths remains meaningful. In addition to assessing predictive performance, higher sequencing depth allows for more detailed exploration of the underlying biological mechanisms, including the relationship between predictive markers, genetic information, and infection-related signals, which might inform future marker discovery and mechanistic studies."*

3. "In Table 1, the superscript values represented the gestational age (in days) at the time of sample collection, delivery, and onset of PE. "I find this a very confusing way of visualizing the data. Consider using separate rows instead.

Author response: We thank the reviewer for this helpful suggestion. We agree that using superscript values to indicate gestational age may be confusing. To improve clarity, we have revised **Table 1, Extended Data Table1-3** by displaying gestational age at the time of sample collection, delivery, and onset of PE in keeping with De Borre et al

(PMID: 37640858). and Adil et al. studies (PMID: 39939524).

4. “we observed variability in model performance across different validation datasets when using NIPT data” -> “We observed” is confusing to me, as this sentence is just citing results from other studies. Also, this sentence and the following seem to be somewhat understating the importance of the cited studies.
Author response: We sincerely thank the reviewer for this thoughtful comment. We acknowledge that our previous phrasing (“we observed variability...”) could have caused misunderstanding, as those results were reported in other studies rather than directly observed by us. In the revised version, we focused on describing the characteristics of NIPT data in specific regions, including China, as well as our own previous study (by the same authors), which motivated our exploration of cfDNA fragmentomic features at different sequencing depths. We fully recognize that previous studies, including those by De Borre et al. and others, represent highly valuable and influential contributions that have laid a strong foundation for cfDNA- and other omics-based approaches to PE prediction. **As noted in our response to Comment 2, we have revised the third paragraph of the Discussion** to better reflect the importance of these prior studies and to avoid any language that might imply otherwise.
Revised second paragraph of Discussion section:

> *“Previous and recent studies have demonstrated the feasibility of building PE prediction models using low-depth paired-end cfDNA WGS data (0.3X or 0.5X), originally designed for fetal chromosomal abnormality screening*[10,36]*. While many current NIPT workflows have adopted or are transitioning toward paired-end sequencing, this approach has not been universally implemented across all regions. Single-end sequencing remains in use in certain clinical laboratories, such as China, which prevents estimation of fragment length. In our previous work using NIPT data routinely collected for fetal aneuploidy screening in China, predictive performance across different clinical cohorts was limited (AUCs of 0.79, 0.76, and 0.60)*[37]*, and nucleosome positioning signals around promoter regions showed insufficient resolution. These findings motivated our exploration of fragmentomic markers at different sequencing conditions and depths. In this study, we employed using a sequencing depth of at least 600 million reads (~10X final read depth) to analyze TSS coverages, TSS scores and Gini coefficients with predictive potential for PE. At this threshold, our PE models based on MFs and cfDNA fragmentomics showed robust and consistent predictive performance across test sets. For samples collected at…”*

Reviewer #4 (Remarks to the Author):

COMMENT 1-1: Regarding when were the clinical parameters such as BMI and MAP assessed?
No additional comments.

COMMENT 1-2: Regarding concerns about overfitting.

I remain confused about the first aspect of your approach – combining training and validation sets for model selection. How is this maintaining data independence? If you combined the training and validation sets, then should you not just call this your training set; and then Test Set 1 and Test Set 2 are your validation sets? Also, if you combine the training and validation sets for feature selection, how is the reader supposed to interpret Figure 4l, Figure 5 d-f, and Figure 5i?

Author response: We sincerely appreciate the reviewer's comment and the opportunity to clarify the rationale behind our dataset partitioning and terminology. In this study, the data were divided into three datasets: **training, validation**, and **two independent test sets** (Test Set 1 and Test Set 2). **The two test sets were completely independent and locked until all model development and tuning were completed**.

Following standard machine learning practice (*Goodfellow et al., Deep Learning, MIT Press, 2016; Stuart Russell and Peter Norvig, Artificial Intelligence: A Modern Approach, 2009*), <u>the training and validation datasets were used during model development, whereas the test set was strictly held out for final performance evaluation.</u> As described in the "Feature selection for each cfDNA fragmentomic" section of the Methods, **we combined the training and validation datasets for feature selection**. This process involved bootstrap-based differential analysis and a 10-fold cross-validation framework to evaluate model generalization. Although the validation data was not used to fit model parameters or to tune hyperparameters, it contributed to determining which features were informative for model input. **Therefore, the validation dataset influenced feature selection, and its performance evaluation could be somewhat biased due to "feature overfitting", justifying its designation as a validation set.** However, the **final model evaluation** was performed **entirely on the independent test sets, which were never involved in any stage of feature selection, training, or model adjustment. We thus thought our naming is appropriate and consistent with standard terminology.**

Regarding the reviewer's concern about potential overfitting, we have taken multiple steps to minimize this risk during model training, as described in our previous response. Moreover, if the AUC of the training dataset were substantially lower than that of the validation or test datasets, this would indicate the model had not adequately learned from its own data, rather than reflecting better generalization performance. As shown in Figure 4l, Figure 5d-f, and Figure 5i, **the AUC values obtained from the validation dataset (biased evaluation) and from the independent test datasets (unbiased evaluation) were highly consistent. This consistency further supports that no significant overfitting occurred in our analysis.**

To avoid confusion, we have revised the text in the **Methods section** to clarify that the independent Test Sets were used exclusively for final validation of model performance.

COMMENT 1-3: Regarding control cohort.

Thank you for this clarification, however, it remains unclear if these participants had uncomplicated pregnancies that delivered at term. The latter part about term deliveries

is important and needs to be included in the description of the cohort. Table 1 suggests that these were all term deliveries. Additionally, I would further temper your language in the Discussion as say that this “likely limits the generalizability of our findings to broader clinical populations.”
Author response: We sincerely appreciate the reviewer’s careful reading and valuable comment. We agree that clarifying the pregnancy outcomes, particularly term deliveries, is important for accurately describing the cohort. We have revised the **Methods and Discussion section** to explicitly indicate that all participants included in this study **had uncomplicated pregnancies and delivered at term**.
Regarding the Discussion, we have tempered the language as suggested and now state that this “likely limits the generalizability of our findings to broader clinical populations,” to reflect this limitation more cautiously and appropriately.

COMMENT 1-4: Regarding Figure 4j-k legend.
No additional comments.

COMMENT 2: Concerns regarding need for 10X depth when this is not routinely performed during NIPT.
Thank you for addressing this. What you’re ultimately saying, however, is that your approach does not work well on lower coverage data (below 10X). So although this exploratory approach works on 10X data, performance at lower depths is suboptimal and not comparable to the approach chosen by Adil et al. I think this remains a major and critical limitation of the novelty of your findings in light of this recent publication. You could have mitigated this by further downsampling to 0.5X to compare your results with theirs, but based on your response here, it seems that it will be inferior. Furthermore, the NIPT data used in Adil et al was paired-end sequencing, so your statement at the end of this paragraph is misleading. Overall, this aspect of the manuscript remains a significant issue.

It is also unclear what is meant by, “we observed variability in model performance across different validation datasets using NIPT data”. Do you mean that this is the performance reported in these other studies? If so, I would change the language to be clearer (not something you “observed”, but what is reported in these other papers). Also, these AUCs (0.80-0.92) are quite favorable, and comparable to your reported AUCs in your “test sets” (0.84-0.85 for early-onset PE, and 0.75-0.77 for late-onset PE). This again suggests that with higher sequencing depth, your approach does not necessarily afford any gains.
Author response: 1) We sincerely thank the reviewer for these comments. **We fully agree that the recent work by Adil et al. represents an important and influential contribution to the field. Our manuscript was in preparation and revision during the publication of that work.** As the reviewer correctly pointed out, our approach requires approximately 10X paired-end sequencing depth, which is higher than what is routinely performed in standard clinical NIPT workflows. **We acknowledge that this is a limitation of our study, and that the present model does not achieve**

comparable performance when applied to lower-depth data (e.g., 0.5X). We also appreciate the reviewer's clarification regarding sequencing formats, and we have revised the text to avoid any misleading comparison with the paired-end data used in Adil et al.

We also agree that some phrasing in our previous version may have unintentionally implied superiority over previously published studies. **In the revised Discussion section, we have removed such language and instead emphasize that our work represents a complementary direction in cfDNA-based PE prediction rather than a replacement for approaches optimized for ultra-low-depth NIPT data. We believe that, for basic research and early-stage clinical investigations, examining cfDNA fragmentomic signals at different sequencing depths remains meaningful.** In addition to assessing predictive performance, higher sequencing depth allows for more detailed exploration of the underlying biological mechanisms, including the relationship between predictive markers, genetic information, and infection-related signals, which might inform future marker discovery and mechanistic studies.

Revised third paragraph of Discussion section:

> *"Although our study explored the predictive performance of PE models based on cfDNA fragmentomics for samples collected during 12-16 weeks of gestation, with an average AUC of 0.852 in test sets, we acknowledge that this performance might appear relatively higher, as clinical sampling in the participating hospitals typically occurs after 12 weeks of gestation. In contrast, De Borre et al. reported that an early-onset PE model based on cfDNA methylation alone achieved AUCs of 0.752 for samples collected between 9-14 weeks and 0.629 for those collected before 12 weeks.* ***Their study provided valuable insights into the role of cfDNA methylation in PE prediction, especially at earlier gestational ages[26].*** *Additionally, PE prediction using cfRNA profiles showed AUCs of 0.710, 0.720, and 0.740 across different validation cohorts for samples collected between 5 and 16 weeks of gestation[38].* ***Together, these studies contributed significantly to the growing body of knowledge on earlier PE detection using various omics approaches. Importantly, we also appreciate the recent work by Adil et al.[36], which represents an influential contribution to the cfDNA-based PE prediction.*** *Adil et al. reported an overall AUC of 0.84 (AUC of 0.92 for EPE and 0.79 for LPE-PB) for predicting PE requiring preterm delivery based on 0.5X pair-end sequencing data in a closely approximating real-world cohort, with samples collected at a median of 12.2 weeks (range, 8.1-16.0 weeks). Our study was in preparation and revision during the publication of their work. While our study required approximately 10X sequencing depth, which is higher than the depth used in routine NIPT workflows, it explored fragmentomic markers detectable at different sequencing depth, representing a complementary promising direction in cfDNA-based preeclampsia prediction rather than a replacement for approaches optimized for ultra-low-depth NIPT data. We believe that, for basic research and early-stage clinical investigations, examining cfDNA fragmentomic signals at different sequencing depths remains meaningful. In addition to assessing predictive performance, higher sequencing depth allows for more detailed exploration of the underlying biological mechanisms, including the relationship between predictive markers, genetic information, and infection-related signals, which might inform future marker discovery and mechanistic studies."*

Added the limitation in Discussion section:

"Moreover, our study relied on an approximately 10X paired-end sequencing depth, which is higher than what is routinely performed in standard clinical NIPT workflows, representing a limitation compared with the study by Adil et al."

2) Moreover, we initially also expected that PE prediction could be implemented directly using existing NIPT data without additional sequencing. While many current NIPT workflows have adopted or are transitioning toward paired-end sequencing, this approach has not been universally implemented across all regions. Single-end sequencing remains in use in certain clinical laboratories, such as China, where routine NIPT testing is performed using single-end whole-genome sequencing (typically <50 bp or ~50 bp read length at ~0.1X-0.4X depth), which prevents estimation of fragment length. In our prior exploratory analyses (work by the same authors) using 0.1X NIPT data routinely used for fetal aneuploidy screening in China, predictive performance across different clinical cohorts was limited (AUCs of 0.79, 0.76, and 0.60)[37], and nucleosome positioning signals around promoter regions showed insufficient resolution. (Response Fig 1). These findings motivated our investigation into fragmentomic markers that require different sequencing depth to ensure signal stability.

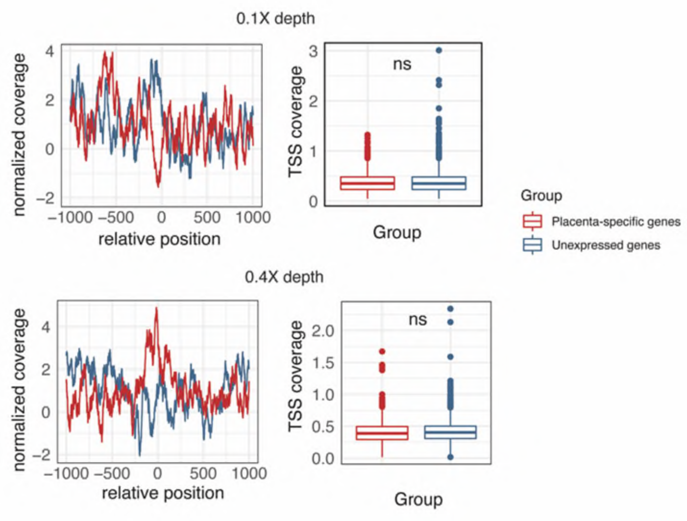

Response Fig 1. Promoter-associated nucleosome positioning signals using single-end sequencing (<50 bp read length) at 0.1X or 0.4X depth data

Revised second paragraph of Discussion section:

"Previous and recent studies have demonstrated the feasibility of building PE prediction models using low-depth paired-end cfDNA WGS data (0.3X or 0.5X), originally designed for fetal chromosomal abnormality screening[10,36]. While many current NIPT workflows have adopted or are transitioning toward paired-end sequencing, this approach has not been universally implemented across all regions. Single-end sequencing remains in use in certain clinical laboratories, such as China, which prevents estimation of fragment length. In our previous work using NIPT data routinely collected for fetal aneuploidy screening in China, predictive performance across different clinical cohorts was limited (AUCs of 0.79, 0.76, and 0.60)[37], and nucleosome positioning signals around promoter regions showed insufficient resolution. These findings motivated our exploration of fragmentomic markers at different

sequencing conditions and depths. In this study, we employed using a sequencing depth of at least 600 million reads (~10X final read depth) to analyze TSS coverages, TSS scores and Gini coefficients with predictive potential for PE. At this threshold, our PE models based on MFs and cfDNA fragmentomics showed robust and consistent predictive performance across test sets. For samples collected at…"

Regarding the control population, as discussed in our responses to previous comments (COMMENT 1-3): See comment above about whether these were all term deliveries. This needs to be added to the manuscript.
Author response: We sincerely appreciate the reviewer's careful reading and valuable comment. We agree that clarifying the pregnancy outcomes, particularly term deliveries, is important for accurately describing the cohort. We have revised the **Methods and Discussion section** to explicitly indicate that all participants included in this study **had uncomplicated pregnancies and delivered at term**.

COMMENT 3: Regarding readability of the paper.
No additional comments.

COMMENT 6: Regarding determination of a "reasonable depth for cfDNA WGS" by sequencing 50X which lead to determining that 10X was sufficient, but then ultimately using 20-40X for their study. I appreciate the changes you've made to downsample to 10X. However, I think the issue remains that your approach works well at 10X, but not lower than this. And this depth is not standard sequencing depth for NIPT. I think you need to clarify if/why your approach offers any advantage over other published studies that have performed cfDNA fragmentomics for PE risk stratification with much lower sequencing depths with comparable performance. It is not surprising that higher depth will provide higher accuracy, so the advantage of your approach is unclear.
Author response: We sincerely thank the reviewer for the comments. **As also noted in our response to Comment 2**, we acknowledge that the 10X sequencing depth in our study is higher than that typically used in clinical NIPT workflows, and this represents a limitation of our study (now clearly stated in the Discussion). **We have also revised the text to remove any potentially comparative language and to more accurately reflect the value and significance of these prior studies, including the work by Adil et al. Our manuscript was in preparation and revision during the publication of their work.**
Our goal was not to propose a directly deployable clinical test, but rather to explore cfDNA fragmentomic markers that are detectable and stable at different sequencing depths. We believe this work provides complementary value to existing ultra-low-depth NIPT studies by illustrating increased sequencing depth might improve fragmentomic signal resolution (as shown in the first section of the Result section). In our prior exploratory analyses using 0.1X NIPT data (work by the same authors)

routinely used for fetal aneuploidy screening in China, no significantly differential TSS regions were identified (adjusted p-value < 0.05). This motivated our investigate into fragmentomic markers at higher sequencing depth to ensure signal stability. Furthermore, in late-onset PE samples, we identified 1,016, 531 and 6,846 significantly differential TSS coverages, TSS scores, and Gini coefficients at 20-40X depth, compared with 107, 63 and 1,924 significantly differential TSS coverages, TSS scores, and Gini coefficients at 10X depth, indicating a reduction in detectable fragmentomic features at lower depth. While our analyses demonstrated that fragmentomic metrics, such as TSS coverage, TSS score, and Gini coefficient, showed statistically significant difference between gene expression categories at a read depth of ~10X, we acknowledged that this depth does not necessarily represent the optimal threshold for cfDNA fragmentomics analyses and predictive model development. Instead, the ~10X read depth might represent the reasonable depth required for relatively stable estimation of cfDNA fragmentomic metrics, at which biologically informative signals could be extracted and used for risk prediction (<u>clearly stated in the limitation section</u>). **For basic research and early-stage clinical investigations, we believe that exploring cfDNA fragmentomic profiles and PE predictive performance under different sequencing depth remains scientifically meaningful.** In addition to assessing predictive performance, higher sequencing depth allows for more detailed exploration of the underlying biological mechanisms, including the relationship between predictive markers, genetic information, and infection-related signals, which might inform future marker discovery and mechanistic studies.

COMMENT 11 (minor remark): Regarding TSS depth coverage
No additional comments.

COMMENT 12: Regarding diversity of cfDNA fragment lengths (Gini coefficient)
No additional comments.

ADDITIONAL COMMENTS:
The one thing I will continue to point out here, is that although you correctly report that DeBorre found suboptimal performance before 12 weeks gestation, you also did not sample before this gestational age. In fact, Table 1 indicates that your mean GA at sampling is around 16 weeks. As such, you can’t really compare your data to Deborre et al as you did not include samples from that early in gestation (or at least very few). This is also intriguing as if you propose this could be a method offered with NIPT (if sequencing costs continue to decrease), however, you mention that NIPT is routinely performed between 10-12 weeks in China. You also note that an additional blood draw at 12-16 weeks “might be challenging”, but this is what your approach would require.

Also, what do you mean by “…using samples obtained during NIPT, as well as those collected before or at 16 weeks of gestation.” For the second part, are these samples

that were not collected for NIPT? What were these samples collected for?

Author response: 1) We sincerely appreciate the reviewer's thoughtful comments and for pointing out the disparity in gestational age between our study and the De Borre et al. study. **We fully acknowledge that our previous comparison was not fair, and we would like to take this opportunity to clarify and correct this**.

We have revised the third paragraph in the "*Discussion*" section, and the updated text now reads as follows:

> *"Although our study explored the predictive performance of PE models based on cfDNA fragmentomics for samples collected during 12-16 weeks of gestation, with an average AUC of 0.852 in test sets, we acknowledge that this performance might appear relatively higher, as clinical sampling in the participating hospitals typically occurs after 12 weeks of gestation. In contrast, De Borre et al. reported that an early-onset PE model based on cfDNA methylation alone achieved AUCs of 0.752 for samples collected between 9-14 weeks and 0.629 for those collected before 12 weeks.* ***Their study provided valuable insights into the role of cfDNA methylation in PE prediction, especially at earlier gestational ages***[26]*. Additionally, PE prediction using cfRNA profiles showed AUCs of 0.710, 0.720, and 0.740 across different validation cohorts for samples collected between 5 and 16 weeks of gestation*[38]*.* ***Together, these studies contributed significantly to the growing body of knowledge on earlier PE detection using various omics approaches. Importantly, we also appreciate the recent work by Adil et al.***[36]***, which represents an influential contribution to the cfDNA-based PE prediction.*** *Adil et al. reported an overall AUC of 0.84 (AUC of 0.92 for EPE and 0.79 for LPE-PB) for predicting PE requiring preterm delivery based on 0.5X pair-end sequencing data in a closely approximating real-world cohort, with samples collected at a median of 12.2 weeks (range, 8.1-16.0 weeks). Our study was in preparation and revision during the publication of their work. While our study required approximately 10X sequencing depth, which is higher than the depth used in routine NIPT workflows, it explored fragmentomic markers detectable at different sequencing depth, representing a complementary promising direction in cfDNA-based preeclampsia prediction rather than a replacement for approaches optimized for ultra-low-depth NIPT data. We believe that, for basic research and early-stage clinical investigations, examining cfDNA fragmentomic signals at different sequencing depths remains meaningful. In addition to assessing predictive performance, higher sequencing depth allows for more detailed exploration of the underlying biological mechanisms, including the relationship between predictive markers, genetic information, and infection-related signals, which might inform future marker discovery and mechanistic studies."*

2) We thank the reviewer for highlighting the potential confusion regarding sample collection timing. We acknowledge that our previous description may have been unclear, which could have led to misinterpretation. In China, the routine first-trimester blood test is conducted between 10 and 12 weeks of gestation, primarily for basic maternal health assessment (including ECG, blood counts, urine analysis, liver/kidney function, thyroid function, glucose, and anthropometrics). This routine blood draw is not for NIPT. **NIPT sampling is typically performed between 12 and 26 weeks of gestation, which aligns with our study samples.** A small subset of these NIPT samples were collected before 16 weeks, representing the early portion of this sampling window. If

PE risk prediction were restricted to only this early window (< 16 weeks), many high-risk pregnancies could potentially be overlooked. Therefore, the primary analysis relied on the broader NIPT sampling period (12-26 weeks). The samples collected before or at 16 weeks of gestation represented a subset of this NIPT sampling window (12-16 weeks). We have revised the first paragraph in the "*Discussion*" section as follows:

> *"While Non-invasive prenatal testing (NIPT) for fetal aneuploidy screening can be performed from ≥10 weeks of gestation, according to American College of Medical Genetics and Genomics (ACMG) guidelines[34], in China, routine first-trimester blood tests are typically conducted between 10 and 12 weeks for maternal health assessment, and NIPT is generally performed between 12 and 26 weeks. Within this window, only a small proportion of samples were collected before 16 weeks, and restricting PE prediction to this early subset could potentially overlook many high-risk pregnancies. Given that the American College of Obstetricians and Gynecologists (ACOG) recommends starting low-dose aspirin between 12 and 28 weeks of gestation, and that NIPT is typically performed during this window, it is clinical meaningful to explore whether cfDNA-based methods could provide predictive value using routinely collected NIPT samples. Therefore, in this study, we evaluated the performance of PE prediction based on cfDNA fragmentomics using samples obtained during NIPT, as well as the subset of NIPT samples collected before or at 16 weeks of gestation."*
